# Therapeutic effects on the development of heart failure with preserved ejection fraction by the sodium-glucose cotransporter 2 inhibitor dapagliflozin in type 2 diabetes

**DOI:** 10.1186/s13098-023-01116-8

**Published:** 2023-06-29

**Authors:** Bin Feng, Peiran Yu, Hao Yu, Buyun Qian, Yuan Li, Kangyun Sun, Bimin Shi, Nannan Zhang, Guidong Xu

**Affiliations:** 1 Department of Endocrinology and Metabolism, The First Affiliated Hospital of Soochow University, Suzhou, 215006 Jiangsu People’s Republic of China; 2 Department of Cardiology, The Affiliated Suzhou Hospital of Nanjing Medical University, 242 Guangji Road, Suzhou, 215008 Jiangsu People’s Republic of China

**Keywords:** Dapagliflozin, Diabetic cardiomyopathy, Heart failure with preserved ejection fraction, AMPK/mTOR, Multi-omic analysis

## Abstract

**Background:**

Heart failure with preserved ejection fraction (HFpEF) is a common disease with high morbidity and lacks effective treatment. We investigated the protective effects of the long-term application of the sodium-glucose cotransporter 2 inhibitor (SGLT2i) dapagliflozin on diabetes-associated HFpEF in a rat model. Serum proteomics and metabolomics analysis were also conducted in type 2 diabetic patients with HFpEF treated with dapagliflozin.

**Methods:**

Male Zucker diabetic fatty (ZDF) rats were used as a model of diabetic cardiomyopathy. From weeks 16 to 28, animals were given a vehicle or dapagliflozin (1 mg/kg) once daily. Primary blood biochemistry indices, echocardiography, histopathology, and cardiac hemodynamics were determined during the study period. The key markers of myocardial fibrosis, nitro-oxidative stress, inflammation, apoptosis, autophagy, and AMPK/mTOR signaling were examined. Additionally, healthy controls and individuals with type 2 diabetes were enrolled and 16 serum samples from 4 groups were randomly selected. Serum proteome and metabolome changes after dapagliflozin treatment were analyzed in diabetic individuals with HFpEF.

**Results:**

Dapagliflozin effectively prevented the development of HFpEF in rats with diabetes by mitigating nitro-oxidative stress, pro-inflammatory cytokines, myocardial hypertrophy, and fibrosis, reducing apoptosis, and restoring autophagy through AMPK activating and mTOR pathway repressing. Proteomics and metabolomics revealed that cholesterol and high-density lipoprotein particle metabolism, nicotinate and nicotinamide metabolism, arginine biosynthesis, and cAMP and peroxisome proliferator-activated receptor (PPAR) signaling are the major disturbed pathways in HFpEF patients treated with dapagliflozin.

**Conclusion:**

Long-term treatment with dapagliflozin significantly prevented the development of HFpEF in diabetic rats. Dapagliflozin could be a promising therapeutic strategy in managing HFpEF individuals with type 2 diabetes.

**Supplementary Information:**

The online version contains supplementary material available at 10.1186/s13098-023-01116-8.

## Background

According to the epidemiological data released by International Diabetes Federation (IDF), the global diabetes prevalence in 2021 is estimated to be 10.5% (537 million people), rising to 11.3% (643 million) by 2030 and 12.2% (783 million) by 2045 [1]. Cardiovascular complications are the primary cause of mortality and morbidity in diabetic patients [2]. Furthermore, the clinical outcomes associated with heart failure (HF) are considerably worse for patients with diabetes than those without diabetes [3]. However, conventional therapies for established heart failure are the same, whether or not the patient has diabetes, which means mechanism-specific therapy for diabetes-associated heart failure is currently unavailable [4]. Diabetic cardiomyopathy is a common diabetic cardiovascular complication characterized by myocardial fibrosis, dysfunctional remodeling, and associated diastolic dysfunction, later by systolic dysfunction, and eventually by clinical heart failure, which occurs independently of other cardiac risk factors such as coronary artery disease and hypertension [5, 6]. In general, initial impairments in left ventricular (LV) diastolic function are thought to be typical of diabetes-induced cardiomyopathy, manifested as heart failure with preserved ejection fraction (HFpEF) [7]. In type 2 diabetes (T2DM)-associated HFpEF, metabolic disorders like hyperglycemia, hyperlipidemia, and insulin resistance in the heart cause mitochondrial dysfunction in cardiomyocytes and endothelial cells, inducing fibrosis, oxidative stress, inflammation, endoplasmic reticulum stress, and myocardial apoptosis [8–11]. Although several molecular mechanisms for the development of HFpEF have been identified [12–14], they are still only partially understood. More importantly, therapies that target diabetes-induced cardiac dysfunction are urgently needed.

Sodium-glucose cotransporter 2 inhibitors (SGLT2is) are newly developed agents for the treatment of T2DM, which increase urinary excretion of glucose via SGLT2 inhibition in the kidney and thereby prevent hyperglycemic episodes in diabetic animals and individuals [15]. Besides EMPA-REG OUTCOME, CANVAS and DECLARE three large randomized trials [16–18], recently published DAPA-HF and EMPEROR-Reduced trials [19, 20] have shown that SGLT2is (empagliflozin, canagliflozin or dapagliflozin) reduced the cardiovascular deaths or hospitalization for heart failure more than the placebo group, regardless of the presence or absence of diabetes in heart failure with reduced ejection fraction (HFrEF) patients. Furthermore, a prospective multicenter trial showed the beneficial effect of dapagliflozin on left ventricular diastolic functional parameters for 58 T2DM patients with heart failure [21]. In addition, dapagliflozin has recently been shown to reduce the combined risk of worsening heart failure or cardiovascular death among HFpEF patients [22]. SGLT2i has been found to be cardioprotective in animal models by improving metabolic disorders and pathological process such as insulin resistance, obesity, inflammation, oxidative stress, fibrosis, and apoptosis [23–25]. Our previous study described dapagliflozin’s anti-inflammatory, anti-structure remodeling, and vascular-protective effects of in hypertension-induced HFpEF pigs [26]. Similarly, Santos-Gallego et al. showed that empagliflozin could switch myocardial fuel utilization, thereby improving myocardial energetics and ameliorating adverse left ventricular remodeling in nondiabetic pigs [27]. Despite such promising results, it is still unclear whether SGLT2is is beneficial for left ventricular diastolic dysfunction and the underlying mechanisms in T2DM- associated HFpEF.

The present study investigated whether long-term administration of the SGLT2i dapagliflozin in the prediabetic phase could prevent the development of HFpEF in Zucker diabetic fatty (ZDF) rats and tried to explore the potential molecular mechanism.

## Materials and methods

### Animals and experimental protocol

After 1 week of acclimation, 6 weeks-old male ZDF diabetic (fa/fa) and ZDF lean (fa/+) rats (Vital River Laboratory Animal Technology Co., Ltd., Beijing, China) were assigned to three groups: (a) Lean controls, dosed orally with vehicle (1 mL/kg/day, n = 8); (b) ZDF, treated with vehicle (1 mL/kg/day, n = 8); (c) ZDF + Dapa, treated with dapagliflozin (1 mg/kg/day, n = 8) from weeks 16 to 28. Rats were housed at a constant temperature (22 ± 2 °C) and 50% humidity on a 12 h light/dark cycle. ZDF rats were maintained on Purina 5008 chow and water ad libitum. At the end of 15 weeks, ZDF rats with fasting blood glucose ≥ 11.1 were considered to have successfully induced T2DM and were further divided into two groups for subsequent drug intervention experiments. The drug or vehicle was administered once daily through drinking water, beginning at 16 weeks of age and continued until the end of the study. Body weight (BW) was measured every week and the dosage of dapagliflozin was adjusted accordingly. Blood pressure (BP) was recorded noninvasively using the tail-cuff method, and readings were averaged from at least 3 measurements. Functional measurements were taken at the age of 28 weeks. All animals were euthanized in a random fed state. Then heart tissues were immediately dissected, fixed with 10% formaldehyde or snap-frozen in liquid nitrogen, and stored at − 80 °C until use.

### Clinical study design

Outpatients or inpatients aged 35–75 years were enrolled at the Department of Cardiology of the Affiliated Suzhou Hospital of Nanjing Medical University from June 2020 to June 2021, including normal controls (NC group), individuals with type 2 diabetes (DM group), DM with HFpEF (DM-HF group), and DM-HF treated with dapagliflozin (DAPA group). T2DM was diagnosed using the World Health Organization criteria of 1999. HFpEF was defined using the following criteria [28]: (i) Patients with symptomatic HF; (ii) plasma N-terminal pro-B-type natriuretic peptide (NT-proBNP) ≥ 125 ng/L or BNP ≥ 35 pg/L and (iii) echocardiographic measured ejection fraction (EF) ≥ 50%. Patients in the NC group had neither DM nor HFpEF. In the DAPA group, dapagliflozin (10 mg once daily) had been used for more than 3 months.

Key exclusion criteria included: type 1 diabetes; coronary heart disease; chronic atrial fibrillation; stroke; severe hepatic and renal dysfunction; systolic blood pressure ≥ 160 mmHg if not on three or more antihypertensive medications, or ≥ 180 mmHg regardless of the number of medications; uncorrected primary valvular disease; known or suspected amyloid heart disease; myo- or pericarditis; hypertrophic cardiomyopathy; probable alternative diagnoses that might account for the patient's symptoms (e.g., anemia, hypothyroidism, primary pulmonary hypertension).

### Biochemistry tests

Fasting blood glucose (FBG) levels were measured by a Glucometer (Roche) at indicated time points during the study. Venous blood samples were collected after a hemodynamic study at the age of 28 weeks. Plasma glucose, glycated hemoglobin (HbA1c), creatinine, atrial natriuretic peptide (ANP), NT-proBNP, triglyceride (TG), low-density lipoprotein-cholesterol (LDL-C), and high-density lipoprotein-cholesterol (HDL-C) were measured using Roche Diagnostics System (Madison, WI, USA). After dosing with vehicle or dapagliflozin to determine glucosuria, rats were immediately placed into metabolism cages for 24 h urine collection. Quantitative assays of urine glucose were performed by using the Cobas Mira Analyzer. Urine glucose data were normalized per 200 g body weight. A commercially available ELISA kit was used for serum 3-NT determination. The activity of superoxide dismutase (SOD), catalase (CAT), glutathione peroxidase (GPx), and the concentration of malondialdehyde (MDA) in heart homogenate was determined by commercial assay kits according to the manufacturer’s (Jiancheng Bioengineering Institute, Nanjing, China).

### Intraperitoneal glucose tolerance test (IPGTT)

For the glucose tolerance test, rats were fasted overnight and dosed intraperitoneally with glucose solution (2 g/kg in saline). Blood glucose levels were measured with a glucometer before (0 min) and 30, 60, 90, and 120 min after glucose administration. The delta area under the curve for baseline glucose levels was calculated.

### Echocardiography

At the age of 28 weeks, rats underwent a standard transthoracic echocardiogram examination. Echocardiography was conducted using an echocardiographic system (Vivid q, GE Vingmed, Horten, Norway). Standard 2D and M-mode were performed to measure the interventricular septum thickness at end-diastole (IVSd), LV anterior (LVAW) and posterior wall (LVPW) thickness, LV mass, LV internal diameter at end-diastole (LVIDd) and systole (LVIDs). Relative wall thickness (RWT) was calculated, and LV mass was normalized to the body weight (LVmass index) and the tibia length (LVmass/TL). Pulse-wave Doppler was harnessed to measure isovolumetric relaxation time (IVRT), deceleration time (DT), mitral inflow E wave and mitral inflow A wave. Tissue Doppler was used to measure median mitral annular early diastolic velocity (e'), followed by calculating of E/e' and E/A. Finally, the average value of three consecutive beats was calculated. All analyses were completed by an independent investigator on a workstation (EchoPac-PC, version 6.1.0, Vingmed-General Electric, Horton, Norway).

In the clinical study, echocardiography was performed with commercially available equipment (iE33, Philips, Amsterdam, Netherlands) in all participants. Standard echocardiographic parameters were obtained in accordance with the guidelines of the American Society of Echocardiography.

### Invasive hemodynamic assessment

After the chronic treatment period, invasive hemodynamic measurements were conducted under general anesthesia to assess left ventricular function. A 2F microtip pressure-conductance microcatheter (SPR-838, Millar Instruments, Houston, USA) was inserted into the right carotid artery. A P–V conductance system (Millar instrument, Houston, USA) was used to record all pressure–volume (P–V) loop data. A commercially available programme (PVAN, Millar Instruments, Houston, USA) was used to calculate mean arterial pressure (MAP), ejection fraction (EF), cardiac output (CO), the maximal slope of systolic pressure increment (dP/dt_max_), and diastolic pressure decrement (dP/dt_min_), and time constant of LV pressure decay (Tau_w_). The slope (Ees) of the LV end-systolic pressure–volume relationship (ESPVR) and preload recruitable stroke work (PRSW) were calculated as indices of LV contractility, and the slope of the LV end-diastolic pressure–volume relationship (EDPVR) was calculated as an index of LV diastolic stiffness. Rats were quickly exsanguinated, and their hearts were immediately excised. Then Tibia length (TL) and heart weight (HW) were measured.

### Histology and TUNEL assay

Five μm thick myocardial sections were deparaffinized and stained with hematoxylin–eosin (HE) to assess general morphology. Cardiomyocyte diameter was measured and normalized to TL. Masson's trichrome and picrosirius red staining was performed to evaluate the extent of fibrotic remodeling by conventional methods. Images were obtained using a Zeiss microscope (Carl Zeiss, Germany), and 8 fields of each section were randomly selected in a blinded fashion. Positive staining areas were determined by Image J (NIH, Bethesda, MD, USA), and the average percentage was calculated relative to the total area of the tissue.

For apoptosis determination, tissue sections were subjected to terminal deoxynucleotidyl transferase dUTP nick-end labeling (TUNEL) analysis using a commercially available detection kit (R&D Systems, Minneapolis, Minnesota, USA) according to the manufacturer's instruction. TUNEL-positive cells were imaged by a fluorescence microscope (Nikon Eclipse E800, Tokyo, Japan) and counted in 8 randomly selected fields of each section.

### Quantitative real-time PCR

Briefly, total RNA was extracted from LV tissues using Trizol reagent and reverse transcribed into cDNA using a High-capacity cDNA Reverse Transcription kit according to the manufacturer's instructions (Invitrogen, Carlsbad, CA, USA). qRT-PCR was performed on a StepOnePlus™ PCR System (Applied Biosystems, Foster City, CA, USA). Relative expression levels of each target gene to the internal control were calculated by the 2^−∆∆CT^ method. The primer sequences used in the study are listed in Additional file 6: Table S1.

### Western blot analysis

Preparation of protein samples and Western blot analyses were performed as previously described [29]. Briefly, the LV sample homogenates were added with a lysis buffer and heated at 95 °C for 2 min. Sample aliquots were subjected to sodium dodecyl sulfate–polyacrylamide gel electrophoresis (SDS-PAGE) and transferred onto polyvinylidene difluoride (PVDF) membranes (Millipore-Linco, St Charles, MO) for Western blot. The proteins were detected using the following primary antibodies: anti-β-actin (1:3000, Bioworld Technology), anti-P65 (1:2000, Abcam, ab16502), anti-p-P65 (1:500, ab76302), anti-IkBɑ (1:1000, BioVision, 3252), anti-cleaved PARP-1 (1:500, Invitrogen, 44-698G), anti-total PARP-1 (1:1500, Invitrogen, PA5-16452), anti-B-cell lymphoma/leukaemia-2 (Bcl-2) (1:1000, Affinity, AF6139), anti-AMPK (1:2000, PA5-105297), anti-p-AMPK (1:500, PA5-37821), anti-mTOR (1:2000, ab134903), anti-p-mTOR (1:500, ab109268), anti-p62 (1:1500, PA5-20839), and anti-LC3-I/II (1:1000, PA5-22731). The bound antibodies were detected with horseradish peroxidase (HRP)-conjugated secondary antibodies for 1 h at room temperature and visualized using enhanced chemiluminescence (ECL, Millipore-Linco, St Charles, MO). Band densities were quantified using Image J software. All protein expression was normalized by β-actin as an internal control.

### Serum proteomic sample preparation and LC–MS/MS analysis

A total of 105 individuals were enrolled, including 30 normal controls (NC), 30 people with type 2 diabetes (DM), 25 DM with HFpEF (DM-HF), and 20 DM-HF treated with dapagliflozin (DAPA). For each group, 16 serum samples were randomly selected. Sets of 4 serum samples were then mixed into 1 loading sample, resulting in 4 loading samples per group. First, serum pools were depleted of abundant proteins using Multiple Affinity Removal Column Human 14 (Agilent, USA). Then, after determining the concentration by the bicinchoninic acid (BCA) method, one part of each sample was used for SDS-PAGE. In addition, 50 μg protein of each sample was digested with trypsin and peptide mixtures were labeled with tandem mass tags reagent (TMT) according to the manufacturer's instructions (Thermo Scientific, USA).

Protein identification, quantitation, and proteomics data analysis were performed by Luming Biological Technology (Shanghai, China). In addition, Gene Ontology (GO) term and Kyoto Encyclopedia of Genes and Genomes (KEGG) pathway analysis was used for the biological categorization of proteins that were significantly differentially expressed in the serum of subjects from different groups. The Additional file 1: Methods provide more detailed descriptions of the methodology.

### Serum sample preparation and nontargeted GC–MS analysis

Serum samples stored at − 80 °C were thawed to 4 °C. Each aliquot was supplemented with 20 μL of L-2-chlorophenylalanine (0.3 mg/mL) dissolved in methanol as internal standard, and the tube was vortexed for 10 s. Subsequently, 900 μL cold methanol/water (8:1 vol/vol) was added, and the mixtures were extracted by 4 min ultrasonication and 30 s vortex-mixing in an ice-water bath. The extract was centrifuged at 4 °C (13,000 rpm) for 10 min. Next, 500 μL of supernatant in a glass vial was dried in a freeze-concentration centrifugal dryer. The dried aliquots were combined with 20 μL of Myristic-d27 acid as internal standard and 50 μL of methoxyamine hydrochloride in pyridine (15 mg/mL), and then vortexed for 2 min and shaken at 37 °C for 90 min. Finally, 30 μL of BSTFA (with 1% TMCS) were added to the mixture, vortexed vigorously for 2 min, and then derivatized at 70 °C for 60 min. QC samples were prepared by pooling aliquots of all the serum samples and were processed with the same procedure.

The derivatized samples were analyzed using a GC–MS system (Agilent Technologies Inc., CA, USA). The raw data analysis was performed by Luming Biological Technology (Shanghai, China). Principle Component Analysis (PCA) was carried out to observe the overall distribution among the samples. Partial least squares -discriminant analysis (PLS-DA) was used to distinguish the metabolites that differ between groups. The variables were selected based on the variable importance of projection (VIP > 1.0) from the peak height. In addition to the multivariate statistical method, a two-tailed Student's t-test was further used to verify whether the metabolites of difference between groups were significant. The resultant P values from ANOVA were further adjusted by the false discovery rate (FDR). Significantly altered variables were selected with VIP > 1.0, P < 0.05, and FDR < 0.05.

### Statistical analysis

All data were expressed as means ± SD. Normal distribution was tested by the Shapiro-Wilks method. For non-normally distributed variables, a logarithmic transformation was carried out before further analysis. We employed Student's t-test and One-way ANOVA followed by the Bonferroni post hoc test to examine intergroup differences. In addition, the two-way ANOVA was performed using the factors “T2DM” and “Dapagliflozin”. Correlation analysis was performed on two groups of data that were continuous, normally distributed, and had a linear relationship using the Pearson test, otherwise the Spearman test was used. SPSS 22.0 was used for the statistical analyses, while the GraphPad Prism 8.0 software was used to draw graphs. p < 0.05 was deemed statistically significant.

## Results

### Dapagliflozin alleviated metabolic disorders in ZDF rats

**Fig. 1:**
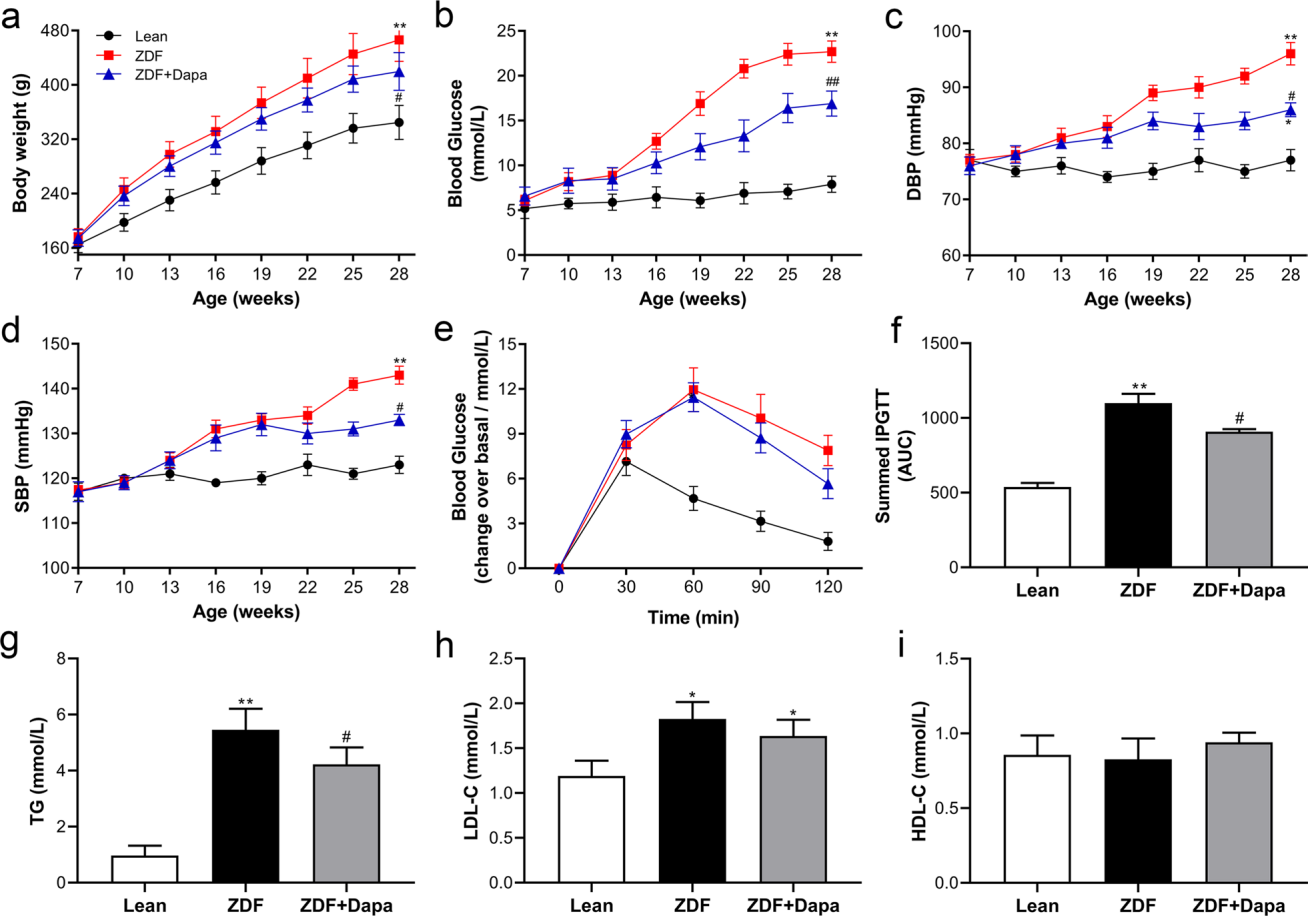
The effects of dapagliflozin on the metabolic characteristics in ZDF rats. **a** Changes in body weight. **b** Random blood glucose. **c**, **d** Diastolic and systolic blood pressure. **e**, **f** Intraperitoneal glucose tolerance tests were performed, and the area under the curve was calculated. **g**–**i** The serum lipid profile of all rats. The data are presented as the mean ± SD. n = 8 per group. *P < 0.05, **P < 0.01 vs. Lean; ^#^*P* < 0.05, ^##^*P* < 0.01 vs. ZDF

At the end of the study, rats in the ZDF group showed significantly increased BW, FBG, and BP compared with lean rats (Fig. 1a–d). In addition, a marked difference in the AUC of IPGTT was observed between the ZDF and Lean group, suggesting an impaired glucose tolerance (Fig. 1e, f), which, together with an abnormal lipid profile (Fig. 1g–i) constitutes metabolic disorders in ZDF rats. Nevertheless, these changes in metabolic indexes, including FBG, HbA1c, 24-h urine glucose quantification (Table 1), BP, the AUC of IPGTT, and TG (Fig. 1c–g) were significantly reduced with 12 weeks of dapagliflozin treatment.

**Table 1:** Metabolic characteristics, echocardiographic and haemodynamic parameters of experimental rats

Variable	Lean	ZDF	ZDF + Dapa
Metabolic characteristics			
BW (g)	344.8 ± 25.1	466.3 ± 31.6*	419.8 ± 27.9^#^
HW (g)	1.25 ± 0.12	1.37 ± 0.13	1.35 ± 0.13
HW/BW (g/kg)	3.32 ± 0.17	3.39 ± 0.19	3.25 ± 0.24
HW/TL (g/cm)	0.322 ± 0.014	0.353 ± 0.018*	0.344 ± 0.017*
FBG (mmol/L)	6.5 ± 0.8	18.4 ± 2.6*	11.3 ± 1.7^#^
HbA1c (%)	5.1 ± 0.9	15.8 ± 3.2*	10.4 ± 2.7^#^
24 h urine glucose (mg/200 g BW)	2.8 ± 0.5	275.4 ± 21.1*	4790.4 ± 746.7^#^
Creatinine (mg/dl)	0.72 ± 0.13	0.65 ± 0.15	0.63 ± 0.11
ANP (pg/mL)	82.4 ± 21.0	213.5 ± 56.3*	188.2 ± 54.9*
NT-proBNP (pg/mL)	253.5 ± 62.7	863.3 ± 127.5*	475.2 ± 112.3^#^
Echocardiographic parameters			
LVIDs (mm)	7.73 ± 0.58	7.74 ± 0.66	7.68 ± 0.67
LVIDd (mm)	11.87 ± 0.67	11.74 ± 0.71	11.61 ± 0.78
LVAWs (mm)	2.30 ± 0.15	2.71 ± 0.17*	2.46 ± 0.12^#^
LVAWd (mm)	1.59 ± 0.09	1.75 ± 0.07*	1.70 ± 0.05
LVPWs (mm)	2.49 ± 0.11	2.64 ± 0.15	2.67 ± 0.13
LVPWd (mm)	1.74 ± 0.09	1.86 ± 0.11*	1.77 ± 0.09
RWT	0.41 ± 0.03	0.45 ± 0.05	0.43 ± 0.05
LVmass (g)	0.90 ± 0.03	0.95 ± 0.05	0.96 ± 0.06
LVmass/TL (g/cm)	0.22 ± 0.01	0.27 ± 0.02*	0.24 ± 0.01
LVmass index (g/kg)	2.38 ± 0.18	2.79 ± 0.22*	2.68 ± 0.20
IVSd (mm)	2.59 ± 0.13	2.76 ± 0.15	2.70 ± 0.13
E/e'	2.17 ± 0.18	2.31 ± 0.19*	2.26 ± 0.21^#^
E/A	1.71 ± 0.12	1.63 ± 0.12*	1.65 ± 0.11
DT (ms)	62.1 ± 10.1	76.8 ± 11.9*	75.6 ± 10.6
IVRT (ms)	31.2 ± 1.6	34.9 ± 1.9*	32.6 ± 1.3^#^
Haemodynamic parameters			
HR (bpm)	321 ± 19	328 ± 17	330 ± 16
MAP (mmHg)	95 ± 8	106 ± 11*	98 ± 7^#^
EF (%)	76.9 ± 3.3	74.8 ± 2.7	77.9 ± 3.7
CO (mL/min)	70 ± 6	68 ± 7	70 ± 6
dP/dt_max_ (mmHg/s)	4042 ± 324	3881 ± 202	4167 ± 263
dP/dt_min_ (mmHg/s)	− 3366 ± 252	− 2857 ± 194*	− 3095 ± 271^#^

Values are expressed as the mean ± SD. n = 8 per group. *P < 0.05 vs. Lean; ^#^*P* < 0.05 vs. ZDF. HW, heart weight; TL: tibia length; AW: anterior wall thickness; PW: posterior wall thickness; LVID: LV internal diameter; RWT: relative wall thickness; IVSd: inter ventricular septum at end-diastole; DT: deceleration time; IVRT: iso-volumetric relaxation time; MAP: mean arterial pressure; EF: ejection fraction; CO: cardiac output; dP/dt_max_ and dP/dt_min_: maximal and minimal slope of dP/dt. The ‘s’ and ‘d’ after the acronyms indicate end-systolic and end-diastolic, respectively

### Dapagliflozin improved left ventricular dysfunction and myocardial remodeling in ZDF rats

**Fig. 2:**
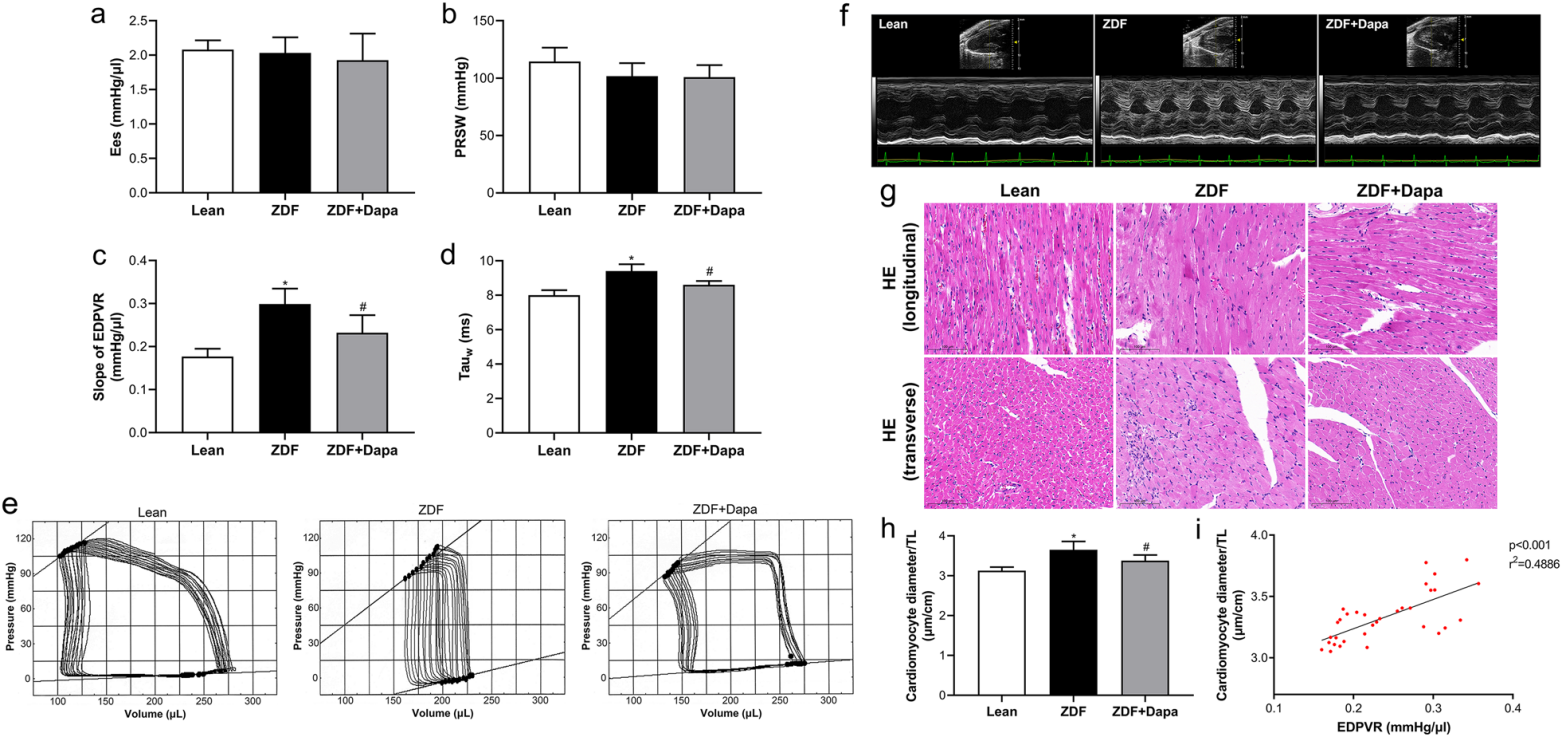
Dapagliflozin improved left ventricular diastolic function and myocardial hypertrophy in ZDF rats. **a**–**d** Key parameters obtained from invasive left cardiac catheterization. **e** Representative LV pressure–volume (P–V) loops and the slope of EDPVR. **f** Representative images of M-mode echocardiography. **g** HE staining of heart sections is presented. Bar = 100 μm, Magnification = ×40. **h** The cardiomyocyte diameter/TL ratio was calculated. **i** Correlation analysis between cardiomyocyte diameter/TL and the slope of EDPVR. Data are the mean ± SD. n = 8 per group. *P < 0.05 vs. Lean; ^#^*P* < 0.05 vs. ZDF

In order to explore cardiac function, several classic measurements were performed by biochemistry tests, invasive left cardiac catheterization, and echocardiography. Increased plasma ANP and NT-proBNP levels were noted in ZDF rats, while only the latter was significantly reduced in response to dapagliflozin treatment (Table 1). Conventional systolic parameters, such as EF, CO, and dP/dt_max_, did not differ among the three study groups (Table 1). Similarly, indexes of LV contractility, such as Ees and PRSW, remained unchanged (Fig. 2a, b). However, we found that EDPVR slope and Tau_w_ were significantly elevated in ZDF (Fig. 2c, d), prolonged DT and IVRT, increased E/e’, and decreased dP/dt_min_ and E/A (Table 1), demonstrating an impaired LV diastolic function in vivo. Dapagliflozin treatment markedly blunted the slope of EDPVR and Tau_w_. Meanwhile, IVRT, dP/dt_min_, and E/e’ were improved in ZDF rats. Representative diagrams of EDPVR are shown in Fig. 2e. In ZDF rats, echocardiography revealed significant increase in LVAW, LVPW, and LVmass/TL, indicating in vivo myocardial hypertrophy. However, LVAWs and LVPWd were statistically decreased in response to dapagliflozin treatment (Table 1).

**Fig. 3:**
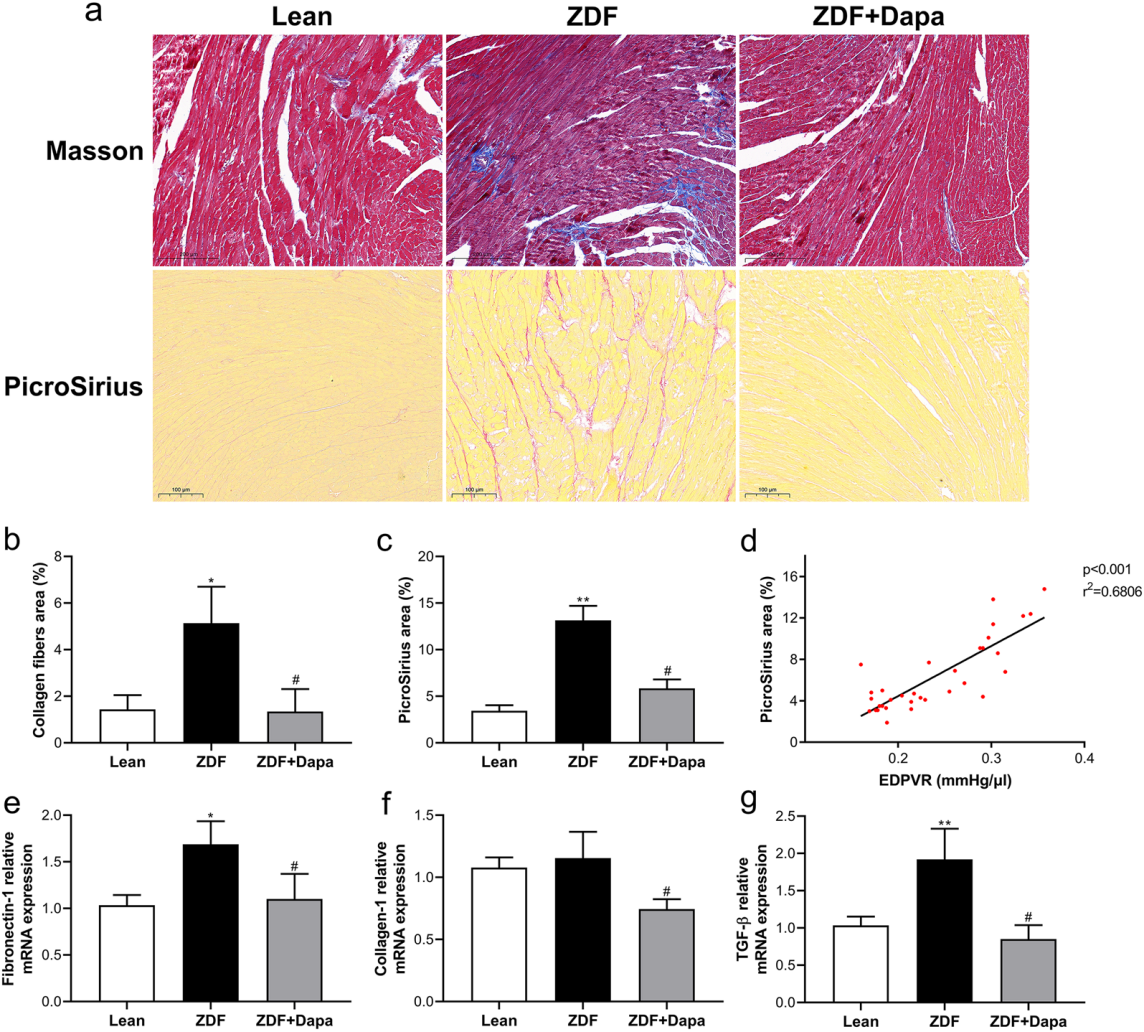
The effects of dapagliflozin on myocardium fibrosis in diabetic rats. **a** Representative images of Masson's trichrome and PicroSirius staining. Bar = 200 and 100 μm, respectively. Magnification = ×20. **b**, **c** Fibrotic area quantification of stained sections. **d** Correlation analysis between PicroSirius positive area and the slope of EDPVR. **e**–**g** Quantitative real-time PCR was conducted to detect the mRNA expression of Fibronectin-1, Collagen-1, and TGF-β in the myocardium. Values are expressed as the mean ± SD. n = 8 per group. *P < 0.05, **P < 0.01 vs. Lean; ^#^*P* < 0.05 vs. ZDF

Representative M-mode echocardiography images are presented in Fig. 2f. HE staining of heart sections revealed typical cardiac hypertrophy with increased cardiomyocyte diameter in ZDF, effectively diminished by dapagliflozin (Fig. 2g, h). In addition, the slope of EDPVR was positively associated with cardiomyocyte diameter/TL (Fig. 2i). Fibrotic remodeling of the myocardium was assessed by Masson trichrome and PicroSirius red staining. As presented in Fig. 3a–d, myocardial fibrosis was aggravated in diabetic rats, and the extent of fibrosis correlated robustly with the slope of EDPVR. However, this pathological change was strongly suppressed in the ZDF + Dapa group. In addition, diabetes-induced higher mRNA levels of Fibronectin-1, Collagen-1, and TGF-β were markedly inhibited with dapagliflozin, even though the gene expression of Collagen-1 was not changed in ZDF rats as compared with lean controls (Fig. 3e–g).

### Dapagliflozin prevented diabetes-associated myocardial nitro-oxidative stress and inflammation in ZDF rats

**Fig. 4:**
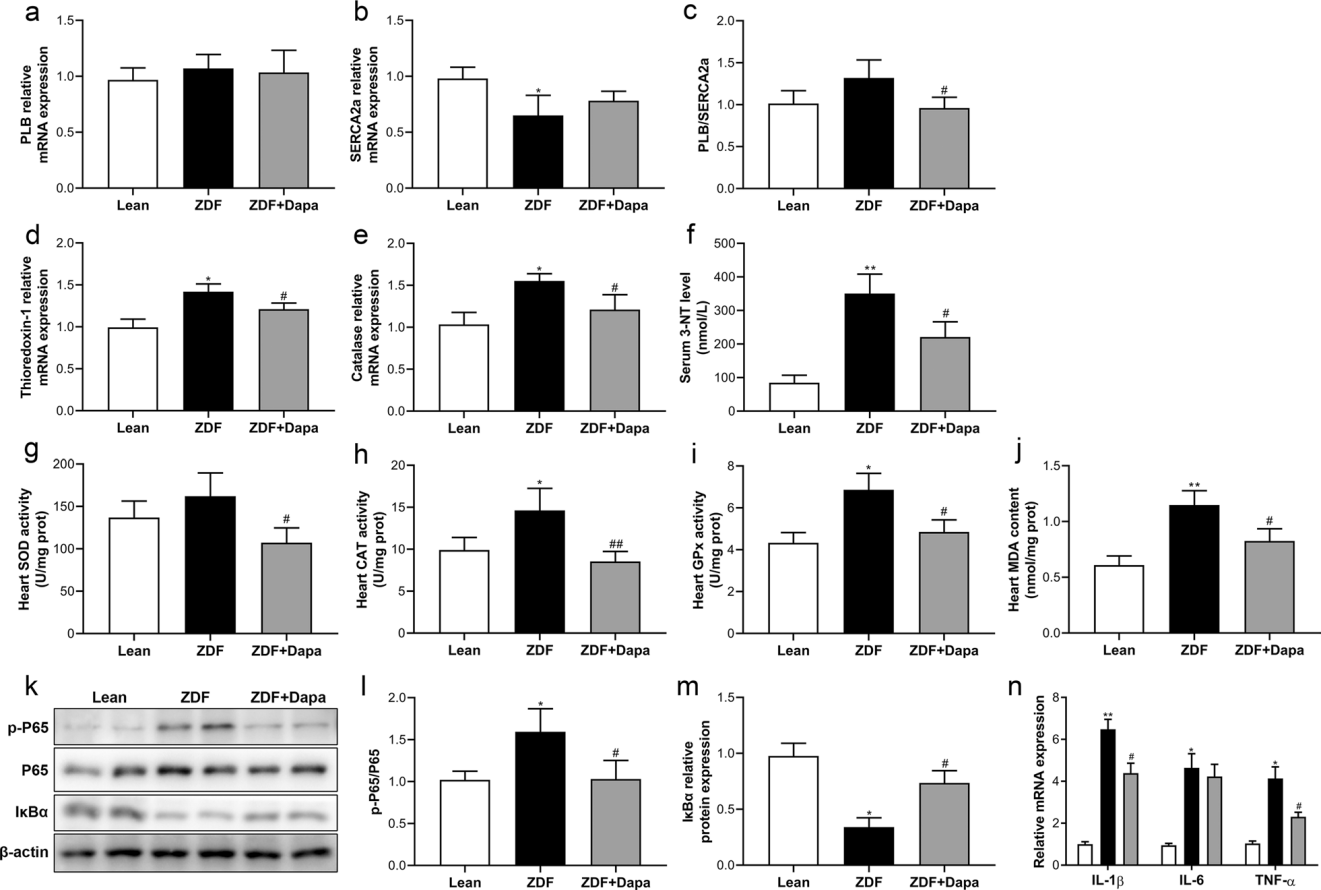
Dapagliflozin alleviated myocardial nitro-oxidative stress and inflammatory response. Real-time PCR measurements of **a**–**c** PLB and SERCA2a, **d** Thioredoxin-1, and **e** Catalase in the myocardium. **f** Serum 3-NT levels in the experimental groups. **g**–**i** The activity of classical antioxidants and **j** content of cardiac MDA. (k) Representative western blot bands of myocardial p-P65, P65, and IκBα levels. **l**, **m** Densitometric semi-quantitative determination of protein expression levels using ImageJ. **n** Gene expression of classic pro-inflammatory cytokines. Values are expressed as the mean ± SD. n = 8 per group. *P < 0.05, **P < 0.01 vs. Lean; ^#^*P* < 0.05 vs. ZDF

First, the gene expressions of myocardial SERCA2a and its inhibitory protein PLB were determined to assess calcium dysregulation in cardiomyocytes. The mRNA level of SERCA2a was significantly decreased in ZDF rats, as shown in Fig. 4a–c, but tended to rise after treatment. Although no difference in PLB gene expression was detected among groups, PLB/SERCA2a ratio was statistically reduced due to dapagliflozin treatment in ZDF animals. Next, we investigated the gene expression of antioxidant enzymes such as catalase and thioredoxin-1, and found that both were up-regulated in the ZDF group. Nevertheless, chronic drug treatment induced a significant decrease in catalase and thioredoxin-1 in the myocardial tissues of diabetic rats (Fig. 4d, e). In accord with this, we observed a marked elevation of serum 3-NT content in rats with diabetes, however, dapagliflozin prevention effectively reduced it (Fig. 4f). Further analysis showed that the activity of cardiac CAT and GPx, and content of MDA in heart tissues were increased in ZDF rats. Chronic drug treatment significantly mitigated abnormal changes in these markers (Fig. 3g–j).

Furthermore, western blot analysis demonstrated a remarkable increase in the ratio of phosphorylated p65 to total p65, and degradation of IκBα, suggesting myocardial NF-κB pathway activation in ZDF rats (Fig. 4k–m). However, dapagliflozin treatment for 12 weeks effectively prevented the abovementioned changes (Fig. 4k–m). The gene expression of classic pro-inflammatory factors TNF-α, IL-6, and IL-1β was further determined. Diabetes was associated with markedly elevated cytokines in the myocardium. Despite the unchanged mRNA level of IL-6, dapagliflozin substantially inhibited the gene expression of IL-1β and TNF-α in the ZDF group (Fig. 4n).

### Diabetes-associated cardiomyocyte apoptosis was inhibited by dapagliflozin

**Fig. 5:**
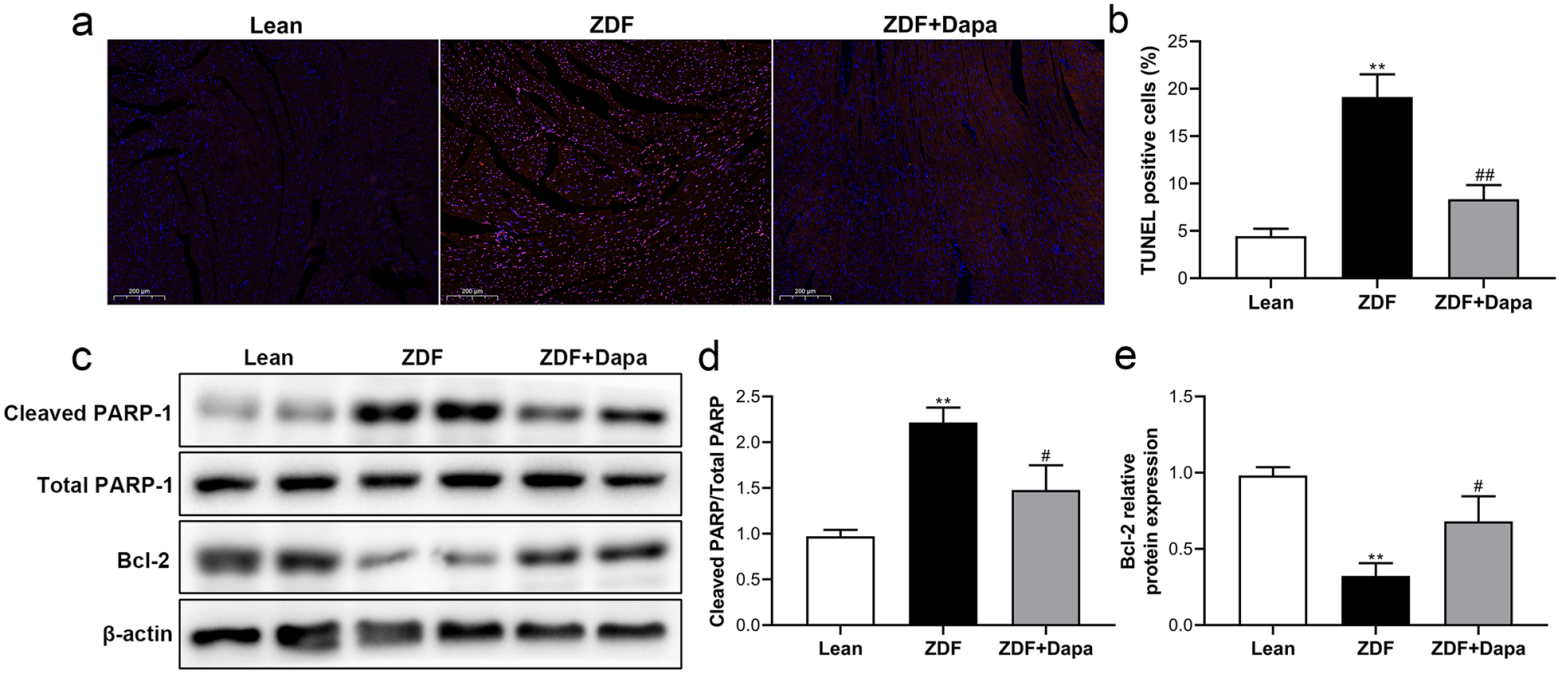
The effects of dapagliflozin on apoptosis and DNA fragmentation in cardiomyocytes. **a** Representative images of TUNEL staining. Bar = 200 μm, magnification = ×10. **b** Quantification of TUNEL-positive nuclei. **c** Representative western blot bands of cleaved PARP-1 and Bcl-2 levels in the myocardium. **d**, **e** Relative protein levels were densitometrically quantified with ImageJ. Data are expressed as the mean ± SD. *P < 0.05, **P < 0.01 vs. Lean; ^#^*P* < 0.05, ^##^*P* < 0.01 vs. ZDF

As presented in Fig. 5a–d, the percentage of TUNEL-positive cells and the protein content ratio of cleaved PARP/total PARP were both remarkably increased in the myocardium of diabetic rats. Meanwhile, the expression of anti-apoptotic protein B cell lymphoma 2 (Bcl-2) decreased in ZDF rats compared with lean controls (Fig. 5e). The dapagliflozin significantly prevented cardiomyocyte apoptosis in ZDF rats, which was evidenced by the reduced number of TUNEL-positive nuclei and cleaved PARP content, as well as up-regulated expression of Bcl-2 protein.

### Dapagliflozin activated the myocardial AMPK/mTOR pathway and restored autophagic flux in ZDF rats

**Fig. 6:**
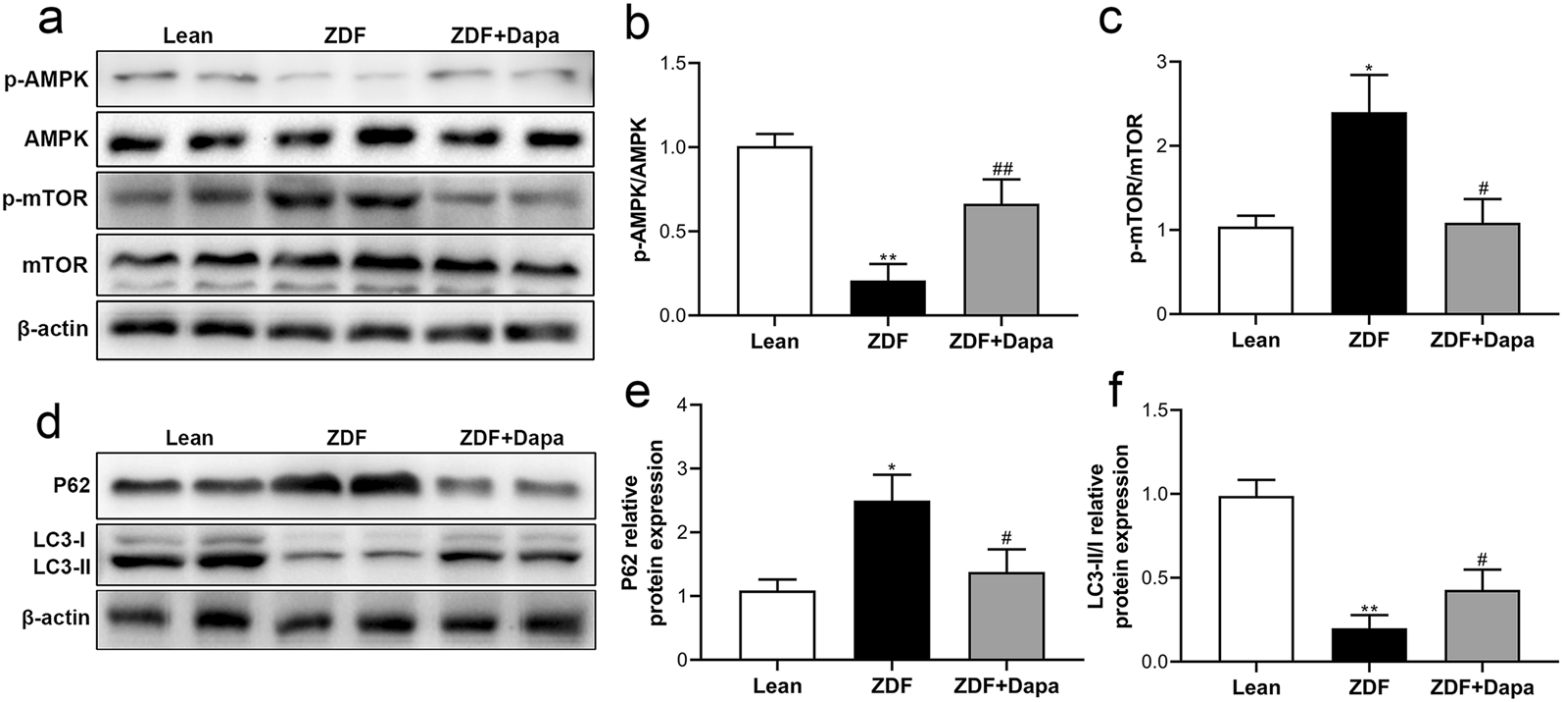
Modulatory effects of dapagliflozin on the myocardial AMPK/mTOR signaling pathway and autophagic flux in T2DM rats. **a** Representative western blot bands of p-AMPK and p-mTOR levels in the myocardium. **b**, **c** Densitometric semi-quantitative determination of protein expression. **d** Representative western blot bands of myocardial p62 and LC3-I/II expression. **e**, **f** Semi-quantitative analysis of relative protein levels. Data are expressed as the mean ± SD. *P < 0.05, **P < 0.01 vs. Lean; ^#^*P* < 0.05, ^##^*P* < 0.01 vs. ZDF

To investigate whether autophagy partly contributed to the cardioprotective effects of dapagliflozin, the autophagy process associated AMPK/mTOR signaling pathway was determined in cardiomyocytes of the experimental animals. As shown in Fig. 6a–c, western blotting revealed that dapagliflozin led to higher phosphorylation of AMPK and subsequent inactivation of mTOR in myocardial tissues of the treated rats as compared with the ZDF group. The content of LC3B and p62, two critical proteins involved in autophagic flux, was then determined in myocardial tissues. Dapagliflozin restored LC3B-II expression in ZDF rats and significantly elevated the ratio of LC3-II/I, accompanied by reduced p62 content, which is inversely correlated with cell autophagy. These data demonstrated that the AMPK/mTOR pathway, at least partly, mediated dapagliflozin-induced cardiomyocyte autophagy restoration in rats with T2DM.

### Demographic and clinical characteristics of study participants

A total of 105 subjects (52 males and 53 females) were enrolled in the study, including 30 normal controls (NC), 30 type 2 diabetes patients (DM), 25 DM patients with HFpEF (DM-HF), and 20 DM-HF patients treated with dapagliflozin (DAPA). Table 2 shows the anthropometric, clinical, and echocardiographic data on the participants. Combining HFpEF makes DM patients have higher FBG, HbA1c, NT-proBNP, and lower eGFR. However, when receiving chronic DAPA treatment, these abnormally elevated indicators were significantly alleviated. Furthermore, standard echocardiography was performed on all subjects. As shown in Table 2, there were no significant differences in the values of LVEDD, LVESD, IVSD, and LVPWT among the subjects in each group. HFpEF patients treated with DAPA had lower LVMI values than those in the DM-HF group. Importantly, although LVEF did not differ between groups, E/e' was lower in DAPA-treated diabetic patients with HFpEF, suggesting a significant improvement in diastolic function.

**Table 2:** Demographic and clinical data of all subjects

Variables	NC (n = 30)	DM (n = 30)	DM-HF (n = 25)	DAPA (n = 20)
Clinical characteristics				
Male (%)	50	53	40	55
Age (years)	59.2 ± 9.3	63.6 ± 8.1	69.7 ± 6.9	67.2 ± 12.8
BMI (kg/m^2^)	24.9 ± 2.8	24.9 ± 3.8	28.7 ± 4.1	25.5 ± 3.3^#^
SBP (mmHg)	132.3 ± 15.2	147.2 ± 19.9	151.9 ± 21.8	135.4 ± 16.1^#^
Heart rate (bpm)	72.0 ± 9.5	73.6 ± 9.5	75.5 ± 11.8	77.6 ± 12.5
FBG (mmol/L)	5.3 ± 0.7	7.4 ± 2.1	9.2 ± 2.3*	7.4 ± 2.1^#^
HbA1c (%)	5.8 ± 0.6	6.9 ± 1.2	8.6 ± 1.4*	7.5 ± 0.9^#^
Total cholesterol (mmol/L)	4.4 ± 1.2	4.5 ± 1.6	4.1 ± 1.0	3.9 ± 1.3
TG (mmol/L)	1.7 ± 1.1	1.9 ± 1.4	1.7 ± 1.1	1.7 ± 1.0
LDL-C (mmol/L)	2.6 ± 0.9	2.7 ± 0.9	2.2 ± 1.0	2.1 ± 0.8
HDL-C (mmol/L)	1.1 ± 0.3	0.9 ± 0.2	0.9 ± 0.3	0.9 ± 0.2
eGFR (mL/min/1.73 m^2^)	92.0 ± 11.5	90.2 ± 12.1	70.4 ± 22.5*	95.2 ± 25.6^#^
NT-proBNP (pg/mL)	92.6 ± 85.5	100.3 ± 74.6	1095.0 ± 910.4*	294.5 ± 391.9^#^
Echocardiographic parameters				
LVMI (g/m^2^)	92.6 ± 19.5	99.1 ± 27.0	115.4 ± 38.3	104.8 ± 27.9^#^
LVEDD (mm)	46.9 ± 5.2	48.4 ± 4.2	49.9 ± 7.8	48.8 ± 8.3
LVESD (mm)	30.4 ± 3.5	31.6 ± 4.2	33.9 ± 7.1	34.5 ± 9.9
IVSD (mm)	9.7 ± 1.7	10.1 ± 2.8	10.1 ± 2.3	11.0 ± 1.6
LVPWT (mm)	9.7 ± 0.7	9.5 ± 0.8	10.2 ± 1.4	9.8 ± 1.6
LA (mm)	34.3 ± 3.4	36.4 ± 4.5	40.4 ± 5.1*	39.2 ± 4.7
LVEF (%)	64.6 ± 5.5	63.4 ± 7.3	60.5 ± 9.4	60.6 ± 7.8
E/e'	9.2 ± 2.6	10.1 ± 2.8	12.8 ± 3.7*	10.7 ± 2.5^#^
E/A	0.9 ± 0.2	0.8 ± 0.3	0.8 ± 0.4	0.8 ± 0.3

Values are expressed as the mean ± SD or %. *P < 0.05 vs. DM; ^#^*P* < 0.05 vs. DM-HF. LVMI: left ventricular mass index, LVID: LVEDD, left ventricular end-diastolic diameter; LVESD: left ventricular end-systolic diameter; IVSD: inter ventricular septum at end-diastole; LVPWT: left ventricular posterior wall thickness; LA: left atrium; LVEF: left ventricular ejection fraction

### Effects of dapagliflozin on serum proteome in T2DM patients with HFpEF

**Fig. 7:**
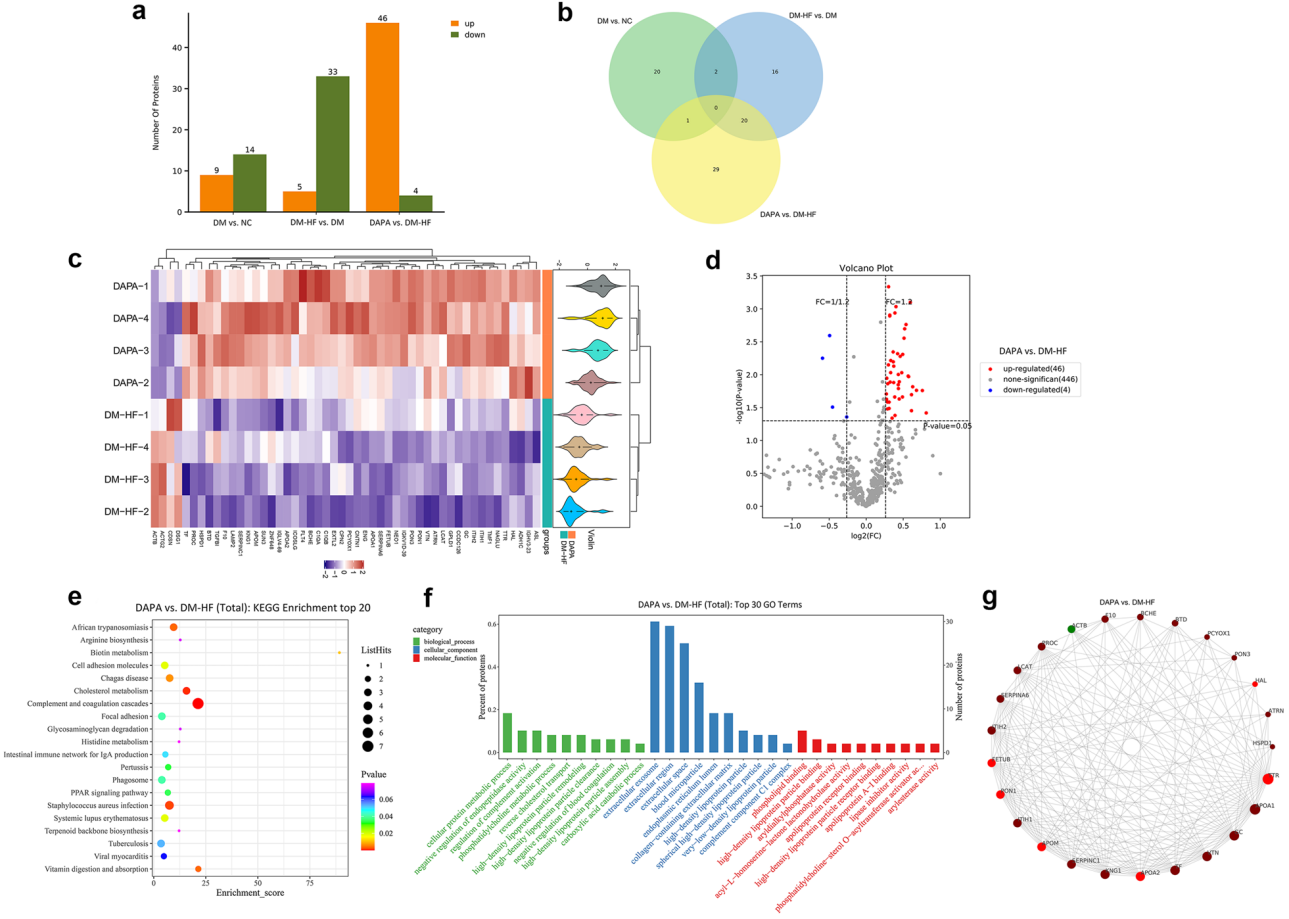
Proteomics deciphered the changes in the treated T2DM individuals with HFpEF. **a** Summary of differentially expressed proteins for each pairwise comparison. **b** Venn diagram showing the relationship between three pairwise comparisons. **c** Heatmap for protein detection from the proteomics data. Each column represents a protein marker, and each row represents a loading sample of the DM-HF and the DAPA group. **d** Volcano plots of the proteomics data for the pairwise comparison. Up-regulated and down-regulated proteins are marked with red and blue colors, respectively. **e** KEGG pathway enrichment and **f** GO term analyses. **g** The interaction relationship of differentially expressed proteins was obtained from the STRING database (https://string-db.org/), and the top 25 proteins were selected to draw the interaction network diagram. Red represents up-regulated genes, and the green represents down-regulated genes. The size of the circle represents the degree of connectivity

Furthermore, we examined changes in the protein contents in healthy or diabetic individuals using serum proteomic analyses. In total, 553 differentially expressed proteins were quantified across all 16 loading samples (4 groups). After pre-processing and missing value filtering, the expression profile of 496 proteins was used for further analysis (Additional file 7: Table S2). Differential expression analysis was performed on pairwise two-sample t-tests to define proteins altered in different groups of individuals ranging from NC to DAPA. Between two groups (DM vs. NC, DM-HF vs. DM, and DAPA vs. DM-HF, respectively), 23, 38, and 50 significantly differentially expressed proteins were identified (Fig. 7a). Venn diagram analysis shows an overlap of differentially expressed proteins after all the pairwise comparisons (Fig. 7b). The heatmap shows 46 up-regulated and 4 down-regulated proteins when compared DAPA group with DM-HF group (Fig. 7c). We also marked differentially expressed proteins on the volcano plots of pairwise comparisons within DAPA and DM-HF groups (Fig. 7d). Furthermore, these proteins were pooled for KEGG pathway enrichment and GO term (biological process, cell component, and molecular function) analyses (Fig. 7e, f). The proteins expressed differentially in the DAPA group were involved in multiple processes, including cholesterol metabolism, phosphatidylcholine, high-density lipoprotein particle metabolic process, and peroxisome proliferator-activated receptor (PPAR) signaling. Each protein involved was defined as a node, and the top 25 nodes in terms of connectivity were visualized and displayed with gene names, respectively (Fig. 7g). The heatmaps, volcano plots, and results of KEGG pathway enrichment and GO term analyses of pairwise comparisons within NC, DM, and DM-HF groups are presented in Additional file 2: Fig. S1 and Additional file 3: Fig. S2.

### Serum metabolome regulation in T2DM patients with HFpEF after dapagliflozin treatment

**Fig. 8:**
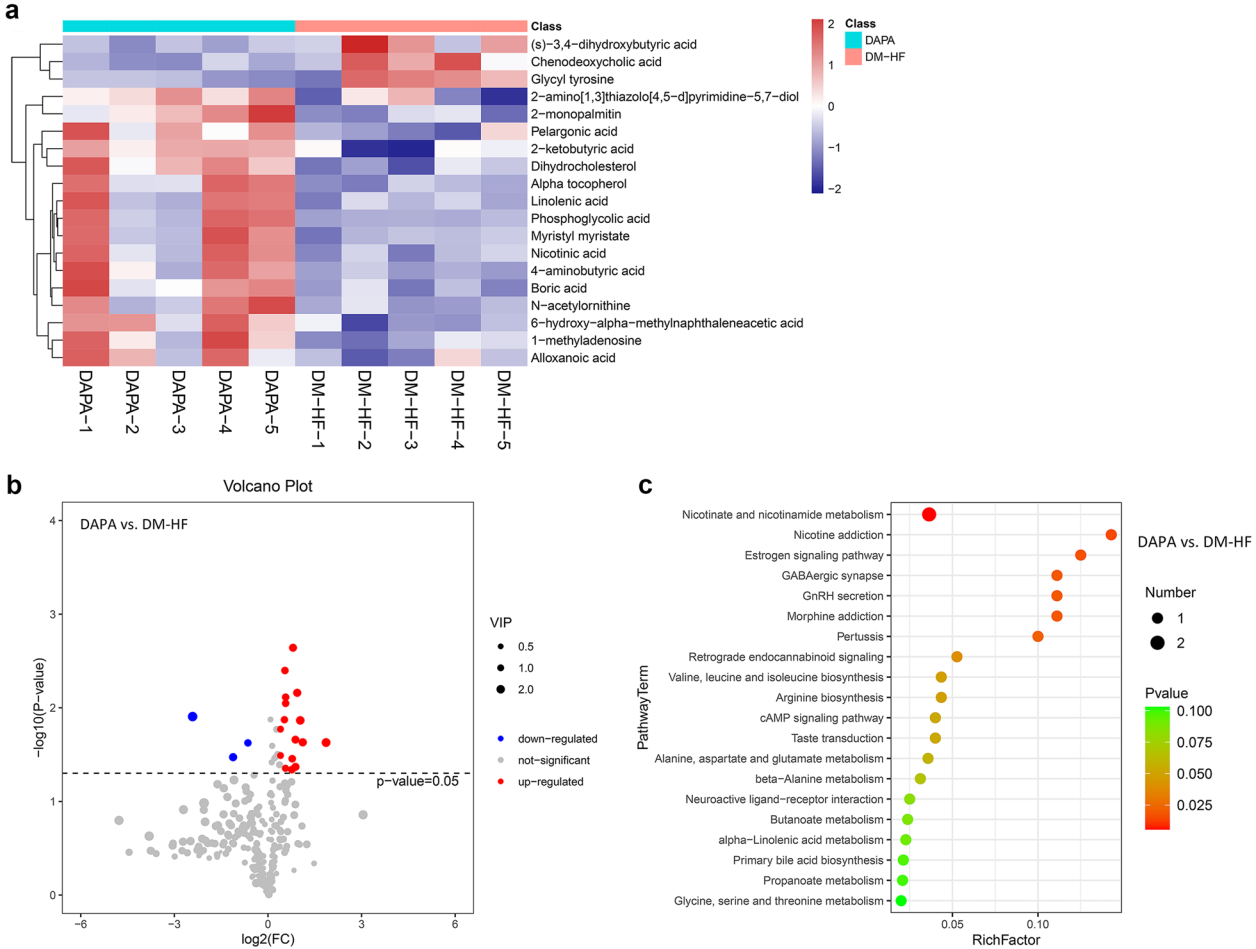
Metabolomics revealed the effects of dapagliflozin on serum metabolite profiles. **a** Heatmap for differentially expressed metabolites. Each row represents a metabolite marker, and each column represents a loading sample. **b** Volcano plots of the metabolomics data for the pairwise comparison. Up-regulated and down-regulated proteins are marked with red and blue colors, respectively. **c** Top 20 significant functional pathways enriched by differentially expressed metabolites

In order to reveal the changes in serum metabolite profiles more directly and accurately in patients with T2DM complicated with cardiac dysfunction after dapagliflozin treatment, we performed metabolomics analysis. After removing exogenous metabolites identified by Lumingbio's untargeted GC–MS database, 300 metabolites were analyzed further (Additional file 8: Table S3). Pairwise two-sample t-tests were performed on each metabolite, and 77, 43, and 19 differentially-abundant metabolites were identified between two groups (DM vs. NC, DM-HF vs. DM, and DAPA vs. DM-HF, respectively). As shown in the heatmap (Fig. 8a), the top up- regulated and down-regulated metabolites in the comparison between DAPA and DM-HF group identified 2-ketobutyric acid, 4-aminobutyric acid, *N*-acetylornithine, nicotinic acid, and Linolenic acid. Marked differentially abundant metabolites were also visualized on the volcano plots (Fig. 8b). The biological significance of these metabolites was then determined using KEGG pathway analyses. Finally, bubble plots were generated to illustrate the top significant pathways enriched by these metabolites. For DAPA versus DM-HF, nicotinate and nicotinamide metabolism, valine, leucine, isoleucine, and arginine biosynthesis, and cAMP and estrogen signaling pathway were markedly changed (Fig. 8c). The heatmaps, volcano plots, and results of KEGG pathway enrichment of pairwise comparisons within NC, DM, and DM-HF groups are shown in Additional file 4: Fig. S3 and Additional file 5: Fig. S4.

## Discussion

This study addressed the following issues regarding the therapeutic effect of dapagliflozin on T2DM-associated HFpEF: dapagliflozin (i) effectively prevents concentric hypertrophy, myocardial stiffness, and diastolic dysfunction in diabetic rat models, (ii) significantly mitigates the extent of cardiac nitro-oxidative stress, inflammation, and cardiomyocytes apoptosis, and (iii) enhances myocardial autophagic flux, possibly through an AMPK/mTOR-signaling pathway-dependent mechanism. However, the remarkable cardiovascular benefits of SGLT2is could not be attributed solely to their glucose and blood pressure-lowering or diuretic effect properties. Furthermore, the existing clinical and basic research evidence did not accurately tell us the detailed mechanisms of SGLT2is to improve diabetic cardiomyopathy.

HFpEF patients are generally older and predominantly female, with multiple comorbidities such as hypertension, diabetes, obesity, coronary artery disease, atrial fibrillation, and sleep apnea [30]. Approximately one-third of HFpEF patients have T2DM, and cardiovascular disease is the leading cause of morbidity and mortality for diabetic patients [31]. Although our knowledge of HFpEF has dramatically improved, diabetes-related cardiac dysfunction remains insufficiently understood. For this reason, mechanism-specific therapy for diabetic cardiomyopathy is currently not available.

The diabetic heart is generally associated with left ventricular diastolic dysfunction, cardiomyocyte hypertrophy, myocardial interstitial fibrosis, oxidative stress, and apoptosis dysregulation [4, 32]. These characteristics are well reproduced in the ZDF rats in our study. Meanwhile, we observed a significant increase in left ventricular stiffness, prolonged relaxation time, and preserved ejection fraction in experimental rats, which fully meet the definition criteria of HFpEF. Furthermore, previous findings have well proved that excessive production and deposition of extracellular matrix, principally collagen I and III, can alter the heart structure and lead to severe cardiac dysfunction [24, 33]. In the present study, we detected a significant decrease in collagen content in the heart of ZDF rats treated with dapagliflozin. Moreover, 12 weeks of drug intervention also alleviated diabetes-induced high expression of TGF-β. This result was supported by recent research demonstrating that the stimulation of TGF-β can directly activate the process of myocardial fibrosis and contribute to diastolic dysfunction [8]. Therefore, dapagliflozin will likely play a beneficial role in regulating pathological ventricular remodeling by hindering fibrosis progression.

Mechanistically, the diabetic heart is in a state of "metabolic rigidity" due to progressive obesity, hyperlipemia, hyperglycemia, and excessive utilization of fatty acids, which subsequently lead to oxidative stress and inflammation burden in the cardiomyocytes, and finally promote cardiac remodeling and diastolic stiffness [5, 6, 34, 35]. Here, we reported that dapagliflozin effectively prevented nitro-oxidative stress in myocardium. Interestingly, the expression or activity of cardiac antioxidant enzymes in ZDF rats was significantly increased. We hypothesize that this may be an adaptive and compensatory autoregulatory mechanism in response to obesity and hyperglycemia-induced stress stimuli. After the intervention of dapagliflozin, various adverse factors were alleviated, and then the reactivity of the antioxidant system was weakened. Moreover, considering the pivotal role of the NF-κB pathway in the pathogenesis of metabolic cardiomyopathy has been adequately validated [36, 37], it may be the most critical target that mediates dapagliflozin's anti-inflammatory effects in diabetes. In addition, PLB/SERCA2a ratio was increased in the myocardial tissue of ZDF rats, suggesting a disturbance of intracellular Ca^2+^ homeostasis in the context of diabetes. Interestingly, this ratio decreased in the ZDF + Dapa group, indicating that dapagliflozin may improve left ventricular stiffness and prolong relaxation time via regulating intracellular Ca^2+^, not just through conventional mechanisms.

Although most studies concluded that a high-fat diet inhibits autophagy [38, 39], a clear consensus as to whether autophagy is protective or detrimental in the diabetic heart has not been accomplished. Our data showed that myocardial autophagy was strongly suppressed in ZDF rats, as evidenced by a decreased LC3 II/I ratio and increased p62 protein expression. Sadoshima et al. proposed that the activation of autophagy could protect the heart during diabetic cardiomyopathy by counteracting the excessive accumulation of dysfunctional cellular waste [40]. Beclin1 is a Bcl-2 homology 3 (BH3) domain-only protein that plays a vital role in autophagy initiation. AMPK activation causes the interruption of the association between Beclin1 and Bcl-2, allowing free Beclin1 to combine with class III PI3K to form a kinase complex, which is essential for inducing autophagy [41]. Moreover, liraglutide-induced enhancement of autophagy was related to increased AMPK phosphorylation but decreased mTOR phosphorylation in ZDF rats [42]. In line with this, the present findings suggest that the AMPK-mTOR signaling pathway is involved in dapagliflozin-mediated autophagy restoration in cardiomyocytes. Regrettably, we did not dig deep into the upstream mechanism underlying AMPK activation with dapagliflozin in a hyperglycemic state. Besides aberrant autophagy, the interplay between autophagy and apoptosis is also critical in the pathogenesis of cardiomyopathy [38, 43]. Herein, the increased apoptosis of cardiomyocytes in ZDF rats was weakened with dapagliflozin treatment. These findings are consistent with a previous study, demonstrating that diabetic mice exhibited suppressed autophagy and augmented apoptosis in myocardial tissues [13]. Therefore, the modulation of abnormal autophagy and apoptosis of cardiomyocytes is likely to be the internal mechanism of dapagliflozin to protect heart function in T2DM patients.

Pharmacologic therapy for HFpEF represents one of the greatest unmet medical needs. Generally, the current opinion believes that the pathogenesis of diabetes-associated HFpEF is multifaceted. Thus, the treatment strategy could be to find a "one-size-fits-all" approach. More and more animal and clinical studies have told us that SGLT2is can improve diastolic function in subjects with or without diabetes, and its underlying mechanisms are widely discussed [21, 44–46]. Dapagliflozin has been shown to reverse left ventricular concentric remodeling in HFpEF pigs by restraining sympathetic tone in the aorta and inhibiting NO-cGMP-PKG pathway in our previous work [26]. Recently, Winzer et al. reported similar cardioprotective effects of SGLT2i in a diabetes-associated HFpEF model, where ZSF1-obese rats were treated with empagliflozin for 8 weeks [47]. Increasing scientific evidence proposes that SGLT2is may have direct effects on the heart, including a reduction in diastolic myofilament stiffness, up-regulation of the global phosphorylation of myofilament protein, and inhibition of cardiac late sodium channel current activity [48, 49]. Multi-omics study is an emerging method in recent years to explore the interactions between multiple substances in biological systems. In the present study, we performed proteomic and metabolomic assays on serum samples from diabetic patients with HFpEF. Using bioinformatics analysis, we have made some interesting findings. Compared with the standard hypoglycemic drug group, the differentially expressed proteins in the serum of HFpEF patients after dapagliflozin treatment were mainly related to the process of cholesterol metabolism, especially the peroxisome proliferator-activated receptor (PPAR) signaling pathway. In terms of metabolite phenotype, serum metabolome analysis suggestted that metabolites related to nicotinamide metabolism, arginine biosynthesis, and cAMP and estrogen signaling pathways were significantly changed in the serum of diabetic patients under long-term dapagliflozin treatment. These findings provide valuable molecular biological clues for exploring the mechanism of SGLT2 inhibitors improving myocardial injury independent of their hypoglycemic effect. For instance, nicotinamide is a key component of the coenzymes nicotinamide adenine dinucleotide (NAD) and nicotinamide adenine dinucleotide phosphate (NADP), both of which are involved in the respiratory chain oxidation in mitochondria and energy generation. Therefore, it is reasonable to speculate that dapagliflozin may have a protective effect on the occurrence of diabetic cardiomyopathy by improving mitochondrial function and energy metabolism of cardiomyocytes.

In the light of very recent press releases, the EMPEROR-Preserved trial and the DELIVER trial showed that empagliflozin and dapagliflozin reduced the primary outcome of heart failure hospitalizations and cardiovascular death in HFpEF patients [22, 50]. In the meanwhile, we remain optimistic about the ongoing DETERMINE-preserved trial and believe that SGLT2is will open up new avenues for treating diabetes-related heart failures.

## Conclusion

Our results illustrate that dapagliflozin might protect against cardiac concentric hypertrophy and diastolic dysfunction in diabetic rats by mitigating myocardial fibrosis, nitro-oxidative stress, and inflammation and repairing the balance between apoptosis and autophagy. These findings will help explain the molecular mechanism underlying this drug-mediated improvement in heart function in T2DM patients and help develop diabetic cardiomyopathy medication.

## Supplementary Information

**Additional file 1. **Data supplement.

**Additional file 2: Figure S1.** Differentially expressed proteins and pathway analysis of comparing DM and NC.

**Additional file 3: Figure S2.** Differentially expressed proteins and pathway analysis of comparing DM-HF and DM.

**Additional file 4: Figure S3.** Differentially expressed metabolites and pathway analysis of comparing DM and NC.

**Additional file 5: Figure S4.** Differentially expressed metabolites and pathway analysis of comparing DM-HF and DM.

**Additional file 6: Table S1.** The sequences of rat primers for RT-PCR analysis.

**Additional file 7: Table S2.** Proteomics data for protein expression across 16 loading samples (4 groups).

**Additional file 8: Table S3.** Metabolomics data for serum metabolite profiles across loading samples (1 QC group and 4 experimental groups).

## Data Availability

The datasets generated and analyzed for this study are available from the corresponding author upon reasonable request. The mass spectrometry proteomics data have been deposited to the ProteomeXchange Consortium (http://proteomecentral.proteomexchange.org) via the iProX partner repository with the dataset identifier PXD037538.

## Abbreviations

HFpEF: Heart failure with preserved ejection fraction
HFrEF: Heart failure with reduced ejection fraction
SGLT2i: Sodium glucose cotransporter 2 inhibitor
PPAR: Peroxisome proliferator-activated receptor
LV: Left ventricular
NT-proBNP: N-terminal pro-b-type natriuretic peptide
HW: Heart weight
TL: Tibia length
ANP: Atrial natriuretic peptide
AW: Anterior wall thickness
PW: Posterior wall thickness
LVID: LV internal diameter
RWT: Relative wall thickness
IVSd: Inter ventricular septum at end-diastole
DT: Deceleration time
IVRT: Iso-volumetric relaxation time
PRSW: Preload recruitable stroke work
GO: Gene Ontology
KEGG: Kyoto Encyclopedia of Genes and Genomes
