# Supplementary material for: Therapeutic effects on the development of heart failure with preserved ejection fraction by the sodium-glucose cotransporter 2 inhibitor dapagliflozin in type 2 diabetes
Source: Diabetol Metab Syndr. 2023 Jun 29;15:141. doi: 10.1186/s13098-023-01116-8 (PMC10308685; doi:10.1186/s13098-023-01116-8)
Supplement: Supplementary file 1 — Additional file 1. Data supplement. [file 13098_2023_1116_MOESM1_ESM.docx]

**Data Supplement**

**Detailed Methods**

**Protein Identification and Quantitation**

Sixteen labeled peptide mixtures were subjected to reversed-phase chromatographic separation and mass spectrometry analysis. The LC-MS/MS analysis was performed using a Q Exactive mass spectrometer (Thermo Fisher Scientific, USA). The MS full scan was acquired at a resolution of 70,000 with the scan range of 300–1800 m/z. The automatic gain control (AGC) target was set to 1e6, and the maximum inject time to 50 ms. MS data were acquired in 60 min by a data-dependent top10 method. All MS/MS spectra were acquired using collisional fragmentation in positive ion mode and the normalized collision energy was 32 eV. The MS/MS scans were acquired at a resolution of 35,000, and dynamic exclusion duration was 45 s. Database searching was conducted using Proteome Discover 2.4 (Thermo Fisher, USA) with parameters as follows: static modification: tandem mass tag (N-term, K), Carbamidomethyl (C); dynamic modification: oxidation (M), acetyl (N-term); digestion: trypsin; ms1 tolerance: 10 ppm; ms2 tolerance: 0.02 Da; missed cleavages: 2.

The raw data were imported in MaxQuant for analysis. The Human UniProt FASTA database (reviewed; 20,577 sequences) was used for protein identification. Database search was performed with Trypsin digestion specificity. Alkylation on cysteine was considered as fixed modifications in the database searching. A global false discovery rate (FDR) was set to 0.01 and protein groups considered for quantification required at least 2 peptides. For proteome quantitation, the output result was log2-transformed, and then data analysis such as missing value imputation, Hierarchical clustering, Principal Component Analysis (PCA), t-tests, and correlation were performed.

**Proteomics Data Analysis**

Proteomics data were collected across 16 loading samples with 4 loading samples for each of the four groups: NC, DM, DM-HF, and DAPA. After filtering out N/A values, a list of statistically-significant proteins was generated through a student’s t-test. For the pairwise comparisons across the four groups, a two-sample t-test was performed. Proteins with 95% confidence interval and with a fold-change cutoff value of 1.2 were considered significant (p < 0.05). Subsequently, differentially expressed (DE) proteins were pooled, and hierarchical clustering, correlations, and venn anslysis were performed.

**Nontargeted GC-MS Analysis**

The derivatized plasma samples were analyzed with a GC-MS system that consisted of a HP7890B gas chromatograph coupled to a quadrupole HP5977A mass spectrometric detector (Agilent Technologies Inc., CA, USA). The detector had a resolving power of unit mass resolution over mass range m/z 5-500 and mass accuracy of ± 0.2u. A DB-5MS fused-silica capillary column (30m×0.25mm×0.25μm, Agilent J & W Scientific, Folsom, CA, USA) was utilized to separate the derivatives. Helium (>99.999%) was used as the carrier gas at a constant flow rate of 1 mL/min through the column. The injector temperature was maintained at 260 °C. Injection volume was 1 μL by splitless mode. The column temperature was maintained at 60 °C for 0.5 min, ramped to 125 °C at a rate of 8 °C/min, to 210 °C at a rate of 5 °C/min，to 270 °C at a rate of 10 °C/min, to 305 °C at a rate of 20 °C/min, and finally held at 305 °C for 10 min. The collision energy was 70 eV. Mass spectra data were acquired after a solvent delay of 4 min in a full-scan mode (m/z 50-500). The ion source temperature and MS quadrupole temperature were 230 °C and 150 °C, respectively.

Raw data were acquired from Xcalibur 2.2 software (Thermo Scientific) in “.D” format and were converted to “abf” format via software with the Analysis Base File (ABF) Converter (<http://www.reifycs.com/AbfConverter/index.html>) for quick retrieval of data. Software MS-DIAL with the Fiehn library was used for raw peak exaction, data baseline filtering and calibration, peak alignment, MS2Dec deconvolution analysis, peak identification, and missing value interpolation [S1]. Metabolite characterization is based on LUG database. In each sample, all peak signal intensities were segmented and normalized according to the internal standards with RSD greater than 0.3 after screening. After the data was normalized, redundancy removal and peak merging were conducted to obtain the data matrix.

**Data Availability**

All MS raw data associated with the study, along with detailed tables of MaxQuant output files reporting m/z, charge, identification, quantitation, and other details for all peptide identifications, have been deposited to ProteomeXchange (Dataset PXD037538).

**References**

[S1] Tsugawa H, Cajka T, Kind T, et al. (2015) MS-DIAL: data-independent MS/MS deconvolution for comprehensive metabolome analysis. Nature methods 12: 523-6.
