# Supplementary figures and images for: Therapeutic effects on the development of heart failure with preserved ejection fraction by the sodium-glucose cotransporter 2 inhibitor dapagliflozin in type 2 diabetes
Source: Diabetol Metab Syndr. 2023 Jun 29;15:141. doi: 10.1186/s13098-023-01116-8 (PMC10308685; doi:10.1186/s13098-023-01116-8)

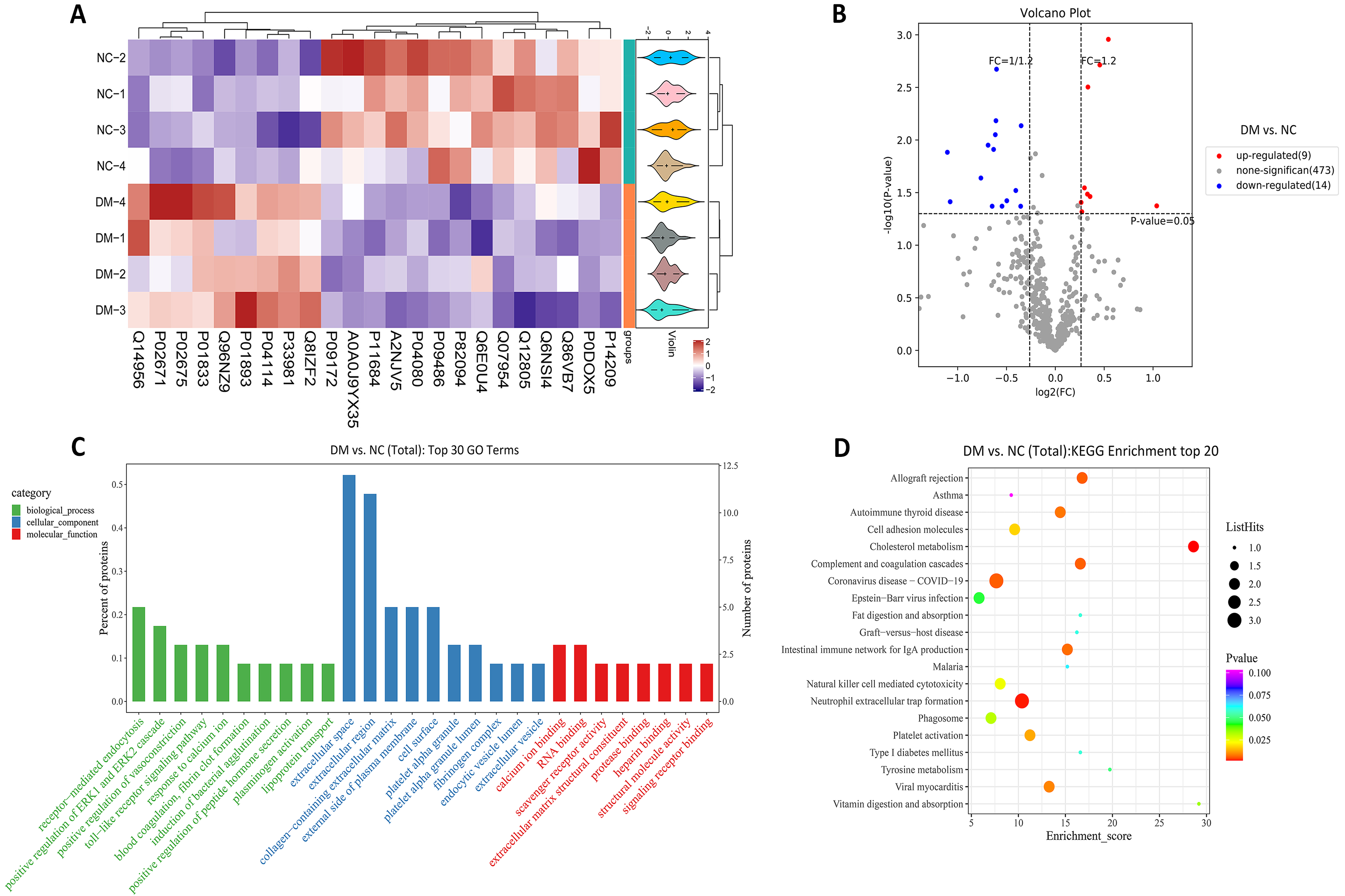

Supplement: Supplementary file 2 — Additional file 2: Figure S1. Differentially expressed proteins and pathway analysis of comparing DM and NC. [file 13098_2023_1116_MOESM2_ESM.tif]

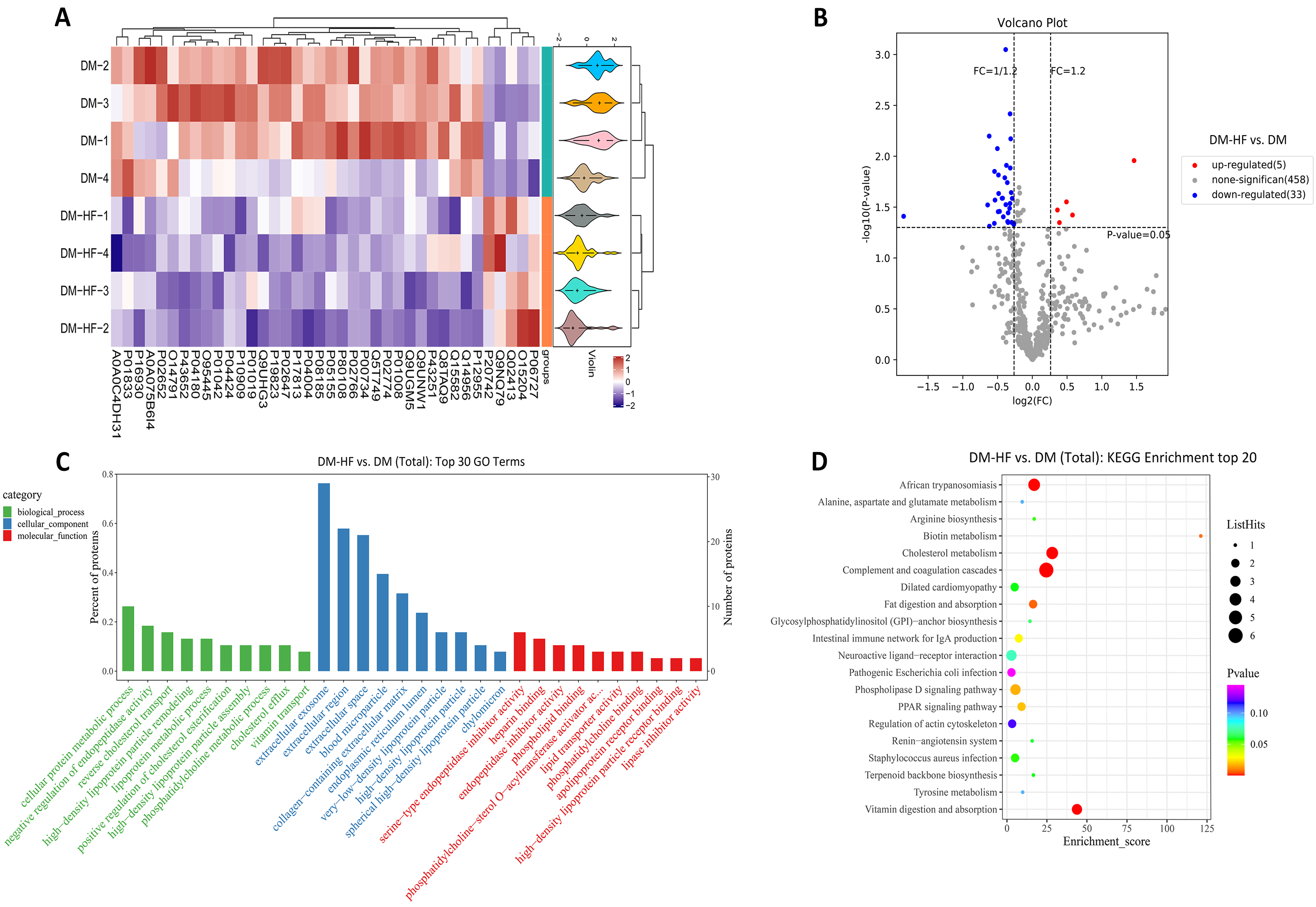

Supplement: Supplementary file 3 — Additional file 3: Figure S2. Differentially expressed proteins and pathway analysis of comparing DM-HF and DM. [file 13098_2023_1116_MOESM3_ESM.tif]

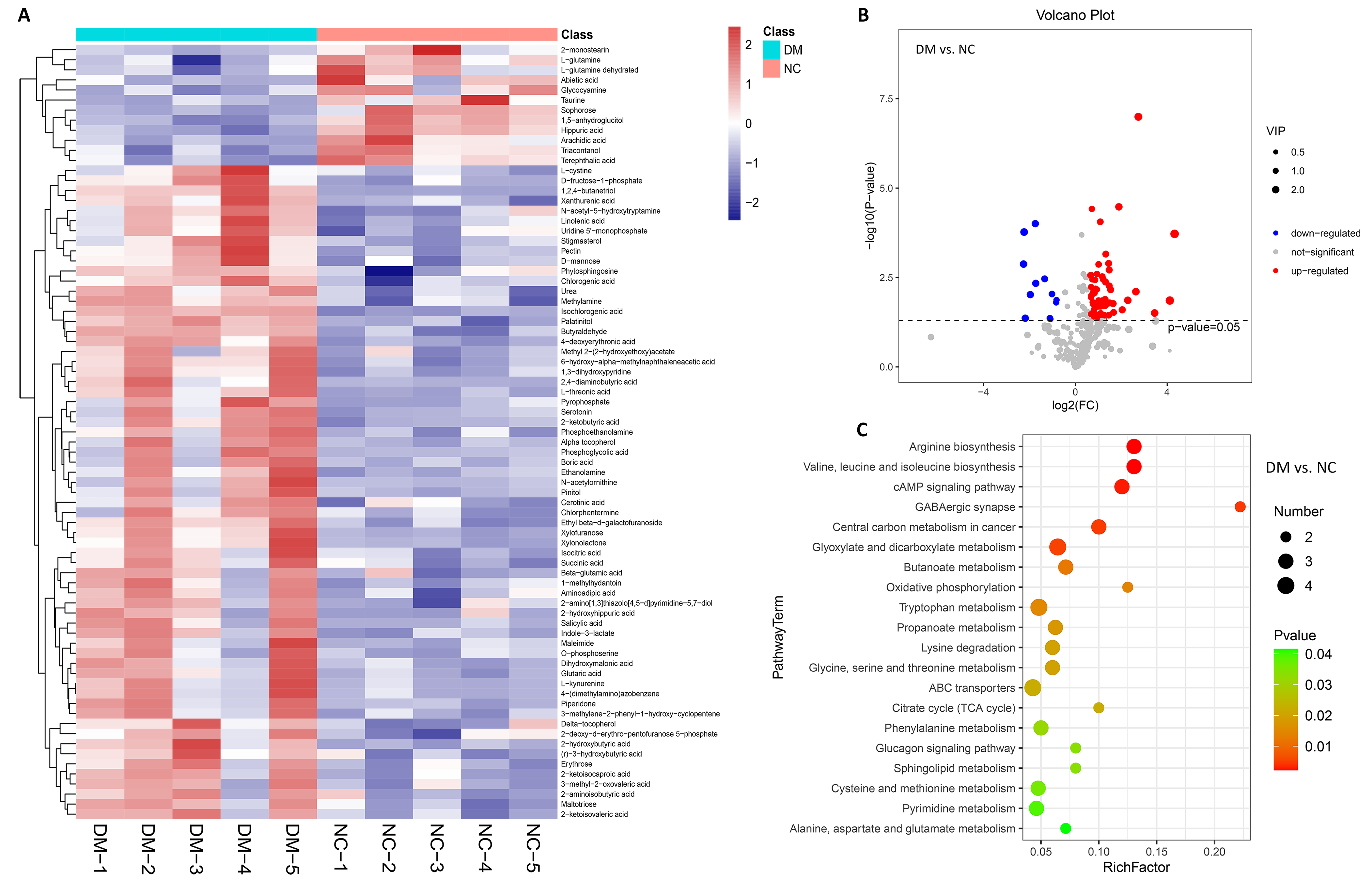

Supplement: Supplementary file 4 — Additional file 4: Figure S3. Differentially expressed metabolites and pathway analysis of comparing DM and NC. [file 13098_2023_1116_MOESM4_ESM.tif]

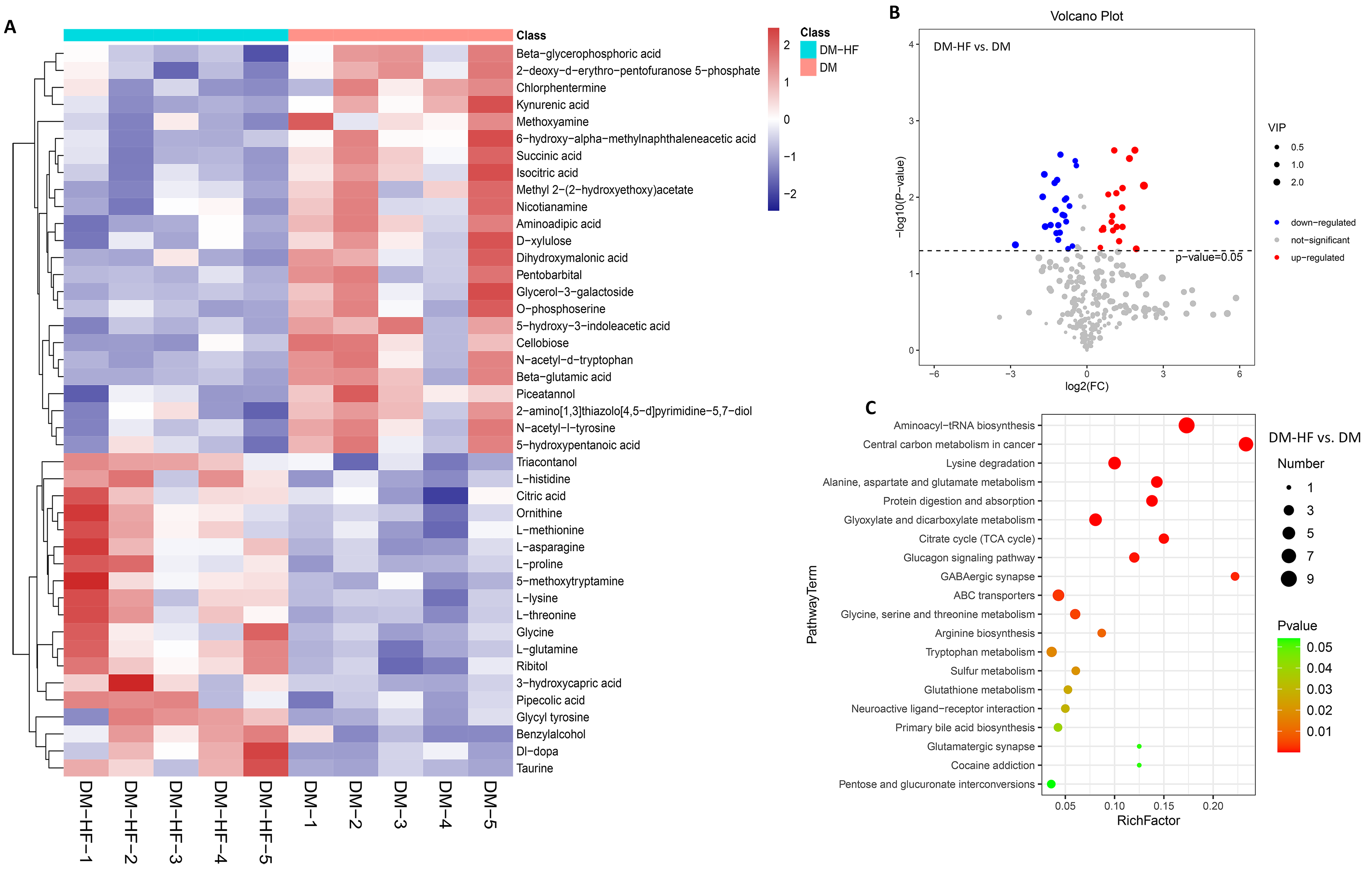

Supplement: Supplementary file 5 — Additional file 5: Figure S4. Differentially expressed metabolites and pathway analysis of comparing DM-HF and DM. [file 13098_2023_1116_MOESM5_ESM.tif]
