# Supplementary material for: Therapeutic effects on the development of heart failure with preserved ejection fraction by the sodium-glucose cotransporter 2 inhibitor dapagliflozin in type 2 diabetes
Source: Diabetol Metab Syndr. 2023 Jun 29;15:141. doi: 10.1186/s13098-023-01116-8 (PMC10308685; doi:10.1186/s13098-023-01116-8)
Supplement: Supplementary file 6 — Additional file 6: Table S1. The sequences of rat primers for RT-PCR analysis. [file 13098_2023_1116_MOESM6_ESM.pdf]

Table S1.

The sequences of rat primers for RT-PCR analysis

Gene name	Primer sequence (5’-3’)	Accession No.
β-actin	F: ATCGTGGGCCGCCCTAGGCACC	NM_031144.3
	R: CTCTTTAATGTCACGCACGATTTC	
Fibronectin-1	F: ATGTGGACCCCTCCTGATAGT	NM_019143.2
	R: GCCCAGTGATTTCAGCAAAGG	
Collagen-1	F: GAGCGGAGAG TACTGGATCGA	NM_053304.1
	R: CTGACCTGTCTCCAT- GTTGCA	
TGF-β	F: CTCCCGTGGCTTCTAGTGC	NM_021578.2
	R: GCCTTAGTTTGGACAGGATCTG	
Thioredoxin-1	F: CATGCCGACCTTCCAGTTTTA	NM_053800.3
	R: TTTCCTTGTTAGCACCGGAGA	
Catalase	F: AGCGACCAGATGAAGCAGTG	NM_012520.2
	R: TCCGCTCTCTGTCAAAGTGTG	
IL-1β	F: CACCTCTCAAGCAGAGCACAG	NM_031512.2
	R: GGGTTCCATGGTGAAGTCAAC	
IL-6	F: GAGTTGTGCAATGGCAATTC	NM_012589.2
	R: ACTCCAGAAGACCAGAGCAG	
TNF-α	F: TACTCCCAGGTTCTCTTCAAGG	NM_012675.3
	R: GGAGGCTGACTTTCTCCTGGTA	
PLB	F: AAAGTGCAATACCTCACTCGC	NM_023129.5
	R: GGCATTTCAATAGTGGAGGCTC	
SERCA2a	F: GAGAACGCTCACACAAAGACC	NM_009722.3
	R: CAATTCGTTGGAGCCCCAT	

Accession numbers are from the GenBank database.
