# Supplementary material for: Therapeutic effects on the development of heart failure with preserved ejection fraction by the sodium-glucose cotransporter 2 inhibitor dapagliflozin in type 2 diabetes
Source: Diabetol Metab Syndr. 2023 Jun 29;15:141. doi: 10.1186/s13098-023-01116-8 (PMC10308685; doi:10.1186/s13098-023-01116-8)
Supplement: Supplementary file 7 — Additional file 7: Table S2. Proteomics data for protein expression across 16 loading samples (4 groups). [file 13098_2023_1116_MOESM7_ESM.pdf]

Table S2. Proteomics data for protein expression across 16 loading samples (4 groups).

Accession	Protein	DAPA-1	DAPA-2	DAPA-3	DAPA-4	NC-1	NC-2	NC-3	NC-4	DM-1	DM-2	DM-3	DM-4	DM-HF-1	DM-HF-2	DM-HF-3	DM-HF-4
P04114	APOB	114	130.1	78.5	71.3	87.1	72.9	68.9	85.8	105.1	112.8	125	115.5	139	75	95.4	123.7
P01024	C3	98.4	107.6	95	118.2	106.8	108.2	112.9	108.1	113.8	91.7	97.4	87.9	86.5	81.2	90.5	95.9
P01023	A2M	115.5	85.1	93.6	111.9	109	162.2	103.9	86.6	89.9	107.6	74.1	100.2	86.3	63.2	123	87.7
P0C0L5	C4B	110	112.9	97.3	109.4	119.7	79.5	81.8	78.3	84.5	100.5	94	104.8	110.6	86.9	115.3	114.4
P0C0L4	C4A	105.7	95.1	92	106.5	110.1	93.2	86.4	82.5	112.6	91.6	94.5	105	124.5	100.1	105.4	94.8
P02787	TF	94	105.6	97.4	111.9	93.2	125.2	101	107.4	132.5	110	110.5	76.7	92.3	85.6	68.1	88.8
P02751	FN1	118.3	116.7	117.6	109.1	100.1	65.4	65.2	86.5	91.6	125.1	80.2	114.9	67.6	102.4	66.6	172.7
P00450	CP	94.6	100	93.3	113.4	99.1	107.8	136.3	85.2	102.4	95.2	100.8	90.2	99.2	86.8	99.3	96.4
P02768	ALB	70.6	65	100.9	80	70.9	71.9	100.1	129.6	88.6	74.1	106.3	105.9	152.6	249.7	83.4	50.3
P02675	FGB	84.3	111	108.4	92.7	85.4	78.5	79.9	73.7	92.7	84.4	92.7	113.4	75.6	139.9	112.2	175.3
P02671	FGA	83.2	114.4	108.3	91.8	82.6	73.9	76.1	71.3	90.1	84.6	92.3	114.6	73.7	143.4	114.6	185.1
P01009	SERPINA1	98.7	85.2	109.6	108.6	87.5	114.3	116.5	94.3	114.9	94.5	96.5	104.3	87.8	99.3	104.9	83.1
P08603	CFH	91.8	90	83.7	140.9	106.9	90.5	99.8	116.8	101.8	74.5	126.6	108.6	110	103.9	88.2	66.1
P01031	C5	92.6	99.1	97.3	91.1	104.8	72.5	95.4	127.5	110.7	83.6	104.3	97.1	126.7	112.9	106.8	77.3
P02774	GC	106.3	97	109.4	96.2	87.5	106.1	124.8	113.5	122.8	111	111.5	86.2	84.9	79.2	80.4	83.4
P00734	F2	96.1	102	105.1	104.7	100.1	93.9	103.6	110.9	123.2	101.9	112.3	90.3	87.1	88.5	88	92.4
P02647	APOA1	103.2	92	105	102.3	129.1	110	84.9	143	94.4	123.9	115	84.4	89.9	72.1	72.4	78.3
P00738	HP	100	125	94.1	27.6	124.3	58.2	112.3	79.5	56.7	102.5	62.5	101.9	99.4	111.6	211.3	133.1
Q14624	ITIH4	99.8	98.6	99.9	113.2	110.7	93.2	97.2	108.3	107.6	98.7	95.4	90	92.1	107.1	97.8	90.6
P19827	ITIH1	114	103.4	109.9	106.6	115.9	94.5	110.9	75.3	104.5	112.3	118.9	82	91.5	82.5	89.1	88.8
P02679	FGG	85.2	113.4	110.2	95.4	83.1	80.7	80.2	71.3	89.9	84.9	89.9	110.7	74.8	140.6	113.9	176
P01008	SERPINC1	101.7	103.7	115.6	117.6	92.2	106.8	110.3	96.5	120.6	111.6	102.6	85.6	84.1	78.6	85.2	87.3
P00751	CFB	95.8	100.8	99.7	87	81.4	94.9	120.5	95.7	127.5	96.6	124.1	107	88.2	109.5	82.7	88.6
P02790	HPX	96.4	105.7	96.8	105.6	117	101.6	105.9	93.8	108.6	106.6	104.2	93.5	85	91.1	93.9	94.4
P01042	KNG1	99.4	105.4	112.5	122.9	104.4	98.3	72.1	100.7	108.7	106.9	119.3	99.4	86.4	86.7	80.8	96.2
P19823	ITIH2	112.9	97.9	109	109.1	115.7	102.1	101.8	104.3	101	122.1	114.7	84.9	89.3	76.2	78.7	80.3
P04275	VWF	85.2	124.9	115.1	93.2	90.9	62.6	62.1	74.6	68.9	111.8	83	130.8	62.1	115.4	124.7	194.7
P13645	KRT10	87.1	88.1	90.9	75.5	147.7	127.7	101.2	77.6	95.8	132.7	114.5	68.2	123	87.6	102.7	79.8
P06727	APOA4	124	82.5	147.2	96.4	107.8	82.3	72.4	79.7	98.2	93.3	87	71.1	98.4	140.2	108.3	111.3
P43652	AFM	131.5	102.1	97.1	87.6	109.4	104.9	93.6	134.3	110.6	109.7	123.6	87.4	91.2	66	64.6	86.4
P06396	GSN	117.5	67.9	113.1	106.6	108.1	118.9	93.6	101	115.1	100.3	87.4	91.1	95.8	127.1	71.9	84.7
P10643	C7	84.1	84	112.9	89.8	85.5	88.8	89.5	114.4	115	64.3	100.2	108.9	124.7	133.3	121.7	82.9
P01871	IGHM	129.7	124.4	83.5	74.2	79.8	104.1	124.7	103.3	73.8	168.2	78.7	87.6	159.4	69.3	76.9	62.3
P01011	SERPINA3	90.7	99.9	89.9	109.8	85.1	93.4	149.4	72.3	87.9	95.4	101.1	88.8	91.9	103.8	145.6	94.9
P09871	C1S	109.2	90.4	99.7	102.3	76.7	99.6	91.3	101.9	102.8	105.9	111.4	112.9	101.3	93.9	96.1	104.6
P13671	C6	104.7	107.1	97.5	99.3	93.7	86.9	96.2	109.2	118.6	88.1	100.6	96.6	100.7	95.4	104.6	100.7
P20742	PZP	88.6	144.9	51.7	44.2	182.8	37.4	179.4	113.5	42.3	60.9	55.4	42.9	175	78.6	122.3	180.2
P04264	KRT1	88.6	92.6	97.6	76.1	123.1	118.6	115.8	83	94.2	128.9	106	80.4	131.5	77.8	101.7	84
P00739	HPR	102.5	115.6	115.7	89.3	104.2	79.4	146.8	94.6	54.7	86	128	139.7	78.5	100.5	71.3	93.3
P00747	PLG	80.6	91.9	100.6	90.4	106.5	65.8	79.6	159.3	114.8	78.5	111.9	115.8	125.4	121.7	85.7	71.4
P01876	IGHA1	97	76.4	99.9	142.7	115.4	134.9	126.2	88.8	90	83.6	79.4	120.6	141.7	54.3	87.4	61.4
P12259	F5	101.8	90.1	110.6	93	122.5	98	93.7	117.2	104.5	95.6	99.2	115.9	93.7	92.9	85.6	85.8
Q06033	ITIH3	103.1	121.6	110.7	104.9	110.4	81.6	139.4	72.6	72.2	74.6	87.2	80.8	99.8	120.3	139.5	81.3
P04003	C4BPA	81.7	100.6	97.3	85	169.8	72.3	83.4	122.2	108.4	85.4	102	98.7	101.9	102.3	104.5	84.4
P01860	IGHG3	129.1	64.2	72.7	57.6	58.6	142.4	103.9	124.2	43.6	62	65.3	237	126.1	91	54.8	167.5
P35527	KRT9	91.9	103.5	98.4	80.8	108.9	119.2	121.8	87.8	88.7	123.8	94.6	91.2	118	73.7	102.7	94.9
P08697	SERPINF2	107.7	112.1	93.3	108.9	110.6	99.9	107.8	103.9	94.9	100.3	101.1	84.5	94.1	88.1	97	95.7
P05155	SERPING1	99.2	92.2	113.7	105.9	103.8	91.1	113.6	95.4	117.2	111.3	106.1	101.3	82.4	84.3	97	85.5
P06681	C2	86.6	91.3	105.2	86.3	118.2	86.1	92.9	105.8	111.8	91	109.3	111.3	98.4	116.4	104.2	85.1
P00736	C1R	102	88	94.5	83.6	85.3	102.8	93.9	105.1	113.8	98.7	114.8	108.2	107.6	106.1	93.5	102
P03952	KLKB1	82.8	113.7	107.8	134.2	110.7	90.2	68.4	108.7	80.7	97	144.3	97	96.7	97.1	74	96.5
P05546	SERPIND1	105.7	121.3	101.3	84.8	122.3	95.9	105.1	103	91.5	111.5	94.8	83.8	83.6	88.8	105.8	100.9
P02765	AHSG	100.5	91.4	106.1	90.2	115.2	116.4	96.6	108.2	124.2	119.6	110.2	86.4	93.5	94.6	62.1	84.7
P27169	PON1	128.7	92.3	110.8	129.1	102.8	123.7	87.7	135.3	89.7	122.7	100.6	78.3	94.8	56.5	80.6	66.3
P01859	IGHG2	112.4	58.7	60.5	162.7	67.4	137	97.6	239.3	53.4	56	36.9	136.1	147.3	122.3	56.3	56.1
P35908	KRT2	86.6	88.6	84.6	71.7	164.1	129.9	99.8	75.1	88.3	119.2	121	67.2	131.6	86.3	106.8	79.3
P02652	APOA2	113	86.1	103.8	112.1	97.7	103.7	100.8	128.6	77.4	143.8	135	100.3	77.7	74.9	61.4	83.5
P0DOX6		113	73.5	107.2	20.4	24.7	104.6	114.9	152.8	192.4	28.7	146.6	205.4	186.3	26	24.1	79.3
P02748	C9	83.8	114.6	103.5	96.9	80.9	82.2	132.4	70.2	112.8	72.6	88.4	101.7	101.3	125.7	137.7	95.2
P02760	AMBP	104.1	89.1	112.8	116.2	109.6	103.1	112	90.1	99	96	97.1	78.8	81.5	148.1	88.6	73.8
P0DOX2		92.7	74.8	105.8	132	124.8	140.2	109.1	83.7	90.4	85	80	141	117.6	62.2	97.1	63.7
P10909	CLU	95.7	93.4	104.2	97.6	100.4	99.6	108.3	101	122.5	123	128.5	95	68.7	89.6	88.4	84.3
P02649	APOE	82.6	98.2	73.7	92.3	120.2	86	90.4	117.8	89.6	122.8	144.9	103.9	106.1	88.9	94.8	87.9
O75882	ATRN	113.1	98.3	100.1	114.3	110.5	107.3	93.7	99	120.5	104.7	125.4	84	94.3	68.7	78.9	87.3
P25311	AZGP1	101.8	74.9	118.6	106.3	100.5	86.2	93.2	100	130.5	105.5	90.1	89	82.7	131.6	107.3	81.8
P07358	C8B	95.5	123.8	112.7	92.9	93.4	64.4	93.7	105	84.5	84.2	111.3	117.2	112.2	95.4	107.5	106.4
P05160	F13B	101.7	95.7	99.8	108.3	86.3	107.4	102.7	95.5	100	115.3	101.7	91.5	75.9	113.9	82.9	121.3
P02763	ORM1	85.5	106.5	78.3	112.1	93	76.7	156.7	78.6	101.9	77	103.3	98.9	78.3	130.1	127.6	95.6
P07357	C8A	92.8	119.1	109.3	91.5	88.9	71.8	99.9	104	86.8	85	110.8	114	112.4	102.5	101.4	109.8
P0DOX5		115.2	103.7	80.6	109.5	86.3	101.2	111.1	189.9	54.8	51.2	45.9	79.7	248	89.2	70.9	62.8
P02753	RBP4	89.9	73.1	110.5	103.2	93.1	98.5	88.1	104.6	115.2	115.9	109.6	73.1	75.9	183.3	88.1	78.1
P04217	A1BG	93.3	101.2	104.5	112.5	107.2	104.9	114.2	112.5	96.8	102.4	105.1	95.2	89	82	83.1	95.9
P80108	GPLD1	128.4	90.6	126.4	109.8	85.6	106.4	101.4	98.2	139.1	113	107.3	86.5	70.9	66.1	86.8	83.5
P01877	IGHA2	57.8	67.4	125.2	76.6	63.4	141.1	94.1	116.4	86.8	127.7	63.7	203.8	119.2	91	100.8	64.9
Q96PD5	PGLYRP2	104.2	92.6	107.3	103.3	111.3	100.1	91.5	118	99.1	102.8	105.3	90.1	117.7	82.2	81.5	92.9
P05543	SERPINA7	96.6	99	80	130.2	124.9	102.3	124.3	117.8	94.2	96.2	103.5	99.2	101.2	56.8	77.1	96.6
P23142	FBLN1	123.1	74.7	126.2	113.3	91.1	120.7	89	92.3	86.6	99.1	78.8	91.3	115.9	104.1	94.1	99.5
P07225	PROS1	96.7	94.1	110.5	92.9	126	92.4	97.1	120.2	103.5	98.9	113.4	93	89.2	94.1	92	85.9
P29622	SERPINA4	84.5	75.2	123.9	96.9	114	76.5	70.3	164.6	121.7	105.7	109.6	101.4	114.4	98.8	73	69.7
P04004	VTN	109.4	110.4	101.2	107.6	100.6	94	107.6	99.2	113.7	103.5	115.2	95.2	100.9	72.6	84.3	84.6
P68871	HBB	148.8	112.3	151.1	200.1	68.4	81.7	63.3	102.7	55.1	100	63.5	98.5	64.9	37.7	117.1	134.8
P00742	F10	96.1	104	115	111.5	98.8	109	113.3	93.1	106.1	112.8	111.5	82.5	84.9	79.9	89.3	92.4
P08185	SERPINA6	110	101.2	107.3	110.6	102.3	96.9	109.6	100.3	110.6	104.7	112.6	93	98.9	76.5	80.6	85
P0DOX7		102.9	77.1	98.4	128.8	106.5	128.7	113	107.9	76.5	85.8	72.3	127.1	149.7	70	85.5	69.9
P01861	IGHG4	83.8	60.9	64.6	62.5	63.4	379.4	70.2	262.4	34.4	57.8	35.2	66.5	55.3	240.9	33.1	29.5
P04196	HRG	67.5	62.6	86.1	310.7	99.3	130.5	106.9	92	52.1	83.5	130.3	115.6	89.4	74.8	51.8	47.1
P02750	LRG1	94.1	111.2	107.1	100.3	87.8	85.8	139.1	84.3	88.9	76.1	79	93.4	82	109.8	154.5	106.7
P19652	ORM2	97.9	108.2	99	100.5	102.3	91	145.9	100.9	90.3	92.8	113.7	89.3	75.5	84.8	100.2	107.7
P02766	TTR	102.4	89.2	117.2	115.1	96.6	110.6	102.9	104.2	131.2	149.1	100.5	83.6	73.6	74.7	75.4	73.7
P00488	F13A1	101	91.4	94.9	103.6	89.1	117	99.6	93.6	91.3	124.2	98.3	91.6	75.1	130.2	83.4	115.7
Q96KN2	CNDP1	110.6	119.4	92.6	88.7	101.1	78.3	110.9	113.5	101.1	116.3	113.8	98.7	111.8	77.6	72.5	93.3
P02749	APOH	83.3	71.1	241.4	68.4	92	53.5	54.2	138	91.2	59.1	75.6	90.1	205.5	149	76.7	50.8
Q12805	EFEMP1	98	71.7	104.1	120.1	115.4	111.6	105.3	97.7	88	88.4	71.3	89.6	99	139	105.5	95.7
P01834	IGKC	98.1	78	97.2	122	107.5	130.2	114.3	104.6	81	87.3	70	135.1	152.6	67.2	83	71.8
P43251	BTD	111.5	105.1	105.7	103.7	103.8	103.2	106.4	110.4	105.7	119.5	109.3	86.6	82.2	71.1	77.3	98.5
P36955	SERPINF1	99.4	86.4	93.7	75.3	93.3	53.6	61.7	135.2	104.7	70	80.1	112.5	121.4	228.5	106.5	77.9
Q08380	LGALS3BP	99.2	107.9	87.5	84.1	108.4	105.7	106.2	120.1	60.6	68	94.7	157.7	88.3	92	113.1	106.5
P35858	IGFALS	95	113.5	108.2	93	108.3	81.4	86.7	148.8	120.7	105.9	130.8	77	103.7	92	60.1	75
P02042	HBD	151.2	111.7	140.9	152.1	69.9	71.5	63.6	105.9	62.2	95.5	69.6	108.5	74.5	55.2	119.6	148.1
P51884	LUM	125.1	71.6	135.9	114.1	96.4	124.7	91	95	88.6	105.8	78.4	86.5	104.7	124.9	68.6	88.7
O14791	APOL1	102.5	133.1	93.8	78.5	97.3	87.7	124.1	115.7	96	102	138.7	118.4	87.8	76.1	67.7	80.5

P08519	LPA	60.9	84.3	208.5	87.8	52.8	59.8	97	54.1	141.3	162.9	52	62.4	69.4	54.6	219.1	133
O43866	CD5L	122.4	124.4	88	82.6	82.9	108.6	126.3	102.6	82.2	145.8	85.4	85.2	144.3	74.6	79.6	65.1
P02786	TFRC	90.9	96.3	96	95.5	108.7	112.3	125.3	93	108.7	123.3	97.7	80.6	111	97.8	84.6	78.4
P0DOY2	IGLC2	116.3	73.2	86.1	107.2	116.7	131	122.3	104.5	81	81.6	69.3	137	154.1	62.8	86.1	70.7
P0DOX8		104.6	78.7	108.3	99.7	114.3	131.5	113.4	91.5	83.5	81.6	77.2	129.9	159.9	57.7	103.5	64.9
P00748	F12	87.2	90	81	135.2	126.2	93.2	122	107.6	95.3	89.7	128.4	86.6	87.9	106	89.9	73.6
P22792	CPN2	118.7	104.6	103.9	123.1	112.8	93.8	91.7	106.6	107.2	117.8	101.2	88.3	87.9	68.6	99.8	74
Q9Y6R7	FCGBP	91.4	90.4	127.2	114.2	85.3	175.4	87.1	116.7	73.7	98.6	78.1	88	86.7	105.1	101.4	80.7
P07360	C8G	91.8	107.7	99.8	100.9	94.1	72.6	103.1	102.5	87.5	83.3	118.3	110.9	112.9	101.1	100.8	112.8
Q04756	HGFAC	94.8	97.1	93.3	92.4	102.7	106.3	110.9	136.1	96.9	110.7	105.9	86.1	106.9	99	84.7	76
P05452	CLEC3B	119.9	83.4	103	121.8	96.9	117.5	105.6	108.1	112.1	98.9	90.7	92.5	101.5	96.9	68.8	82.4
Q14520	HABP2	107.4	106.4	103.4	73.8	99	94.7	127.5	89.9	112.8	98.3	109.3	85.7	102.4	96.8	103.3	89.4
P00740	F9	106.7	112.7	97	95.3	102.1	102.4	98.3	91.5	102.6	110.8	104.6	94.2	115.2	78.3	101.4	87.1
P60709	ACTB	76.2	72.6	75.7	75.4	179	88.8	88.2	94.9	68.9	85.5	98.4	174.1	88	114.1	113.7	106.5
P05090	APOD	96.9	100.6	103.7	100.8	90.6	120.1	112.6	98.6	112.6	107.9	92.2	87.3	78	94.7	130.1	73.5
P05156	CFI	105.9	86.5	93.8	86.7	84.8	92.6	92.3	149.9	137	74.8	107.4	99.8	133.9	112.8	73.5	68.2
P04278	SHBG	96.4	80.5	113.8	136.1	110.5	151.2	93	114.1	110.7	109.7	78.3	95.8	107.5	54.1	78.8	69.5
P15169	CPN1	108.5	101.3	100.7	113.5	110.5	94.5	92	110.5	108.8	110.8	106	95.1	97.4	70	102.7	77.8
P02655	APOC2	59.9	93.5	95.6	77.6	93	67.8	74.6	128.1	81.2	147.2	253.8	106.6	75.1	104.1	77.3	64.6
Q9UK55	SERPINA10	106.4	106.1	116.9	70	87.6	96	94.6	122.2	105.8	87.1	115.1	106	105.4	79.3	108.7	92.9
O75636	FCN3	111	91.7	125.6	119.6	72.5	111.4	90.1	102.3	137.9	51.9	91.4	130.5	66.2	104.6	95.7	97.6
P13647	KRT5	86.1	81.8	101.7	85.3	118.9	141.7	112.6	83.2	96.3	124.7	120.4	73	132.3	68.7	95.1	78.3
P02538	KRT6A	90.7	73.2	113.8	71.4	88	102.8	109.4	85.6	110.4	128.5	119.8	66.4	190.9	65.3	89.5	94.2
P06276	BCHE	126	94	110.2	109.3	108.3	104	105.8	109.9	111.4	105.7	108.9	81.5	92	67.9	75.9	89.2
P0DOX3		88.5	87.6	113.4	184.2	52.3	149.4	151.3	144.1	84	59.8	48.8	91.3	111.2	52.9	51	130.1
Q92954	PRG4	114.3	119.5	80.7	83.2	115.1	94.7	91.3	100.5	93.1	98.2	150.5	108	88.8	51.3	89.3	121.8
P0DJI8	SAA1	35.5	69.3	36.8	37.2	38	32.4	55.3	42.3	32.4	35	46.3	107	31.2	71	895.6	34.8
Q15582	TGFBI	104.8	97.7	113.2	97.8	94	118.2	104.8	106.4	102.1	102.4	125.1	109	64.9	75.2	81.2	103.1
P01019	AGT	96.2	89.9	88.8	96.8	116.3	102.6	98.9	101.1	116.5	107	120.1	98.4	92.4	77.7	105.4	91.9
P48740	MASP1	112.9	90.4	110.9	102.4	81.1	103.5	102	107.4	108	89.2	105.5	114.1	88.7	121.2	82.6	80.4
P02743	APCS	102.4	103.6	90.2	94.4	84.7	77	114.8	76.8	113.3	105.4	118.7	106.3	82.6	100.2	120.8	108.9
A0M8Q6	IGLC7	125.3	99.5	94.6	88.2	80.5	98.9	57.3	91.9	260.2	40.3	28.1	165.6	106.8	49.7	125.6	87.5
P02533	KRT14	92.8	94.3	118.6	79.6	108.1	107.6	113.1	88.5	89.1	120.5	133.9	82.2	133.5	66.4	88.1	83.7
Q9UHG3	PCYOX1	101.1	101.3	103	125.1	90	109.1	113.8	131.4	100.6	118.2	101	86.5	89.9	70.5	89.3	69.2
P04070	PROC	96.4	104.5	108.1	124.6	110.1	91.4	87.5	104.3	107.6	112.3	120.1	83.3	98	79.3	79.7	92.8
P01591	JCHAIN	120.7	137.2	99.8	72.5	93.5	116	127.3	98.1	76.7	135.2	73.5	114.6	131.9	53	79	71
Q9UGM5	FETUB	118.6	102	116	120	99.9	113.2	106.8	105.8	112.9	100.2	99.8	90.6	89.1	71.7	67.9	85.7
P05062	ALDOB	66.2	74.5	68.1	62.8	67.8	61	64.4	508.5	67.9	99.7	122.4	74.4	68.5	61.6	80	52.2
O00533	CHL1	108.3	91.7	94.4	128.8	94.6	110.9	104.5	111.9	99.4	104.9	85.2	91.8	94.7	93.4	97.5	88.2
Q16610	ECM1	103.9	104.6	110.1	78	105.8	72.7	73.3	120.5	116.8	102.6	79.7	113.8	109.1	111.9	86.2	110.8
P02745	C1QA	128.8	98.6	110.9	112.5	129	90.8	92.5	117.3	86.9	67.3	96.2	100.2	98.1	84	90.9	96.1
P13796	LCP1	104.6	97	86.8	114.3	102.2	111.5	122.7	97	91.8	100.7	99.5	90.6	96.2	105.7	84.3	94.9
P02741	CRP	95.7	199	24.4	36.4	45.1	23.3	87.3	26.9	46.7	23.2	62.9	200.3	58.1	91.2	494.5	85
P32119	PRDX2	131	110.4	129	179.5	74.5	81.3	70.5	93.1	69.1	96.6	74.2	102.2	84.8	56.9	107.2	139.6
P03951	F11	69.6	59.9	62.2	168.9	66.5	160.6	210.8	84.7	63	78	169.2	144.4	60	88.9	51.8	61.5
P20851	C4BPB	80.6	102.4	102.5	82.9	162.2	67.6	78.7	128.6	113.3	86.3	106	98.7	99.5	107.8	100.8	82
P0DJI9	SAA2	66.6	91	67.9	56.7	79.7	73.9	108.1	61.9	50.3	68.1	65.7	106.5	83.3	69.4	489.6	61.1
P19320	VCAM1	112.7	80.9	92.8	97.6	114.7	96.6	112.2	101.6	82.8	105.9	99	95.5	97.5	99.3	137.8	73
P02656	APOC3	83.5	85.2	88.4	81.2	107.5	72.5	73	114.8	74.8	135.6	190	93.5	84.1	128.4	106.9	80.7
P11021	HSPA5	108.7	100	101.7	99.2	102.1	98.6	100.4	100.2	95.4	103	104.4	96.1	93.1	103.6	100.5	93.2
P13591	NCAM1	112.2	79.5	139.5	99.9	94.3	136.2	98.4	100.3	105.2	100.7	86	82.9	98.3	87.9	94.5	84.2
Q03591	CFHR1	85.7	122.8	100.7	104.5	83	63.8	73	149.6	82.2	89.1	127.7	104	112.4	119.2	116.7	65.7
P69905	HBA1	143.2	112.9	143.8	180.5	75.5	78.8	66.3	103.9	62.2	101.4	70	96.3	67.8	44.7	113.6	139
Q9NZP8	C1RL	98.6	109	90.4	113	97.2	101.1	118.4	90.1	103.8	89.5	94.7	88.3	100.6	113.6	108.2	83.7
P04180	LCAT	109.8	104.4	94.5	116.4	110	105.5	97.9	104.6	104	106.2	122.7	94.2	91.8	77.6	76.5	83.9
Q9NQ79	CRTAC1	99	87.5	89.2	114.5	100.6	101.8	98.8	104.3	98.3	85.2	81	87.6	112.7	106.4	97	136.3
P08571	CD14	102.8	92.5	106.6	112.6	99.9	108.8	112.8	89.7	110.6	93.9	87.1	103.4	83.4	94.3	104.4	97.1
P11226	MBL2	126.2	82.9	108	95.7	64.7	69.1	184.9	110.3	84	70.5	60.8	139.8	92.5	146.1	64.6	100
P33151	CDH5	133.8	69.5	117.4	120	128.6	92.2	100.8	100.4	97.1	109.4	93.5	93.2	82.2	99.8	84.2	78.1
P02746	C1QB	127.9	102.7	112.4	116.7	126.3	87.1	92.9	112.6	80.3	71.5	93.1	96	95.1	89.7	91	104.9
P01833	PIGR	98.1	122.9	121.5	92.3	85.6	87.3	96.2	87.1	109.4	109.7	105.2	124.5	80.9	92.9	102.1	84.3
P15144	ANPEP	104.7	98.6	89.1	111.1	100.8	123.1	106	119	111.7	103.7	92.8	93.9	90.3	82.5	91.9	80.7
P06702	S100A9	62.8	61.7	62.8	82.7	86.1	86.1	111.4	61.1	53.9	84.1	93	67.4	113.5	369	119.9	84.5
P55058	PLTP	123.4	92.3	100.3	105.2	111.8	96.8	94.1	103	93.1	103.4	93	113.1	99.5	98.1	78.9	93.9
P81605	DCD	77.7	72.7	61	169.5	64.1	209.8	196.1	69.8	50.9	94.6	80.3	68.1	116	92.5	94.9	82
Q86VB7	CD163	99.8	93.9	115.7	86	120.9	111.5	118.8	90.7	77.1	98.3	73.7	96.4	99.3	101.1	145	71.7
P98160	HSPG2	106.7	87.2	102.3	109.3	92.6	103.3	104.1	102.7	92.6	109.6	92.9	93.9	102.2	128.6	86.3	85.5
P33908	MAN1A1	112.6	94	98.4	101.8	104	103.8	104.4	94.5	99.5	99	86.4	105.8	101.6	92	104.7	97.5
P05154	SERPINA5	79.3	73	97.7	144.6	95.6	100.4	74	129.6	127.4	84.4	123.8	94.4	131.6	89.4	66.1	88.7
P63267	ACTG2	78.5	62.8	60.2	63.6	196.1	108.6	86.7	95.7	69.7	84.7	94.2	200.2	81.8	104.3	114.2	98.7
Q6EMK4	VASN	112.4	76.8	111.1	98.3	107.8	100.8	99.8	108.6	107.3	104.8	87.2	97.4	88.9	125.2	84.9	88.6
P14151	SELL	102.2	96.5	85.1	111	107	113.4	132.4	106.6	114.6	90.7	95	95.6	94	86.8	93.5	75.5
P35542	SAA4	86.4	102.4	94	90.3	95.6	71.9	111.5	114.7	87.5	102.6	141.8	111.5	92.2	99.7	114.3	83.7
P22105	TNXB	120.3	90.6	103.3	96.7	100.3	121.1	100.6	104.5	105.4	101.6	97.6	94.6	93.3	99.1	87.3	83.7
P18428	LBP	75.9	91	90.8	65.4	108.5	97.3	121.3	120.6	113.4	69	94.6	127	139.1	102.6	103.1	80.3
P22352	GPX3	103.3	107.1	112.2	111.1	88.5	140.9	96.3	129.2	77.6	118.6	84	108.8	106.2	49	88	79
Q14515	SPARCL1	93.1	79.9	105	89.8	96.5	99.3	83.3	106.7	76.9	97.6	90.4	83.8	88.3	206.9	120.2	82.3
O95445	APOM	97.7	102.9	108.1	123.6	98.9	98.3	94.8	110.8	117.5	118.1	137	91	74.3	70.8	72.7	83.7
P22891	PROZ	117.2	97.8	127.1	63	72.6	110	107.5	113.5	111.2	90.8	121.9	98.1	81.8	77	86.5	124.2
P11597	CETP	125.2	124.1	82.1	86.3	109.2	95.1	97.3	120.2	98.2	92.7	89.6	88.7	156	82.6	76.7	76
Q92820	GGH	99.4	92.2	104.9	111.3	91	87.1	117.5	131.9	89.6	83.6	106.3	113.5	117.5	94.6	85	74.5
A0A0C4DH38	IGHV5-51	114	93.5	56.8	76.3	157.2	124	86.4	101	75.8	103.7	78.7	110	150.6	78.6	92.1	101.2
Q15166	PON3	116.2	90.8	101.9	118.9	80.1	89.4	110.9	127	110.6	121.7	101.9	85.5	92.8	81.2	87.8	83.4
P00918	CA2	128.9	113.5	145.8	164.4	71.6	73.4	63.4	94.5	72	102.3	61.4	101.8	91.6	64	124.6	126.9
P02654	APOC1	79.6	109.2	103.8	90.4	102.7	88.2	73.6	143.7	104.6	122.8	151.4	85.7	107.6	102.6	68	66.1
P17936	IGFBP3	82.3	102.5	104.4	93.1	107.1	64.5	70.5	154	129.8	94.9	139.5	80.7	106.6	144.4	56.8	68.9
P08779	KRT16	108.6	79.5	151.2	65.2	96	79.5	104.7	91.7	93.7	128.8	124.4	83.6	125.9	63.2	96.2	107.8
P08709	F7	102.8	94.2	109.1	111.2	107.3	87.1	87.3	113.2	101.6	109.9	103.5	101.5	94.5	102.2	84.8	89.9
P02747	C1QC	128.1	107.4	106	104.7	126.8	84.9	87.2	120.5	77	74.3	100.8	96.7	98.3	92.5	91.7	103.2
P36980	CFHR2	66.2	103.3	78.2	95.7	68.9	137	62.7	159.7	78.9	131.7	110.7	79.1	116.3	110.2	122.5	78.8
P55056	APOC4	69.5	101.6	93.2	79.1	95.4	51	81.4	109.9	95.2	125.2	203.4	103.3	95.6	114	104.5	77.6
Q14126	DSG2	98	81.2	118.4	97.7	111.3	91.4	94.5	106.1	111.9	105.7	92.7	96.8	92.7	101	107.4	93.2
Q9BXR6	CFHR5	96.1	91	103	107.6	86.8	87.8	103	94.4	92.8	89.7	111.6	117.4	116.9	112.5	104.1	85.4
P49908	SELENOP	92.5	90.2	116.2	92.3	110.6	95.3	69.1	171.5	102.7	105.1	117.5	89.2	102.3	71	101.8	72.5
P01768	IGHV3-30	100.6	99.7	87	97.5	91.2	158.7	108.5	110.6	84.6	86.8	88.9	102	158.8	88.2	74.6	62.2
P01619	IGKV3-20	85	121.3	111	91.3	115.7	109.8	98.1	82.2	86.7	106.4	55.7	153.4	164.6	69.5	91.7	57.4
P01780	IGHV3-7	108.2	88.6	92.6	122.6	112.8	129.2	124.1	100.9	73.5	93.2	62	126	160.2	63.5	80.3	62.3
P01764	IGHV3-23	125.3	149.5	105.3	96.7	110.8	126.2	123.9	90.1	85.6	98	79	97.6	92.1	70	83.1	66.8
O00187	MASP2	111.6	94.6	117.2	82.8	86.1	98.9	108.8	101.1	111.5	79	100.3	101.1	79.6	118.8	101.9	106.7
P00915	CA1	127.3	139.1	144.6	148.5	54.3	49.9	28.4	154.6	55.7	91.7	64.1	129.7	113.5	55.6	130.5	112.5
P07996	THBS1	81	117.4	189.1	48.3	118	119.8	59.8	194.5	53.4	79.4	67.2	78	58	61.3	106.6	168.2
P01762	IGHV3-11	104.1	111.1	90.6	114.4	106.6	142.9	97	106.2	101.7	95.9	86.7	88.8	112.6	75.4	75.9	90.3
P09172	DBH	126.2	87.5	80.9	120.3	98.1	164.2	137.4	94.6	81.6	64.2	82.8	87.1	97.3	83.8	104.8	89.3
Q13103	SPP2	95.7	110.4	97.6	98.6	115.7	100.6	65.8	136.3	91.1	113.6	92.7	109.5	104.5	80.7	105.7	81.7
A0A0C4DH25	IGKV3D-20	122.4	83.4	86.9	111	107.9	132.9	107.2	93	85.4	97.5	73.2	125.8	151.7	68.8	85.4	67.5

P61769	B2M	66.7	53.5	67.2	75.6	77.3	71	86.2	59.4	63	65.8	62.5	47.4	64.3	589.3	100.5	50.2
Q6UXB8	PI16	126.1	72.2	126.2	136.5	107.6	104.9	95.2	102.7	95.4	103.8	79.6	85.7	87.5	113	72.7	90.7
P49747	COMP	108.6	79	98.7	104.6	102.8	118	100	101.7	106.5	122.2	93.7	82.8	79.8	128	72.6	100.9
P08253	MMP2	115.8	85.6	106.3	108.1	107	113.9	90.2	116.6	89	99.8	94.2	88.6	108.8	101.4	94.4	80.2
A0A087WW87	IGKV2-40	109.8	96.3	88.9	92.2	94.4	109.7	126.9	104.7	80.2	104.5	70.9	103.4	198	74.9	78.8	66.3
Q01459	CTBS	101.6	97.9	111.1	88.7	97.4	87.3	94.4	101.8	117.6	117.3	91.3	99.8	96.1	101	96.2	100.5
Q13790	APOF	104.4	102.6	103	106.7	98.9	100.5	102.5	100.7	77.4	119.7	103.3	84.4	102.6	74.8	133.4	85
P05109	S100A8	65.3	68.1	68	85.8	98.3	86.9	103.5	62.3	67.9	86.3	89.1	70.2	97.1	345	123.2	82.8
Q86UD1	OAF	92	80.9	92.6	103.5	113.7	94.1	100.1	91.7	106.5	86.5	103.9	110	79.8	130.1	141.9	72.7
O15485	FCN2	91.2	132.7	107.9	155.3	65.9	87.4	86.7	116.7	148.9	113.3	107.1	71.9	53.7	63.4	72	125.9
Q8NBP7	PCSK9	94.8	99.4	123.1	107.5	93.4	105.7	107.4	149	76.5	95.6	96.4	95.7	87.1	74.7	107.1	86.7
P41222	PTGDS	64.5	59.2	79.2	68.9	71.9	85.8	85.8	65.6	80.7	79	61	66.1	73.7	508.5	90.9	59.1
Q6UX71	PLXDC2	102.7	92.9	116.3	102.9	100.2	115.1	105.1	98.8	111.6	91.7	83.7	81.2	105.1	128.9	90.7	73.1
P69891	HBG1	100.7	81.1	112.5	95.9	84.5	85.8	128.9	236.1	97.4	89.1	79.4	92.8	76.7	64.1	68.5	106.4
P0DP04	IGHV3-43D	117.8	98.6	82.4	120.1	82.4	149.8	139	96.2	75.3	89	66.9	121.8	121.1	66.1	90	83.8
P43121	MCAM	104.4	71.5	121.8	106.1	106.4	117.8	101.5	83.5	92.3	154.5	82.5	69	93	81	81.3	133.5
P18206	VCL	91.6	82.9	85.6	90.1	132.1	106.3	108.2	104.7	79.1	87.9	95.9	126.7	92.9	97.1	113.5	105.2
P01714	IGLV3-19	122.1	95.3	80.8	107.5	113.5	88.1	142.1	83.7	77.3	85.7	86.4	131.8	152.2	62.2	97.2	73.9
Q7Z794	KRT77	94.3	80.5	79	82.1	112.8	97.6	96.1	85.7	91.2	125	101.8	103.2	149.8	94.4	91.4	115
P01700	IGLV1-47	152.9	102.3	85.3	102.8	98.6	124.2	130	92.6	63.9	84.8	69.6	131.2	146.2	66.1	94.2	55.1
P10645	CHGA	102.9	68.5	116.5	86.8	81.6	53	54.5	102.8	53.4	136.7	64.4	84	105.2	304.8	114	70.9
A2NJV5	IGKV2-29	104.6	83.5	48.4	114	134.2	169.8	168.9	86.1	68.9	84.7	43.5	62.7	240.9	44.7	88.5	56.5
Q9BTY2	FUCA2	108.2	77.5	102.5	108.6	103.2	126.1	107.4	113.9	95	112.6	102.8	97.5	101	70.7	92.3	80.8
A0A0C4DH31	IGHV1-18	101.7	83.8	107.3	96.1	82.6	95	120.5	88.3	117.3	116.3	101.7	118.3	98.4	97.3	100	75.4
P23083	IGHV1-2	81.9	66.8	82.5	124	140	118.3	151.3	69.6	72.2	91.4	81.6	107.6	179	71.5	102.7	59.7
P01743	IGHV1-46	75.8	101.6	77.9	119.3	112.8	101.8	119.7	101.4	91.4	112.5	73.1	105.2	162.5	75.3	103.1	66.6
Q9HDC9	APMAP	100	94	98.4	107	110	103.8	102	66.2	111.8	111.3	108.4	106.8	109.9	86.2	88.3	95.7
P01624	IGKV3-15	69.2	101.9	110.2	74.3	99	89.7	185.1	92.2	74.1	94	66.5	143.9	146.2	89.1	95.8	68.9
P06331	IGHV4-34	104.1	93.7	96	125.7	100	102.9	105.5	90.8	72	110	76.4	158.1	158.6	62.3	87.7	56.2
P01893	HLA-H	69.6	66.3	67.1	64.1	84.2	70.7	81.3	82.3	90.6	164	248.2	151	67.6	153.2	66.8	72.9
P54108	CRISP3	96.6	93.9	82.4	142.7	109.7	93.9	106.3	92.9	107.8	105.9	82.1	100.8	112.7	93.4	96.6	82.3
P04406	GAPDH	99.2	87.6	96.9	102	120.5	110.5	96.9	117.4	84	89.7	85.4	110.1	132.8	67	102.4	97.5
P26927	MST1	89.4	84.5	92.4	143.1	96.7	112.5	108.9	116.7	94.6	97.6	110.1	101.8	103.3	107.6	76.5	64.3
Q6UWP8	SBSN	146.7	77.8	88.2	109.6	120.6	99.6	124.6	85.6	71.1	83.5	99.7	98.9	107.6	106	103.7	76.7
O75144	ICOSLG	110.3	97.7	93.1	114.9	99.5	115.1	102.2	108.8	107.8	125.1	87.1	94.4	81.3	88.6	80.5	93.4
Q6YHK3	CD109	113	79.1	145.3	84.2	98.2	123.7	107.3	74.7	88.3	122	101.8	100.1	82.1	88	91.4	100.9
P13598	ICAM2	114.3	81	105.3	111.1	112.2	122.4	132.7	107.7	100.8	112.1	98.2	78.7	84.8	94.7	61.3	82.6
P01593	IGKV1D-33	75.6	85.1	109.4	104.7	95.2	113.1	120.3	139.2	72.8	72.9	94.1	120.6	125	106.7	98.3	66.9
P06312	IGKV4-1	107.2	100.1	64.8	84.9	113.7	130.2	112.2	101.3	78.7	121	83.2	121.2	161.1	75.1	80.2	64.9
P27797	CALR	94.6	72	104.8	93.5	110.4	92.7	103.2	89.6	82.2	82.7	83.8	102.5	80.6	199.6	123.9	83.9
P0DP01	IGHV1-8	105.5	116.1	98.9	101	93.5	83.8	137.1	80.9	100.5	122.4	73.8	126.4	146.2	70.7	69.6	73.5
P08294	SOD3	94.3	94.8	99.5	84.5	97.3	96.6	95.1	139.8	84	98.8	86	94.5	100.1	92.9	152	89.8
Q02985	CFHR3	95.5	107.1	73	93.4	81.3	71.1	83.2	148.8	81.7	70.8	92.4	90	170	178.3	95.1	68.4
Q08830	FGL1	88	100.6	110.3	67.3	68.9	121.3	97	60.8	83	68	73.1	116.4	85.4	80	253.8	126.2
P80748	IGLV3-21	78.5	76.2	67.5	100.2	137.4	142.4	201.2	69.4	72.1	95.9	85.8	129.4	126	65.7	88.6	63.9
Q04695	KRT17	52.6	50.7	89.1	48.1	59.4	45	71.9	70.6	67	73.4	158.7	33.4	596.8	43.1	66.6	73.5
P16070	CD44	118.9	78.9	108.3	121.8	92.7	114.5	127	99.4	106.1	94.6	78.7	85.8	84.3	121	86.4	81.4
P00746	CFD	65.3	77.6	81.2	88.2	75.4	75.8	83.8	104.6	72.3	89.6	87.8	108.6	111.3	324.9	86.6	66.8
P27918	CFP	101.6	105.4	83.3	113.7	155	71.9	78.6	140.2	99	57.9	102.3	93.8	123.4	101.1	85.9	86.8
P01721	IGLV6-57	90	90.5	102.6	78.2	107.5	93.8	111.9	109.3	80.4	99.3	90.2	122.3	139.9	107.9	105	71.3
Q9Y4L1	HYOU1	106.1	74.9	103.4	104	113.4	87	105.4	92.6	87.8	100.7	89	85.7	97.1	166.1	100.8	86.1
P12111	COL6A3	99.9	98.4	108.9	92.3	102.7	94.3	94.9	101.2	111.5	94.1	89.6	78.3	115.5	149.7	89.9	78.7
A0A0B4J1Y9	IGHV3-72	106.8	92.4	96.5	102.1	97.8	123.4	109.4	104.9	88.9	107.4	74.6	119.6	130.3	78.7	78.1	89.2
A0A075B6K4	IGLV3-10	139.5	53.3	51.3	155.8	171.8	169.7	102.6	70.1	91.2	85.4	73.8	114.4	113	46.1	75.7	86.5
Q9UNW1	MINPP1	104	84.1	124.6	100.9	105	113	111.6	103.4	116.4	111.1	111	83.4	91.1	81.4	72.9	86
Q9ULI3	HEG1	120.8	75.1	118.5	105.1	114.9	116.9	116.7	99.9	58.4	114.8	86.9	61.2	118.4	130.3	75.8	86.4
P31151	S100A7	80.4	68.3	72.7	111.8	68.8	126.6	206.4	68.1	71.2	147.5	128.6	70.9	150.5	64.1	76.1	87.9
O07954	LRP1	91.7	101.1	111.8	81.7	135.9	106.4	115.2	109.3	86.7	72.7	68.7	79.4	148.2	68.7	109.3	113.2
Q9NPH3	IL1RAP	98.1	105.5	105.1	99.4	96	84.8	93.6	113.1	103.5	107.7	92.1	92.2	112.7	95.1	101.2	100
A0A0A0MS15	IGHV3-49	119.9	95.3	69.7	89.9	96.2	105.6	103.1	147.5	67	89.5	76.9	177.8	136.8	68.4	103.9	52.5
P14625	HSP90B1	140.6	80.9	117.4	107.7	80	112.4	105.5	122.2	88.4	79.5	75.4	122.8	99.7	90.7	105.9	70.9
P07339	CTSD	100.8	98.2	93.7	98.1	91.2	95.7	117.2	97.7	105	100.5	109.1	129	102.8	75.4	83.4	102.1
P07333	CSF1R	142.1	87.6	82.7	98.5	113.6	107.9	96.7	106.8	64.9	113.1	78.3	110.4	115.6	108.6	102.4	70.7
P04211	IGLV7-43	81.4	218.7	53	63.9	74.9	147.9	156.6	71.2	49.3	180.3	93.5	90.1	143.2	46.3	91.3	38.4
P55290	CDH13	122.2	59.1	116.1	104.6	108.6	106.4	92.7	98.3	100.2	101.2	103.1	107.5	82.4	167.9	68.3	61.6
Q9Y5Y7	LYVE1	105.5	78.1	97.9	102.9	121.3	146	104.1	81	77.2	110.7	73.3	72.3	78.1	167.2	115.7	68.6
Q6Q788	APOA5	86.8	93.2	115.1	77	97.3	70.3	69.1	94.4	93.1	119.9	203.3	85	95.7	68	146.7	85
Q9BWP8	COLEC11	92.3	95.4	109.4	125.5	84.6	105.4	107.3	126.3	94.8	93	115	103	83.9	71.4	101.6	91
P01034	CST6	81.5	64	77	79.9	85.3	109.9	85.5	70	74.1	81.2	66	70.5	72.5	407.8	110.4	64.3
Q15063	POSTN	96.4	78.1	102.2	108.5	115	124.3	77.3	121.8	84.5	120.7	80.8	104.6	132.2	79.9	103.6	70.1
Q8WZ75	ROBO4	110.4	82.9	119.6	97.2	115.8	96	101.1	110.3	101.7	115.1	91	94.9	86	108.4	86.3	83.4
P02776	PF4	80	77.7	113.3	53.2	109.4	264.4	65.2	175.4	61	101.5	73.7	71.8	85.4	49.5	85	133.5
P35443	THBS4	96.7	86.4	100.2	103.8	99.1	85	103.1	114.7	107.1	82.8	107.2	99.7	105.4	132.6	94.8	81.5
A0A0C4DH34	IGHV4-28	90.8	69.3	76	112.8	109.9	151.6	82.8	85.2	79.6	91.7	79.8	158.3	172.4	65.3	99.4	75.2
P12830	CDH1	79.5	175.1	85.2	107.5	98.8	75.7	84	108.3	81.6	90.1	116.8	99.4	85.3	102.3	115.7	94.8
A0A075B6I9	IGLV7-46	110.1	138.3	56.8	92.3	104.9	122.4	121.6	116.7	71	116.4	69.9	96	160	56.5	87	80.1
Q15848	ADIPOQ	128.1	87.8	90.7	169.3	142.7	130.5	86.1	147.2	127.4	70.5	61	58.5	86.5	60.9	78.3	74.4
P15814	IGLL1	84	88.4	118.3	83.8	136.5	131	81.7	80.9	80.8	72.5	57.9	104.4	91.4	100.2	122.5	165.9
Q7Z7G0	ABI3BP	105.2	92	111.9	119.1	98.4	120.8	103	88.9	127.1	103.9	84.8	89.6	81.9	105.1	83.1	85
A0A075B6R2	IGHV4-4	181.8	89.3	74.3	54.4	145	58.8	129.7	88.6	62.7	154.8	87	82.9	99.7	72.5	126.6	91.8
Q9BUN1	MENT	68.4	96.1	119.4	94.6	77.5	73.2	90.9	100.4	91.5	100	90.6	118.1	88.7	135.8	107.4	147.5
A0A087WSY4	IGHV4-30-2	125.4	89.9	89.6	89.1	89	106.1	129.4	94.2	91.4	96.4	101.1	105.4	132.9	92.9	88.7	78.4
P04430	IGKV1-16	95.1	123.4	96.1	82.3	83.2	165.4	166.8	85.7	73	102	63.6	96.1	134.4	80.1	90.4	62.7
A0A075B6J9	IGLV2-18	91.4	112.6	87.9	74.2	95.7	164.3	105.3	84.8	82.3	93.3	84.2	119.7	153.9	68.9	105.2	76.3
A0A0B4J1Y8	IGLV9-49	150.2	75.9	73.6	94.9	91.5	192.5	64.4	94	93	99.3	109.3	120.4	137	52.2	73.2	78.7
P35916	FLT4	143.7	99.2	114.2	101.6	106.1	101.1	104.3	113	104.1	120.4	87.3	78.6	83.1	76.9	76.2	90.4
A0A0B4J1V2	IGHV2-26	100.9	133.7	65.6	77.9	106.5	125.4	199.1	105.5	68.6	75.3	82.5	180.1	80	77.8	83	37.9
O15204	ADAMDEC1	88.3	83.8	112	92.9	98.6	92.6	96.4	84.6	93.7	86.5	76.3	84.7	118.4	162.8	138.3	89.9
P07359	GP1BA	115.6	76.6	107.6	94.5	123.3	112.5	80.9	108.7	98.1	101.4	105.6	100.5	76.9	103	116.2	78.7
Q05639	EEF1A2	84.3	100.8	117.7	111.2	132.5	91.5	83.6	94.6	94.4	90.2	110.3	119.7	88.2	82.3	99.7	98.9
P18065	IGFBP2	90.4	74.1	104.1	106.8	98.3	93.5	85.7	103.5	90.9	103.6	94.5	93.1	99.9	153.3	129.2	79
P59665	DEFA1	89	74.2	78.2	117.5	103.6	110	77.9	68.4	83.3	82	97.8	70.1	85.9	275.6	107.3	79.2
P13473	LAMP2	99.7	101.5	119	120.9	111.6	114.9	107.3	103.4	101	115.8	103.3	80.8	86	68.2	80.1	86.5
P22692	IGFBP4	93.8	92.2	102.7	49.4	97.7	75.2	64.6	119.5	89.6	112.1	89.4	88.6	106.6	248.5	94.3	75.7
A0A075B6I0	IGLV8-61	54	154	30.4	57.3	161.3	98.8	199.8	32.1	98.1	185.9	88.9	114.4	137.7	65.2	50.7	71.4
P05362	ICAM1	102.3	95.1	95.5	122.4	104.5	107.1	104.2	103.7	79	112.3	92.6	117.1	96.6	80	91	96.6
P06733	ENO1	90.8	87.8	90.7	97.1	137.6	91.6	86.7	122.4	97.1	86.4	100.1	100.4	104.7	106.8	101.2	98.8
Q00610	CLTC	99.9	90.7	105	106	115.8	90.7	81.4	105.9	91.6	124.7	93.1	107.2	106.2	91.6	84.5	105.7
P27487	DPP4	116.2	93.6	97.9	102.7	103.6	113.8	105.4	112.5	122	101.3	102.2	71.8	95.9	82.5	68.7	109.9
O14786	NRP1	102.6	104.2	96.2	81.6	118.8	101.8	111.2	83.1	87.3	89.4	63.1	96.8	125.4	90	158.5	90
A0A075B6H9	IGLV4-69	94.1	83.5	96.2	118.6	113.6	139.3	118.3	99.3	181.7	93.4	60.5	156.5	37.8	60.8	68.4	78
A0A0J9YX35	IGHV3-64D	102.6	88.8	94.9	124	101.2	154.4	121.1	106.4	79.9	78.7	81.1	102.6	145.7	62.6	89.3	66.7
A0A075B6R9	IGKV2D-24	99.4	199.3	71.5	104.2	91.5	126.8	71.3	94.4	63.8	105.6	83.5	132.1	135.7	63.4	98.2	59.4
Q13201	MMRN1	71.6	112.9	165.3	54.5	88.6	162.9	53	182.1	57.3	92.8	91	73.2	70.8	73	112.7	138.2
P54289	CACNA2D1	97.3	79.5	136.4	121	86.8	147.8	79	96.3	115	98.8	95.6	85.2	79.5	123.3	85.2	73.1

Q3LXA3	TKFC	113	104.2	110.1	108.8	115.1	55.3	56.1	93.2	87.8	135.6	93.3	136.8	142.3	154.6	49.7	44.2
P12821	ACE	105.5	81.2	97.4	84.4	104.1	103.8	146.9	88	104.1	106	114.9	99.3	90.7	102.4	82.7	88.5
A0A0B4J1X8	IGHV3-43	84.6	92.7	87	111.4	104.1	139.4	113.7	91.4	75.4	67.6	83.9	114.3	172.8	84.7	86.1	90.8
Q96NZ9	PRAP1	76.7	95	110.7	86.1	75	64.7	73.8	77.4	75.8	89.3	92.1	100.4	80.6	231.9	117.8	152.7
O43493	TGOLN2	90.2	80.4	92.1	82.7	86.4	91	85.3	103.3	81.1	91.7	79.4	105.1	90	229.7	110.9	100.6
Q5D862	FLG2	78.5	50.3	75.7	47.1	99.3	115.3	92.4	67.4	64.2	107.8	125.1	56.1	435.9	52.2	59.7	73
Q7Z7M0	MEGF8	92.2	146	99.1	83.7	233.4	104	81.3	71.8	102.2	127.5	85	70.9	74	81	63.5	84.4
P0DPH8	TUBA3D	104.1	102.8	84.7	121.7	108.9	99.9	124.2	101.1	79.2	106.4	90.9	105.3	106.8	79.3	102	82.7
P55103	INHBC	91	84	95	95.1	100.8	101.7	129	85.5	114.2	102.2	97.4	95.5	129.8	84.7	115.4	78.5
P01742	IGHV1-69	113.8	88.4	89.4	116	89.3	135.9	127.2	93.5	99.4	82.5	80.7	108.2	122.8	49.8	59.1	143.8
P01034	CST3	67.2	78.2	75.9	71.9	74.8	61	70	83.9	72.8	73.8	63.6	61.8	74.3	513.2	103.1	54.7
Q16270	IGFBP7	90.8	97.5	107.9	89	116.5	97.5	92.4	101.3	90.3	95.5	94.6	91	102.8	133.4	110.1	89.4
P22897	MRC1	118.9	90.1	100.4	94.1	95.8	136.7	94.3	108.9	98.4	93.9	83.1	112.3	86.8	85.1	108.9	92.3
P17813	ENG	103	92.5	116.4	128.2	102.1	127.9	111.5	98.1	110.5	102.1	94.9	89.7	97.1	72.5	80.7	72.7
P04746	AMY2A	97.9	74.4	102.9	109.3	101.5	111.1	101.8	101.7	105.2	112.1	98.9	103.5	128.9	94.1	80.7	75.9
Q14118	DAG1	97.5	84.2	107.7	85.3	117	84.2	83.5	102	77.8	85.3	80.1	86.4	75.5	246.6	112.4	74.4
Q99714	HSD17B10	117.9	109	119.4	113.8	116	79.8	71.3	96.3	74.8	115	109.3	125.1	146.9	115.6	48.5	41.1
P07237	P4HB	111.8	88.4	84.4	96.7	112.1	87.2	85.3	116.9	97.2	120.6	103.8	118.2	104.5	99.3	98	75.5
Q8IXL6	FAM20C	86.8	91.7	117.8	86.1	99.2	145.3	105.9	103.1	89.5	80.8	92.6	89.3	124.9	88.6	106.5	91.8
A0A0C4DH72	IGKV1-6	101.7	93.6	83.1	121.4	125.6	60.9	109.3	129.2	93	100	95.5	159.1	94.9	90.5	74.5	67.7
Q12860	CNTN1	101.8	97.6	107	113.3	111.4	117.5	94.1	111.6	100.3	126	88.4	88.1	98.9	80.8	80.4	82.8
Q8WWA0	ITLN1	94.1	82.7	133.2	91.7	85.7	120.8	120.6	90.2	97.4	106.8	77.6	81.2	114.9	93.9	108.4	100.7
Q9Y6Z7	COLEC10	104.3	92.1	103.1	108.8	84.9	113.1	106.8	126.7	97.6	98.9	100.4	89.6	85.9	113.3	94.3	80.2
P07737	PFN1	86.2	72.9	78.6	99.5	164.2	152.8	95.2	77.3	67.4	70.8	69.6	146	79.4	112.1	140.3	87.6
P00441	SOD1	95.3	74.9	92.5	108.6	87.1	72	63.4	110.5	79	86.6	62.2	98.5	89.6	307.6	96.9	75.2
Q9H8L6	MMRN2	104.5	84.3	98.4	126	104.2	102.6	95.5	103.6	104.6	115.8	98.7	95.4	82	115.9	99.2	69.1
A0A0A0MRZ8	IGKV3D-11	88.1	79.2	104.3	121.8	112.3	107.4	115.4	89.2	88.1	88.4	65.6	132.9	155.7	78.1	93.7	79.7
Q9H4G4	GLIPR2	97.5	86.6	101.4	88.2	110.8	76.4	103.8	117.2	104.4	84.2	110.6	112.8	91.5	115.7	88.3	110.5
O00391	QSOX1	102.2	90.1	112.4	87	88.7	78.2	73.8	158.7	88.9	89	95.2	105.6	149.7	130	79.5	71
P61916	NPC2	93.8	86.3	82.4	98.8	94.4	106.1	96.8	76.2	86.4	96.4	77.4	78.7	89.6	232.5	122	82.1
A0A0B4J1V0	IGHV3-15	108.4	62.3	70.4	73.1	102.7	89.8	135	161.8	101	89.5	106.3	114.8	156.7	70.8	84.7	72.5
A1L4H1	SSC5D	78	93	124.9	108.7	80.5	108	97.6	90.5	102.2	122.9	100.3	90.7	79.2	101.4	106.4	115.8
P05451	REG1A	81	223.9	77.6	62.9	73.2	52.2	55.6	91.9	59.8	86.8	112.3	75.6	74.8	253.3	146	72.9
A0A0B4J1V6	IGHV3-73	114.6	94.5	72.6	83	83.4	115.2	82.3	126.4	85.7	115.2	81.5	148.6	132	83.8	96.9	84.5
A0A087WSZ0	IGKV1D-8	112.9	99.6	90.7	99.4	113.3	84.7	94.5	92	92.3	93.3	84.8	107.3	123.7	83.8	141.7	85.9
Q92496	CFHR4	77.6	106	77.1	127.3	64.9	108	82.6	99.4	80.8	116.8	122	59	141	121	140.5	75.8
Q8IZF2	ADGRF5	113.5	95.2	105	84.1	102.7	83.2	82.7	103.5	101.8	110.7	121.7	112.6	85	125.9	91.4	81
Q7RTS7	KRT74	82.2	84	96.1	83.6	132.4	118.7	95.1	80.9	101.9	112	111.7	95.1	121.5	86.1	100.3	98.3
Q9UBQ6	EXTL2	119.8	96.4	108.7	128.3	91.9	125.4	105.9	110.1	91.3	106.3	79	89.8	85	73.5	98.1	90.4
P24592	IGFBP6	80.2	72.9	86.1	71.8	87.6	70.3	72	77.3	72.5	98.3	81.7	80.5	84.8	385.9	99.7	78.5
Q9Y490	TLN1	96.3	95.6	85.1	102.2	144.7	102.7	83.9	87.4	86.5	109.3	86.9	127.3	107.8	79.4	113.7	91
P00326	ADH1C	132.9	141.2	126.9	115.6	136.5	70.7	48.9	93	100.6	111.7	106.1	110.8	116.5	83.1	58.1	47.6
Q16706	MAN2A1	102.4	102.1	107	117.2	100.9	106.9	98.1	95.4	101	97.1	86.3	101.8	105.8	84.9	93.4	99.5
Q96S96	PEBP4	84	61.7	91	80.3	93.1	61.2	81.4	71.6	85.5	54.6	100	86.3	58.1	485.2	54	52.2
P02788	LTF	86.7	71.3	74.9	87.3	99.9	83.8	111.6	160.6	91	87.6	77.9	91.8	97.5	219.3	75.9	82.9
Q04721	NOTCH2	98.5	80	113.5	97.1	161.8	94.3	84.7	73.8	82.5	83.3	89.9	98.3	160.8	105.2	101.8	74.4
Q12884	FAP	115.2	99.4	98.8	102.7	110.8	101.8	81.9	115.2	116.8	92.3	88.3	82.4	109.6	104.7	89.6	90.5
P07195	LDHB	86.2	80	101.8	83.4	102.5	82.6	140	128.6	72.9	70.6	74.4	118.9	142.8	146.1	88.5	80.8
P12109	COL6A1	119.5	88.7	133.8	102.1	103.4	123.9	80	78.8	94.4	100.2	81.7	90.5	92.1	116.2	91	103.7
P07477	PRSS1	100.1	96	113.9	93.8	94.3	89.8	96.5	98.2	89.3	95	104.5	104.3	88.4	120	103.9	112
P40227	CCT6A	101.2	101.7	110.9	90.3	129.8	85.6	106.6	77.2	99.1	72.8	93.9	98.5	171	98.4	79.6	83.4
Q96IY4	CPB2	90.8	89.2	120.3	100	121.6	72.8	62.5	127	116.4	76.7	61.9	122.5	151	116.5	98.2	62.8
P42357	HAL	145.3	150.4	118.5	101.9	132.8	61.9	74	95.4	104.4	99.5	79.5	98.7	111.9	94.9	72.7	58.3
P10153	RNASE2	77.7	82.7	72.9	56.7	89.8	68.4	105.4	85.9	57.2	80.1	65.6	71.6	105.6	422.7	83.8	73.9
P08195	SLC3A2	110.1	95	104.5	98.8	102.2	98.1	95.8	113.4	110.6	119.2	111.5	85.1	99.5	85.3	78.9	91.9
P01703	IGLV1-40	84.3	115.8	99.9	73.7	88.2	89	134	92.4	101.1	105.6	78	120.4	179.6	66.4	101.8	69.9
P12955	PEPD	102	88	90.2	105.8	103.4	111.7	108.5	110.1	119.2	103.1	111.3	108.1	92.1	80.5	83.3	82.7
P12273	PIP	77.1	89.7	95.6	141.4	74.8	171.4	135.6	92	100.1	104	89.3	80.5	78.4	99.9	71.1	98.9
Q96EE4	CCDC126	117.1	87.8	118.6	109.6	109.9	126.7	94.4	106.3	87.8	116	109.7	85.5	94.2	64.3	92.4	79.8
Q15517	CDSN	100.4	91.9	84	79.4	95	114.9	92.1	97.4	96.9	124.2	108.5	88.5	120.6	104.6	95.1	106.6
P61513	RPL37A	105.7	97.9	93.5	108.3	110	118.4	97.6	83.7	88.5	116.6	83.1	99	113.6	83.4	104.5	96.2
A0A0B4J1U3	IGLV1-36	78.3	210.5	88.1	92.2	92.8	128.8	94.3	96.8	73.3	82.4	90.8	98.4	136.7	62.5	134.3	39.7
P05019	IGF1	100.9	85.1	110.2	113.1	77.7	132.8	108	98.1	122.7	93.3	111.6	92.7	80.4	104.1	95	74.3
A0A0B4J2D9	IGKV1D-13	78.5	82.1	148.2	94.9	90.6	110	119.9	92.5	100	86.2	82.7	99.7	171.8	73.1	86.7	83.3
P06732	CKM	111.5	92.6	107.5	92.6	99.5	87.9	87.3	94.8	80.7	138.6	96.5	85.8	133.2	72.1	124.6	94.9
P09486	SPARC	115.9	71.9	157.6	75.2	101.2	147.5	111.1	148.4	67.4	92.8	75	80.1	74.6	73.8	110.1	97.4
P01706	IGLV2-11	64.6	167.4	91.9	56	57.9	51.5	88.6	86.1	87.5	58.8	126.4	72.9	286.4	111.7	126.1	66.1
P13987	CD59	62.8	50.5	68	71.1	64.6	66.8	68.3	64.6	62.6	62.9	57.7	49.8	56.3	668.9	74.3	50.8
P07307	ASGR2	85.6	62.9	86.6	71	101	64.8	93.2	129.6	70.3	73.4	77.2	77.3	70.9	320.1	145.3	70.9
P78417	GSTO1	92.3	89.5	89.1	111.3	94.6	99.1	91.7	141.7	99.1	87.5	100.5	108	98.7	89.1	105.5	102.3
Q86TY3	ARMH4	112.2	66.5	105.3	143.3	119.3	126.6	98.3	118.2	72.7	120	91.8	79.5	80.9	102.5	86.4	76.3
Q86U17	SERPINA11	145.2	105.9	58.8	73.2	101.7	77.3	77.7	106	113.5	111.5	116.7	97.6	161	89.4	83.8	80.6
Q76LX8	ADAMTS13	101.8	99.4	94	85	100.6	116.9	83.5	116.4	97.8	113.5	87.8	113.5	119.9	77.2	83.6	109.3
P12236	SLC25A6	118.2	113.8	88.5	129.8	87.5	105.5	71.6	129.1	86.6	111.2	103	97.7	120.7	89	68.8	79
A0A0G2JS06	IGLV5-39	109	64.1	55.5	47	52.9	88	108.5	130.2	74.9	105.6	49.8	143.7	480.2	33.3	35	22.4
Q02413	DSG1	97.3	84.4	100.9	75.8	111.3	103.1	97.2	89.3	81.3	107.4	81.4	79.3	143.9	130.9	121.3	95.1
P17931	LGALS3	107.5	91.9	85.9	108.6	103.9	101.7	84.4	94.7	109.8	100	86.7	92.9	103.3	107.6	105.2	115.8
P14209	CD99	128.7	121.7	103.7	106.4	102.8	102.9	144.4	121.3	79	85.7	65.3	77.9	95.2	133	37	95.1
P10599	TXN	87.3	67.7	89.7	112.8	112.3	115.7	111	102.2	88.3	85.9	110.4	93.1	121.5	140.3	86.7	75.3
O95479	H6PD	99.5	94.8	115.4	93.7	130.1	83.3	104.7	112.4	94.7	121.7	95.5	79.1	92.6	102	96	84.6
P01718	IGLV3-27	64.2	60.7	70.5	76.7	75.4	489.6	98.2	91.9	88.9	74.9	67.5	75.5	74.2	64.9	70.2	56.7
P11684	SCGB1A1	72.3	38.5	72.2	105.4	81.4	93.8	71.1	60.1	42.5	55.6	49	50.6	56.2	587	74.1	90.3
P10809	HSPD1	109.4	119.8	125.6	114.5	124.8	75	68.5	113.2	79.3	101.4	87.9	104.4	96.9	95.9	87.8	95.7
P15924	DSP	87.7	72.7	102	88.8	122.1	146.1	108.7	84.7	99.5	122.5	124.2	84.1	128.1	70.5	74	84.5
Q13093	PLA2G7	132	106.8	66.3	71.7	82.9	106.8	104.8	57.7	78	121.2	118.5	148.2	157	59.8	119.5	68.9
Q9Y365	STARD10	113.1	97.1	95	109.4	109.7	100.6	93.7	101.8	82.1	101.1	121	97.8	123.4	98.4	78	77.9
P01701	IGLV1-51	91.6	95.9	95.4	78.8	132.3	93.2	122.9	82.4	104.6	101.3	65.9	128.2	134.5	88.2	99.9	85.1
Q6E0U4	DMKN	79	48.3	73.6	91.5	84.1	93.6	93.9	69.3	39.4	80.2	63	50.9	91.5	442.2	126.2	73.4
Q10588	BST1	106.4	92	56.5	124.5	97.1	104.6	154.1	115.8	84.8	94.9	98.6	107.6	119	90.7	91.9	61.7
P01709	IGLV2-8	108.9	160.1	60	62.9	92.9	80.7	105.9	77.1	80.7	171.4	82.1	88.9	192.5	82	93.1	60.8
P08637	FCGR3A	89.3	108.1	87.5	115.3	90	111.1	104.8	103.8	99.7	82.9	95.3	133	103.6	79.8	93.8	102
P54802	NAGLU	110.5	94.8	120.7	106.3	99.4	123.7	87.7	112.4	103.8	145.2	114.6	73.9	83.3	64.1	85.3	74.4
P07942	LAMB1	114.5	71.8	104.8	108.6	93.3	139.1	108.4	95	116	103	92.9	85.9	86.8	89.3	96.2	94.2
P19022	CDH2	94.3	96.9	103.4	81.2	86.6	69.9	71	98.1	87.3	95.9	96.5	97.2	91.8	235.9	74.5	119.6
Q16853	AOC3	103.7	82.5	137.7	108	80.7	142	83.9	73.9	121.1	109.1	91.3	103.8	106.8	78.8	89	87.8
Q14697	GANAB	102.4	68.5	106.8	105.8	101.9	145.6	89	108.2	86.9	113	83.3	99.2	80.3	83.9	147.4	77.7
P05106	ITGB3	91.9	108	100.1	87.8	107.8	106	75.5	95.4	101.1	91	86.3	103	97.2	110.3	116.2	122.4
Q92859	NEO1	111.7	90.4	105.3	108.4	108.1	101.8	93.8	111.9	103	136.8	102.7	83.3	89.7	87.9	83.7	81.5
Q5SYB0	FRMPD1	138.2	73.8	146.3	116.5	89.9	133.4	81.3	95.9	90.2	112.5	68.8	78.5	108.4	98.7	80.3	87.4
Q9UJJ9	GNPTG	85.5	80.1	114.9	94.5	99.5	78.3	90.2	90.8	104.4	89.6	107.8	126.7	95.4	147.1	103.4	91.6
Q99784	OLFM1	112.2	76.3	117.3	103.7	95.5	87.3	113	102.4	98.7	96.4	99.4	81.5	118.6	132.7	89.3	75.6
P01705	IGLV2-23	69	150.3	98.8	49.7	71.5	96.1	69.7	109.7	101.7	52.5	124.2	50.8	332.3	69.7	103.5	50.3
Q12913	PTPRJ	108.9	104.9	97.7	94.8	109.3	116.5	106.7	108.3	79.8	112.6	105.7	108.5	97.6	73.2	84.8	90.8
P14923	JUP	91.8	98.5	87.5	92.2	80.8	91.4	120.9	87.6	90.1	93.8	134.4	95	147.9	91.1	109.6	87.3
Q9BYE9	CDHR2	88.4	113.7	67.1	70.2	101.5	124.4	80.9	128.6	104.2	113.9	182.5	98.1	87.2	76.5	95	67.7

A0A0C4DH24	IGKV6-21	83.9	115.3	154.7	74	82	69.8	91.5	101.2	92.5	91.6	123.9	129.5	111.9	88	89.8	100.4
Q8N1N4	KRT78	75.3	92	115	76.7	134.6	109.8	115.9	89.8	101.2	95.4	136.4	89.6	115.2	65.1	99.1	89
P04424	ASL	103	100	90.3	99.5	89.5	117.7	81.3	215.4	95.9	102.8	122.5	89.3	71.4	81.4	80.3	59.6
P01344	IGF2	82.4	85.6	95	101.5	84.2	72	85.8	103.6	78.5	80.9	89.7	81.9	99	274.1	100	85.8
P26038	MSN	98	83.8	87.1	94.2	125.8	103.7	92.5	109.2	100.3	97.2	97.3	119.4	99.3	116	76.4	99.7
P62491	RAB11A	79.5	101	92.5	88.8	94.4	97.8	135.2	104.2	100.6	58.7	90.4	139.9	135.9	94.1	110.6	76.4
P00505	GOT2	130.5	98.1	115	76.2	120.1	89.1	110.5	112.9	95.5	109.3	103.8	96.2	88.5	79.1	103.4	71.6
P11362	FGFR1	109.9	81.8	126.1	104.9	97	124.6	114.5	107.1	96.8	109.9	86.1	87.2	86.5	103.5	85.9	78.1
P25774	CTSS	88.8	115.9	103.3	107.3	93.6	85.5	106.4	97.8	117.8	109.4	125	79.9	89.1	84.8	89.7	105.6
P14314	PRKCSH	88.6	76	100.1	101.6	112	99.2	108.2	116.3	77.1	95.8	102.8	111.3	90.8	143.1	87.6	89.6
P16930	FAH	61.3	116.6	75.7	88.7	66.6	68.5	82.7	282.7	84.9	138.2	109.9	116.5	97.4	60.1	85.9	64.3
A0A075B6K0	IGLV3-16	189.9	56.5	69.5	208.3	74.2	181.4	74.4	82.5	85	73.2	58.5	69.5	150.5	63.6	82.5	80.4
O95497	VNN1	105.6	110.9	78.8	121.6	115.6	85.9	115.8	89.8	78.1	90.5	128.8	138.1	69.4	66.3	111.3	93.4
Q5T619	ZNF648	144.1	127.4	138.9	182.2	70.9	74.6	54	120	60.3	106.1	78.2	105.4	59.2	40.5	107.1	131
P20774	OGN	72.6	59.8	66.8	82.3	105.1	79.4	76.1	74.4	44.8	80.1	65.1	61.7	72.9	498.8	84.4	75.6
A0A0C4DH36	IGHV3-38	102	92.9	87.6	83.9	99.5	109.1	86	95.9	65.9	79.3	99.1	142.4	191.6	80.4	109.2	75.3
Q9NPR2	SEMA4B	108	83.2	93.9	92.2	91.2	94.8	98.6	91.3	108.7	99.2	92.9	127.7	72.5	176.4	82.5	86.8
Q8WZ42	TTN	99.2	96.7	94.6	116.4	95.8	50.2	73.4	88.2	142.6	52.2	26.1	221.2	258.9	46.6	71.4	66.6
P23470	PTPRG	85.1	89	116.6	116.7	85.3	60	120.7	112.9	83.4	92.7	108	78.6	97.6	89.4	150.5	113.5
P05556	ITGB1	98.1	104.2	115.1	86.3	142.4	87.6	97.9	101.3	77	104.5	75.2	105.9	75.1	90	125.3	114
A0A075B6I4	IGLV10-54	87.3	126.7	51.1	46.4	94.3	115	174.7	134.6	60.5	250.6	137.5	154.4	24.6	58.2	71.3	12.9
Q6P3W7	SCYL2	141	49	96.5	146.5	145.9	92.6	108.7	111.4	85.8	99.4	62.9	128.1	146.8	53.1	78.8	53.6
P07988	SFTPB	67.4	87.2	62.1	101.6	66.6	48.6	207.2	75.3	67.6	76.5	113.9	73	100.5	87	93	272.4
Q16851	UGP2	71.8	91.4	103.8	91.4	107.1	95.2	65.3	170	98.3	86.6	103.9	110.2	91.3	100.7	87.6	125.3
Q6ZN30	BNC2	105.3	99.4	113.2	103.6	99.9	98.1	106.1	104.3	102.2	107.7	109.5	88.9	81.3	95.1	91.5	93.9
P01160	NPPA	54.5	55.4	67.7	46	68.1	77.4	53.2	60.6	32.5	120.1	37.8	61	105.5	476.9	201	82.2
P01854	IGHE	70.8	82	53.2	35.5	41.5	85.4	102	52.4	45	51	55.5	73.9	26.6	129.3	640.6	55.4
P06703	S100A6	135.8	101.6	146.7	127.7	100.2	75	74.9	103.2	64.1	105.3	87	108	73	56.7	122.7	118.1
O00592	PODXL	104	100.9	91.7	91.2	98	97.3	97.3	91.5	84.8	113.6	101.8	97	93.6	88.8	122.8	125.8
A0A0A0MT89	IGKJ1	70.9	54.4	184.3	107.5	118.4	248.2	93.8	64	53.6	56	34	136.5	187.3	67.1	83	40.9
P62328	TMSB4X	46.7	33.9	43	48.6	139.1	67.1	68.5	49.5	21.1	46.1	54	131.9	53.6	643.6	87.5	65.7
P04080	CSTB	93.1	91.5	74.8	115.8	106.7	122.2	104.3	80.6	84	79.4	71.5	77.6	107.7	201.5	111.6	77.9
P29401	TKT	84.4	91	109.5	79.3	86.6	115.3	100.8	126	97.9	91.8	95.6	98.7	108.9	129.6	101.5	82.9
O60333	KIF1B	102.6	66.6	88.9	74.1	215	227	124.2	77.5	64.3	64.2	57.1	126.9	105.2	70.8	73.8	61.8
A0A0B4J1V7	IGHV7-81	104.3	55.8	105	101.9	129.4	85	144	143.3	155.5	90.6	35.4	125.5	123.9	69.2	92.6	38.6
P55265	ADAR	109.7	83.7	112.9	97.7	106.9	115.9	110.4	103.8	90.2	103.4	108.3	76.2	91	94.7	101.1	94.2
O43896	KIF1C	107.5	47.9	86.2	85.5	77	73.4	461.6	84.9	50	71.4	42.4	104.8	145.9	33.2	97.9	30.4
P35555	FBN1	104.1	76.4	105.8	79	82.2	103.1	73.8	63.4	91.7	86	74.8	188.4	110.7	151.2	117.5	91.7
P04432	IGKV1D-39	76.1	58.7	78.6	78	58.3	600.6	60.4	72.2	121.6	56.4	52.3	71.1	58.8	53.3	53.8	49.8
O43505	B4GAT1	124.3	84.7	102.3	114.3	99.8	112.7	122	107.4	109.6	104.3	79.3	98.9	83.6	86.6	90	80.3
Q12965	MYO1E	73.1	153.7	99	81.8	101.3	130.2	126.8	105.1	177.2	88.2	79.7	62	92.9	93.7	91.1	44.3
P30046	DDT	68.2	63.8	102.3	76.8	55.1	71.3	106.5	298.2	79.6	71.8	92.2	109.9	95.9	131.5	107.5	69.4
Q8N149	LILRA2	124.5	130.2	89.4	99.3	89.4	134.4	113.1	47.7	97.7	67.1	84	138.7	84.1	102.2	115.1	83.1
A0A075B6H7	IGKV3-7	106.8	90.1	88.9	160.4	84.3	117.8	100.9	117.2	110.8	98.3	86.5	130.5	113.9	50.3	90.5	52.7
Q8IU80	TMPRSS6	83.2	82.1	77.2	171.6	74.6	147.1	179	66.4	71.1	65	151	126.5	64.4	100.5	61.4	78.9
P02511	CRYAB	99.7	89.1	88.5	99.8	109.8	119.2	75.7	95.8	103.3	154.7	101.7	87.2	129.2	79.8	96.7	69.9
A6NKL6	TMEM200C	53	272	42.8	80.6	68.7	56	68.8	46.8	46	55.2	192.4	68.8	237.4	26.7	255.1	29.9
Q9Y646	CPQ	69.7	103.1	134.9	80.1	80.7	130.3	60.3	118.9	104.7	127.3	111.3	99.1	92.7	94.4	97.4	94.9
O75023	LILRB5	90	101.6	97.3	84.2	132.8	162.7	91.1	96.8	98.2	123.2	83.2	109.7	105.1	64.5	96.7	62.8
P15151	PVR	110.3	70.1	110.7	86.1	82.5	99.9	102.1	134.9	106.6	117.2	115.5	92.6	95.3	85.3	100.6	90.4
P01704	IGLV2-14	59.5	113.3	93	52.8	68.5	71.6	70.7	96.9	77.6	66.5	339.1	66.9	160.7	115.3	98.3	49.1
A2RUR9	CCDC144A	146.1	84.2	68.9	73.9	139.4	103.2	55	163.2	64.5	47.7	80.2	172	286.9	47.9	35.7	31.3
O95998	IL18BP	95.9	102.9	90.3	106.9	113.5	83.5	113.6	97.9	83.3	78.6	97	95.8	90.1	153.8	112	85.1
O95477	ABCA1	83.1	79.8	112.7	161.9	91.9	94.4	81.5	152.4	71.1	83.5	62.7	143.8	92.8	86.6	87.7	114.3
Q13508	ART3	81.2	51.4	122.4	77.3	92.3	79.2	100.6	83.6	69.9	86.1	108	84.8	71.6	292.1	131.8	67.7
Q9H4B0	OSGEPL1	105.3	78.7	93.7	103.4	90.7	134.7	230.5	70.3	66.6	100.2	69.3	98.7	178.5	45.4	81.3	52.8
Q12841	FSTL1	92.2	75.3	108.9	88.9	90	118.6	98.7	106.8	100.8	110.1	87.3	91.2	90	142.9	92.3	106.2
Q8NI99	ANGPTL6	101.2	110.4	92	86.4	104.2	106.5	105	139.2	93.8	114.2	96.8	96.7	73.9	91.2	88.4	100
P30101	PDIA3	95.5	103.5	89.6	72.4	97.4	77	90.7	120.1	101	95.8	98.4	112.7	98.2	125.8	112.4	109.5
Q9H4A9	DPEP2	102.6	76.2	97.3	141.3	130	154.3	117.6	94.4	84.5	101.7	122	80.8	82.7	76.3	85	53.6
Q92835	INPP5D	103.7	109.6	164.5	40.6	33.6	123.4	28	77.3	46.3	33.6	63.4	68.2	297.8	22.7	361.2	26.1
Q5U651	RASIP1	124	130.7	96.2	97.7	110.5	107.7	73.9	113.6	96.8	102.6	111.8	83.8	117.2	62.7	83.2	87.7
Q6UXH0	ANGPTL8	123.5	114.8	83.1	93.1	103	110.4	126.3	113.8	81.2	114.6	85	116.9	113.9	72.2	63.2	85.1
Q8IZU0	FAM9B	158.9	127.9	142.3	187.6	71.5	83.7	59	109.4	55.4	90.9	59.5	101.5	64.6	32.3	114	141.4
P55083	MFAP4	107.2	90	98.9	97.8	75.1	104.8	77.5	109.9	82.9	97.5	90.4	128.5	111.6	100.3	146	81.4
P39060	COL18A1	123	74.1	100.7	118.6	78.1	83	81.7	150	102	89.5	104.8	109.7	87.4	89.6	89	118.7
Q8N387	MUC15	46.4	54.2	51.8	61	41	825.7	52.6	54.9	49.9	56.5	41.2	92.1	45.9	31.9	50.5	44.5
P82094	TMF1	113	99.8	122.8	113.1	128.8	145.7	105.7	135.8	84.7	98.5	89.2	66.1	83.8	66.2	76.7	70.2
P40189	IL6ST	114	85.9	118.4	94.9	90.7	111.3	102.8	100.5	104	100.8	109.3	92.8	122.2	104.8	80.1	67.3
Q5T749	KPRP	69.7	90.2	88	84.5	116.8	149	115.8	77.8	137	125.7	124.9	84.7	92.6	70.7	91.9	80.6
P48741	HSPA7	96.6	87.3	120.9	117.3	92.8	92.3	113.1	97.5	73.2	105	113	98.9	112.6	101.4	81.4	96.7
Q12907	LMAN2	93.3	100.1	110.2	103.8	124.1	106.2	107.8	125	85.3	118.4	93.5	65.4	97.5	95.2	101.7	72.6
Q6NSI4	RADX	80.4	61	71.1	78.2	140.9	92.1	143.7	120.8	61.3	82.2	47.8	102	265.7	128.5	61.3	63
Q9UHN6	CEMIP2	97.9	111.7	86.4	90.8	207	92.1	99.1	105.2	106.7	67.8	83	79	88.6	87	88.7	109
Q14956	GPNMB	94.7	79.9	211.8	60.1	84.2	90.2	83.4	102.5	132.2	96.3	107.8	124.6	99.4	73.8	63.5	95.6
B3KU38	QCJ-SCHIP	95.3	91	100.8	96.7	95.7	98.8	103.7	113	87.5	109.4	122.2	101.4	101.2	73.7	100	109.6
P33981	TTK	120.5	128	72.1	70.7	87.8	86.8	67.8	84.9	102.8	116.6	117.4	111.8	151	69	89.5	123.3
Q8TAQ9	SUN3	95.2	112.3	114	131.4	96.1	107.9	124.2	105.1	116.4	102	101.8	89.2	68.6	63.8	76.5	95.5
P49221	TGM4	73.1	108.4	84.7	104	111.8	99	139.3	99.3	92.6	71.2	64.2	146.7	176.8	84.6	78.2	66.1
Q9H115	NAPB	124.8	75.6	86.1	117.2	79.3	67.6	210.6	74.3	45.4	79.2	80.7	149.5	154.8	94.1	128.6	32.2
