# Supplementary material for: Therapeutic effects on the development of heart failure with preserved ejection fraction by the sodium-glucose cotransporter 2 inhibitor dapagliflozin in type 2 diabetes
Source: Diabetol Metab Syndr. 2023 Jun 29;15:141. doi: 10.1186/s13098-023-01116-8 (PMC10308685; doi:10.1186/s13098-023-01116-8)
Supplement: Supplementary file 8 — Additional file 8: Table S3. Metabolomics data for serum metabolite profiles across loading samples (1 QC group and 4 experimental groups). [file 13098_2023_1116_MOESM8_ESM.pdf]

Table S3. Metabolomic data for serum metabolite profiles across loading samples (QC group & 4 experimental groups).

Alignment ID	Average Rt (min)	Average RI	Quant mass	Metabolite name	QC-2	QC-3	QC-4	QC-5	DAPA-1	DAPA-2	DAPA-3	DAPA-4	DAPA-5	DM-1	DM-2	DM-3	DM-4	DM-5	DM-HF-1	DM-HF-2	DM-HF-3	DM-HF-4	DM-HF-5	NC-1	NC-2	NC-3	NC-4	NC-5
0	5.187	262163	174.116	Ethylamine	1.1083	1.12716	1.1967	0.9906	1.2418	1.2466	1.14551	1.0696	0.9901	1.2708	1.1326	1.2852	1.1533	0.9767	1.2467	1.20701	1.1453	1.2687	1.2261	1.1341	1.20509	1.1857	1.1844	1.2508
3	5.264	262167	171.1	Methylamine	0.6966	0.74883	0.7718	0.688	0.9469	0.5815	0.86385	0.5377	0.7992	0.8697	0.869	0.688	0.8014	0.787	0.7519	1.79232	1.4152	0.81783	0.7664	0.5829	0.37079	0.6384	0.6048	0.37
5	5.407	262176	146.067	Hydroxylami	0.0011	0.00085	0.0009	0.0009	0.001	0.0009	0.00091	0.0009	0.0009	0.0009	0.0009	0.0009	0.001	0.001	0.0007	0.00077	0.0009	0.00097	0.0009	0.0009	0.00108	0.001	0.001	0.001
9	5.498	262181	89.0875	Methyl 2-(2-	0.0042	0.00469	0.0049	0.0049	0.0045	0.0033	0.00507	0.0045	0.0069	0.0059	0.0078	0.0033	0.0059	0.0085	0.0028	0.00224	0.0045	0.00412	0.0029	0.0029	0.00586	0.0023	0.003	0.0039
10	5.519	262182	221.1	Boric acid	0.0056	0.00602	0.0067	0.0062	0.0083	0.0043	0.00473	0.0068	0.0071	0.0029	0.0066	0.0026	0.0063	0.0073	0.0031	0.0043	0.0025	0.00371	0.0027	0.0027	0.00218	0.003	0.0022	0.0029
12	5.627	262189	160.047	Dimethylglyc	0.0009	0.00089	0.0009	0.0009	0.001	0.0009	0.00092	0.0002	0.0009	0.0006	0.0007	0.0005	0.0007	0.0008	0.0007	0.00214	0.0007	0.00083	0.0009	0.0006	0.00057	0.0008	0.0006	0.0008
14	5.768	262197	117.1	Butane-2,3-d	0.0012	0.00125	0.0012	0.0012	0.0008	0.0012	0.00043	0.0011	0.0004	0.0005	0.0004	0.0005	0.0126	0.0005	0.0005	0.00062	0.0012	0.00083	0.0016	0.0039	0.00065	0.0004	0.0004	0.0006
15	5.84	262201	89.0571	2-ketobutyric	0.0013	0.00142	0.0017	0.0014	0.0024	0.0017	0.00227	0.0023	0.0022	0.0014	0.0021	0.0016	0.002	0.0021	0.0017	0.00027	0.0002	0.00165	0.0015	0.001	0.00117	0.0012	0.0012	0.0013
18	5.91	262205	115.1	Diacetone alc	0.0039	0.00437	0.0042	0.0042	0.0048	0.0048	0.00429	0.005	0.0045	0.0043	0.0047	0.0046	0.0047	0.0048	0.0043	0.00412	0.0042	0.00456	0.0041	0.0038	0.00422	0.0039	0.0041	0.0044
20	6.06	262214	104.046	Methoxyami	0.0019	0.00242	0.0014	0.0016	0.0013	0.0009	0.00111	0.0021	0.0022	0.0028	0.0014	0.0018	0.0017	0.0025	0.0013	0.00081	0.0017	0.00107	0.0009	0.0028	0.00439	0.0024	0.0021	0.0018
22	6.14	262219	114.093	1-propylamir	0.0028	0.00299	0.003	0.0029	0.0032	0.0033	0.00297	0.0035	0.0031	0.0027	0.003	0.0029	0.0031	0.0031	0.0031	0.00301	0.0028	0.00295	0.0029	0.0026	0.0027	0.0027	0.0029	0.0028
23	6.155	262220	147.1	Ethanolamine	0.0231	0.02605	0.027	0.0271	0.0406	0.0255	0.02476	0.0413	0.0375	0.0269	0.043	0.0286	0.0363	0.0486	0.0263	0.03624	0.0245	0.02676	0.0254	0.0184	0.02166	0.0201	0.0236	0.0248
24	6.182	262221	128.1	Cyclohexylai	0.0011	0.00132	0.0013	0.0012	0.0015	0.0013	0.0013	0.0014	0.0012	0.0014	0.0016	0.0014	0.0014	0.0015	0.0013	0.00123	0.0012	0.00133	0.0013	0.0013	0.00124	0.0012	0.0012	0.0013
25	6.23	262224	136.06	2-picolinic ac	0.0014	0.00158	0.0017	0.0017	0.0025	0.0016	0.00156	0.0027	0.0025	0.0015	0.0026	0.0015	0.0018	0.0032	0.0014	0.00387	0.0014	0.00152	0.0015	0.0011	0.00132	0.0013	0.0015	0.0015
27	6.317	262229	98.0765	N-methylglut	0.001	0.00112	0.0012	0.0011	0.0019	0.0012	0.00122	0.0015	0.0019	0.0011	0.0017	0.001	0.0017	0.0019	0.0027	0.00129	0.0012	0.00119	0.0018	0.0008	0.00121	0.0008	0.0009	0.001
28	6.343	262231	170.124	Epsilon-capr	0.001	0.00104	0.0014	0.001	0.0015	0.001	0.00097	0.0012	0.0013	0.0013	0.0014	0.001	0.0014	0.0017	0.0011	0.00115	0.0012	0.0013	0.0011	0.0011	0.00122	0.001	0.0013	0.001
29	6.372	262232	318.084	Kynurenic ac	0.0015	0.00184	0.0016	0.0017	0.0024	0.0015	0.00157	0.0023	0.0022	0.0018	0.0021	0.0018	0.0021	0.0024	0.0017	0.00143	0.0015	0.00155	0.0015	0.0013	0.00162	0.0014	0.0015	0.0016
30	6.385	262233	152.1	3-pyridinol	0.0412	0.04522	0.0469	0.0429	0.0438	0.041	0.04176	0.0458	0.0466	0.0526	0.0608	0.0463	0.0428	0.0625	0.0417	0.0429	0.0496	0.04274	0.0363	0.0339	0.04257	0.0354	0.0362	0.0408
33	6.539	262242	174.084	Pyruvic acid	0.014	0.01586	0.0156	0.0144	0.0205	0.019	0.00765	0.0215	0.0083	0.0151	0.017	0.0155	0.0163	0.0172	0.0293	0.01	0.0188	0.01471	0.0121	0.0133	0.01139	0.0124	0.0115	0.0151
35	6.597	262245	127.1	Homoglutam	0.0265	0.02881	0.0245	0.0243	0.0282	0.0242	0.02261	0.0313	0.0285	0.0265	0.0354	0.0271	0.032	0.0342	0.0294	0.01543	0.0233	0.02433	0.0286	0.0287	0.03079	0.029	0.0291	0.025
36	6.597	262245	127.1	2-ketoadipic	0.026	0.02882	0.0246	0.0244	0.0279	0.0229	0.02262	0.0313	0.0285	0.0265	0.0354	0.0261	0.032	0.0342	0.0279	0.01543	0.0234	0.02433	0.0277	0.0281	0.03086	0.0285	0.0284	0.025
39	6.692	262251	147.1	L-lactic acid	1.2873	1.29828	1.3098	1.2587	1.14	1.4971	0.90807	1.4713	0.9083	1.3835	1.3406	2.0442	0.9834	1.3181	1.9758	0.45426	0.9969	1.34487	1.1986	1.2954	1.5563	0.9687	1.7152	1.3598
40	6.774	262256	147.1	Carbamic aci	0.027	0.03298	0.0257	0.024	0.0293	0.0191	0.03036	0.015	0.0296	0.0419	0.0329	0.0218	0.0344	0.0328	0.0186	0.04493	0.0434	0.02699	0.0221	0.0232	0.03005	0.0207	0.0215	0.0207
43	6.864	262261	164.99	Benzylalcoho	0.0051	0.00397	0.01	0.0056	0.0037	0.0101	0.01052	2E-06	0.0008	0.0053	4E-05	0.0017	3E-05	6E-05	0.0035	0.00815	0.0049	0.00783	0.0092	5E-06	1.6E-05	5E-06	0.0015	0.005
45	6.962	262267	177.1	Glycolic acid	0.0008	0.001	0.0009	0.0008	0.0018	0.001	0.00072	0.0011	0.0009	0.0008	0.001	0.0009	0.0007	0.0011	0.0008	0.00086	0.0007	0.00076	0.0009	0.0007	0.00092	0.0007	0.0007	0.0008
46	6.987	262268	258.079	Citraconic ac	0.0013	0.00168	0.0014	0.0013	0.0018	0.0013	0.00128	0.0019	0.0017	0.0014	0.0013	0.0013	0.0016	0.0016	0.0015	0.00161	0.0011	0.00147	0.0013	0.0013	0.00153	0.0015	0.0016	0.0016
47	7.109	262275	311.1	4-aminophen	0.0001	0.00017	0.0002	0.0002	5E-05	8E-05	5.4E-05	0.0001	8E-05	3E-05	8E-05	4E-05	9E-05	0.0001	3E-05	7.2E-05	4E-05	7.5E-05	6E-05	2E-05	6.6E-05	4E-05	5E-05	6E-05
48	7.148	262278	147.1	2-oxo-propar	0.0049	0.00536	0.0053	0.0048	0.008	0.0067	0.00489	0.0074	0.005	0.0033	0.0036	0.0023	0.0059	0.0039	0.002	0.02183	0.01	0.00688	0.009	0.0017	0.00422	0.0017	0.0021	0.0093
51	7.26	262284	173.1	4-methylvale	0.0006	0.00067	0.0006	0.0006	0.0007	0.0006	0.00053	0.0006	0.0006	0.0007	0.0007	0.0006	0.0005	0.0007	0.0006	0.00073	0.0006	0.00057	0.0005	0.0007	0.00083	0.0007	0.0006	0.0007
53	7.341	262289	154.02	Maleimide	0.0004	0.00046	0.0004	0.0004	0.0005	0.0004	0.0005	0.0004	0.0006	0.0008	0.001	0.0005	0.0004	0.0012	0.0004	0.00121	0.0004	0.00035	0.0003	0.0003	0.00036	0.0003	0.0005	0.0005
55	7.404	262293	116.1	L-alanine	0.2671	0.29532	0.2905	0.2697	0.3563	0.4148	0.23534	0.4551	0.2504	0.2385	0.2577	0.2639	0.1915	0.2586	0.6982	0.24882	0.2887	0.3124	0.3411	0.3434	0.1768	0.2705	0.2144	0.3328
56	7.457	262296	187.138	Octanol	0.0003	0.00028	0.0003	0.0003	0.0003	0.0003	0.0003	0.0003	0.0003	0.0004	0.0003	0.0003	0.0003	0.0003	0.0003	0.00032	0.0002	0.0003	0.0003	0.0003	0.00028	0.0003	0.0003	0.0003
57	7.51	262299	89.1	2-ketoisovale	0.0013	0.00143	0.0013	0.0012	0.0012	0.0019	0.00132	0.0018	0.0013	0.0017	0.0017	0.002	0.0011	0.0018	0.0025	0.00104	0.0013	0.0015	0.0012	0.0012	0.00094	0.0013	0.0008	0.001
60	7.688	262309	252.963	Vanillic acid	0.0001	0.00017	0.0001	0.0001	0.0002	0.0001	0.00014	0.0001	0.0001	0.0001	0.0001	0.0001	1E-04	0.0001	0.0001	0.00023	0.0001	0.00016	0.0002	0.0002	0.00014	0.0002	0.0002	0.0002
63	7.808	262316	131.1	2-hydroxybu	0.0417	0.04577	0.0455	0.0424	0.0517	0.0529	0.02832	0.06	0.0298	0.0555	0.0598	0.0885	0.0373	0.0608	0.0742	0.02133	0.0359	0.06687	0.0749	0.0237	0.02814	0.0208	0.0318	0.0256
67	7.945	262324	202	Erythrose	0.0002	0.0002	0.0002	0.0002	0.0002	0.0002	0.00016	0.0003	0.0002	0.0002	0.0003	0.0003	0.0002	0.0003	0.0004	0.00016	0.0001	0.00021	0.0002	0.0002	0.00013	0.0002	0.0002	0.0001
68	7.995	262327	116.1	Adrenaline	0.0009	0.00099	0.001	0.001	0.0019	0.0017	0.00144	0.0022	0.0018	0.0008	0.0015	0.0007	0.0011	0.0016	0.0019	0.00239	0.001	0.00111	0.0014	0.0008	0.00056	0.0011	0.0074	0.0007
72	8.122	262335	177	3-hydroxypro	0.0002	0.00026	0.0002	0.0002	0.0003	0.0002	0.00034	0.0002	0.0003	0.0014	0.0003	0.0002	0.0002	0.0003	0.0003	0.00017	0.0002	0.00034	0.0004	0.0001	7.8E-05	0.0001	0.0002	0.0005
74	8.173	262338	234.108	Piperidone	0.0009	0.00112	0.0011	0.001	0.0008	0.0006	0.00089	0.0008	0.001	0.0018	0.002	0.001	0.0009	0.0022	0.0008	0.00117	0.0016	0.00091	0.0007	0.0007	0.00078	0.0008	0.0005	0.0007
75	8.325	262347	165.1	P-cresol	0.0024	0.00291	0.0035	0.0032	0.0018	0.0016	0.00374	0.0017	0.0052	0.0016	0.0027	0.0006	0.0024	0.0032	0.0011	0.04417	0.0016	0.00343	0.0014	0.0004	0.0019	0.0016	0.0009	0.0004
77	8.38	262350	117.1	(r)-3-hydroxy	0.0102	0.01114	0.0112	0.0106	0.0085	0.0133	0.00965	0.0143	0.0101	0.0082	0.0089	0.0121	0.0075	0.0093	0.0238	0.00802	0.0081	0.0181	0.0332	0.008	0.00435	0.0065	0.0045	0.0052
78	8.427	262352	145.104	2-hydroxy-3-	0.0059	0.00686	0.0066	0.0063	0.0067	0.0071	0.00501	0.0081	0.0053	0.0049	0.0054	0.0112	0.0073	0.0055	0.0055	0.01166	0.0047	0.0078	0.007	0.0059	0.00841	0.0058	0.0048	0.0061
80	8.544	262359	187.114	2-hydroxyval	0.0002	0.0002	0.0002	0.0002	0.0002	9E-06	0.00018	0.0002	8E-06	0.0002	0.0002	3E-06	0.0002	2E-05	0.0002	0.00018	0.0002	3.7E-06	0.0002	0.0002	3.9E-06	0.0002	0.0002	0.0002
81	8.583	262362	130.1	2-aminoisobu	0.0088	0.01004	0.0099	0.0094	0.0152	0.0101	0.00901	0.012	0.01	0.0106	0.0126	0.0139	0.0057	0.0131	0.0302	0.00592	0.011	0.00847	0.015	0.0109	0.00469	0.0062	0.0044	0.0052
88	8.791	262374	151	Phenol	0.0003	0.00032	7E-06	0.0003	0.0002	1E-05	0.00026	0.0003	0.0003	4E-06	0.0003	0.0003	0.0003	0.0004	0.0002	0.00022	1E-05	0.00021	0.0002	0.0002	0.00021	0.0002	0.0002	0.0002
91	8.917	262381	210.019	Tartaric acid	0.0002	0.00014	0.0001	0.0001	0.0001	0.0001	0.00015	0.0002	0.0002	0.0001	0.0001	0.0001	0.0002	0.0002	0.0001	0.00019	0.0001	0.00014	0.0001	1E-04	0.00013	0.0001	0.0001	0.0001
94	9.055	262389	200.087	3-methyl-2-o	0.0004	0.00039	0.0004	0.0004	0.0004	0.0004	0.00036	0.0005	0.0004	0.0006	0.0006	0.0006	0.0002	0.0007	0.0009	0.00034	0.0003	0.00049	0.0003	0.0003	0.00018	0.0005	0.0003	0.0002
97	9.242	262400	102.092	3-aminoisobu	0.001	0.00096	0.0008	0.0008	0.0007	0.0011	0.00104	0.0013	0.0012	0.0009	0.0008	0.0014	0.0005	0.0008	0.0008	0.00434	0.0035	0.00074	0.0019	0.0014	0.00048	0.0015	0.0006	0.0008
99	9.306	262404	200.1	2-ketoisocap	0.0017	0.00198	0.0019	0.0019	0.002	0.0023	0.00138	0.0026	0.0014	0.0027	0.0031	0.003	0.0016	0.0033	0.0033	0.00125	0.0016	0.00242	0.0014	0.0012	0.00087	0.002	0.0011	0.001
100	9.326	262405	218.1	L-tryptophan	0.0187	0.02124	0.0225	0.0213	0.0274	0.0321	0.02487	0.0403	0.0284	0.0247	0.036	0.024	0.0158	0.0344	0.0386	0.03711	0.031	0.02889	0.0224	0.0226	0.00974	0.0252	0.0082	0.0192
101	9.326	262405	144.112	L-valine	0.0989	0.1115	0.1183	0.1111	0.1414	0.1701	0.12748	0.2073	0.146	0.1296	0.1857	0.126	0.0819	0.1771	0.2009	0.19295	0.161	0.15023	0.1174	0.1201	0.05089	0.1347	0.0441	0.101
102	9.361	262407	162.1	Butyraldehyd	0.0007	0.00072	0.0007	0.0007	0.0003	0.0008	0.00048	0.0009	0.0005	0.0014	0.0014	0.0014	0.0012	0.0013	0.0002	0.00058	0.003	0.00052	0.0007	0.0003	0.00063	0.0001	0.0001	0.0006
104	9.47	262414	117.05	Digitoxose	0.0034	0.00368	0.0036	0.0033	0.0034	0.0022	0.00316	0.0019	0.0031	0.0032	0.0028	0.0022	0.0031	0.0027	0.0029	0.01002	0.0056	0.00327	0.0022	0.0033	0.00395	0.0035	0.003	0.0023
108	9.622	323122	116.1	L-alanine-ala	0.0034	0.00343	0.0035	0.0036	0.0036	0.0035	0.00337	0.0048	0.0042	0.0029	0.0057	0.0031	0.0043	0.0053	0.0045	0.00385	0.0042	0.00336	0.0035	0.003	0.00239	0.0036	0.0098	0.0028
109	9.695	323127	277.139	Glutaric acid	0.0004	0.00055	0.0005	0.0004	0.0003	0.0003	0.00039	0.0004	0.0004	0.0006	0.0006	0.0005	0.0004	0.0008	0.0003	0.0006	0.0008	0.00038	0.0003	0.0003	0.00039	0.0003	0.0002	0.0003
117	9.985	323144	147.1	Urea	1.5863	1.70227	1.6857	1.6893	1.9674	1.1665	1.84088	1.2651	1.9348	1.8206	1.9285	1.3937	1.7059	1.9975	1.5226	1.50171	2.2204	1.65269	1.5991	1.2572	0.79977	1.3156	1.2734	0.7977
121	10.155	323154	100.1	1-methylhyda	0.001	0.00115	0.0008	0.0012	0.0018	0.001	0.00083	0.0008	0.001	0.0024	0.0029	0.0017	0.001	0.0027	0.0009	0.09258	0.0543	0.00174	0.001	0.0007	0.00115	0.0007	0.0012	0.0015
123	10.198	323156	174.104	N-acetylputre	0.0014	0.00171	0.0017	0.0018	0.0022	0.0017	0.0017	0.0021	0.002	0.0014	0.002	0.0015	0.0012	0.002	0.0029	0.01515	0.002	0.00179	0.0017	0.002	0.00118	0.0014	0.0008	0.0011
124	10.219	323158	201.108	Octanoate ra	0.0023	0.00273	0.0026	0.0027	0.0027	0.0029	0.00265	0.003	0.0029	0.0024	0.0027	0.0025	0.0027	0.0028	0.0026	0.00181	0.0021	0.00247	0.0026	0.0036	0.00247	0.0021	0.0023	0.0026
126	10.29	323162	158.142	Leucine	0.0617	0.07409	0.0695	0.0704	0.0952	0.1265	0.06716	0.153	0.0796	0.0843	0.1039	0.0761	0.0424	0.106	0.1221	0.0851	0.0844	0.09802	0.0704	0.0803	0.03076	0.109	0.0256	0.0591
128	10.316	323163	147.1	Glycerol	0.0721	0.07318	0.073	0.0726	0.0942	0.0827	0.05007	0.0913	0.0547	0.0704	0.0767	0.0749	0.0579	0.0785	0.0684	0.09352	0.0464	0.08289	0.0838	0.0632	0.04921	0.0577	0.1662	0.0464
130	10.435	323170	180	Nicotinic aci	0.0002	0.00021	0.0002	0.0002	0.0006	0.0003	0.00025	0.0006	0.0005	0.0002	0.0004	0.0002	0.0004	0.0006	0.0002	0.00028	0.0002	0.00025	0.0003	0.0001	0.00019	9E-05	0.0002	0.0002
131	10.463	323172	200.096	2,3-dihydro-8	0.0004	0.00048	0.0004	0.0004	0.0004	0.0003	0.0004	0.0003	0.0004	0.0004	0.0004	0.0003	0.0004	0.0004	0.0005	0.0016	0.0005	0.00043	0.0003	0.0003	0.00039	0.0003	0.0003	0.0003
132	10.465	323172	226	Amidosulfon	0.0001	0.00016	0.0001	0.0001	5E-05	6E-05	8.9E-05	6E-05	9E-05	8E-05	4E-05	3E-05	5E-05	5E-05	5E-05	0.00143	6E-05	7.7E-05	2E-05	3E-06	5.6E-05	8E-05	3E-05	2E-05
133	10.583	323179	231.1	3,6-anhydro-	0.0009	0.00104	0.0009	0.0009	0.0007	0.0004	0.00107	0.0003	0.001	0.0002	0.0001	0.0003	0.0033	0.0002	0.0002	0.00012	0.0004	0.00096	0.0007	0.0001	0.00013	0.0001	0.0004	0.0003
136	10.653	323183	158.143	L-isoleucine	0.0356	0.04344	0.04	0.0408	0.0553	0.0649	0.00022	0.0774	0.049	0.0506	0.0602	0.0386	0.0226	0.0629	0.0805	0.06652	0.0524	0.05856	0.0431	0.0455	0.01589	0.056	0.0135	0.0329
139	10.739	323188	142.1	L-proline	0.0685	0.08302	0.0783	0.0788	0.1196	0.142	0.08676	0.1725	0.1006	0.0534	0.0824	0.0644	0.0657	0.081	0.194	0.18482	0.0955	0.08857	0.1149	0.1194	0.03908	0.1007	0.033	0.067
141	10.867	323196	174.1	Glycine	0.0358	0.04371	0.045	0.0467	0.0554	0.0659	0.05944	0.0773	0.0668	0.0266	0.0353	0.0187	0.0231	0.0369	0.1055	0.05425	0.0418	0.03274	0.1034	0.0401	0.01839	0.044	0.029	0.0262
144	11.013	323204	240.1	1,3-dihydrox	0.0005	0.00063	0.0007	0.0007	0.0007	0.0006	0.00066	0.0007	0.0008	0.0007	0.0009	0.0006	0.0006	0.001	0.0006	0.00059	0.0007	0.00058	0.0006	0.0003	0.00045	0.0003	0.0004	0.0005
145	11.045	323206	147.1	Isocitric acid	0.004	0.00425	0.0042	0.0042	0.005	0.0033	0.003	0.0044	0.0046	0.0058	0.0077	0.0063	0.0045	0.0095	0.0042	0.00259	0.0047	0.00389	0.0032	0.0048	0.00479	0.0029	0.0041	0.0034
146	11.046	323206	247.083	Succinic acid	0.0004	0.00046	0.0004	0.0004	0.0006	0.0003	0.0003	0.0005	0.0005	0.0006	0.0009	0.0007	0.0005	0.001	0.0005	0.00025	0.0004	0.00039	0.0003	0.0006	0.00054	0.0003	0.0004	0.0003
147	11.106	323210	130.1	Chlorphenter	0.0003	0.00082	0.0003	0.0004	0.0026	0.0004	0.00028	0.0039	0.0026	0.001	0.0043	0.0024	0.0037	0.0042	0.0025	0.00036	0.0014	0.00042	0.0003	0.0017	0.0008	0.0017	0.0004	0.0003
149	11.257	323219	189.1	Glyceric acid	0.0038	0.0042	0.0042	0.0043	0.0064	0.0021	0.00314	0.0022	0.0034	0.0069	0.0074	0.0029	0.0024	0.0077	0.0051	0.00228	0.0032	0.00314	0.0028	0.0043	0.00515	0.0023	0.0045	0.0047
153	11.408	381024	147.1	5-hydroxype	0.0015	0.00105	0.0016	0.0016	0.0015	0.001	0.00122	0.001	0.0014	0.0029	0.0035	0.002	0.0016	0.0034	0.0012	0.00247	0.0019	0.00156	0.0009	0.0019	0.00228	0.0009	0.0026	0.0018
156	11.61	381030	292.136	4-deoxyeryth	0.0005	0.00066	0.0007	0.0006	0.0006	0.0002	0.00068	0.0002	0.0008	0.0007	0.0008	0.0007	0.0005	0.0008	0.0004	0.00201	0.0024	0.00034	0.0007	0.0003	0.00039	0.0003	0.0003	0.0004
157	11.651	381032	245.092	Fumaric acid	0.0011	0.00134	0.0014	0.0015	0.0022	0.0012	0.00107	0.0019	0.0019	0.0012	0.0027	0.0013	0.0014	0.0035	0.0026	0.00252	0.0016	0.00128	0.0011	0.0011	0.00143	0.001	0.0009	0.0011
159	11.756	381035	204.1	Serine	0.0285	0.03468	0.0283	0.0312	0.0448	0.0446	0.03733	0.0564	0.0522	0.0292	0.0341	0.0255	0.0214	0.0358	0.0875	0.03681	0.0351	0.03053	0.043	0.0402	0.02284	0.0395	0.0219	0.0246
160	11.781	381036	293.136	Dihydroxyma	0.0003	0.00039	0.0003	0.0004	0.0002	0.0002	0.00024	0.0003	0.0003	0.0007	0.0007	0.0004	0.0003	0.0009	0.0002	0.0002	0.0005	0.00021	0.0002	0.0003	0.00037	0.0002	0.0002	0.0002
161	11.837	381037	215.136	Pelargonic ac	0.0012	0.00122	0.0011	0.0011	0.0018	0.0013	0.00157	0.0013	0.0016	0.0012	0.0014	0.0013	0.0014	0.0015	0.0012	0.00112	0.001	0.00097	0.0014	0.0015	0.00112	0.0011	0.001	0.001
162	11.853	381038	156.116	Pipecolic aci	0.0006	0.00069	0.0005	0.0006	0.0011	0.0011	0.00054	0.0014	0.0008	0.0004	0.0006	0.0007	0.0005	0.0007	0.001	0.00102	0.001	0.00059	0.0007	0.0004	0.00039	0.0007	0.0006	0.0016
165	12.165	381048	218.116	L-threonine	0.0193	0.02158	0.02	0.0206	0.0269	0.0238	0.02401	0.0268	0.0292	0.0151	0.0173	0.0179	0.0128	0.0184	0.0404	0.03313	0.0199	0.02857	0.0235	0.0249	0.0106	0.0246	0.0146	0.0119
167	12.189	381048	263.091	L-threonic ac	0.0005	0.00059	0.0006	0.0006	0.0007	0.0004	0.00056	0.0005	0.0007	0.0006	0.0008	0.0005	0.0006	0.0009	0.0004	0.00079	0.0009	0.00047	0.0005	0.0003	0.00034	0.0003	0.0004	0.0003
170	12.538	381059	103.1	1,2,4-butanet	5E-05	0.00107	4E-05	0.0011	0.0008	0.0005	2.2E-05	0.0005	3E-05	0.0006	0.0007	0.0007	0.0012	0.0007	5E-05	0.00391	0.0021	5.1E-05	0.0013	4E-05	2.7E-05	5E-05	3E-05	4E-05
173	12.875	381070	248.13	Beta-alanine	0.0004	0.00038	0.0004	0.0004	0.0007	0.0003	0.00029	0.0004	0.0004	0.0004	0.0007	0.0002	0.0003	0.0008	0.0004	0.00064	0.0005	0.00076	0.0009	0.0002	0.00018	0.0003	0.0002	0.0004
174	12.916	381071	174.1	Putrescine	0.0535	0.05855	0.0589	0.0599	0.0666	0.0598	0.05677	0.0685	0.0637	0.0543	0.0677	0.0536	0.0642	0.0706	0.0605	0.0595	0.0557	0.05899	0.0595	0.048	0.04872	0.0466	0.0482	0.0515
175	12.933	381072	233.094	(s)-3,4-dihyd	0.0004	0.00045	0.0004	0.0005	0.0003	0.0002	0.00035	0.0003	0.0004	0.0004	0.0004	0.0003	0.0006	0.0004	0.0004	0.0011	0.0008	0.00035	0.0008	0.0004	0.00019	0.0004	0.0003	0.0005
177	13.064	381076	172.1	N-methyl-l-le	0.0004	0.00036	0.0004	0.0004	0.0017	0.0018	0.0001	0.0018	4E-05	0.0002	0.0002	0.0002	0.0001	0.0002	0.0001	0.0019	1E-04	8.5E-05	8E-05	0.0001	5.6E-05	0.0004	0.0001	6E-05
178	13.109	381077	109.1	Pentobarbital	0.0006	0.00065	0.0006	0.0006	0.0002	3E-05	0.00011	5E-05	0.0002	0.0025	0.0027	0.0001	0.0004	0.0029	0.0002	0.00025	7E-05	0.00066	7E-05	0.0005	0.00045	0.0011	0.0002	0.0001
180	13.247	381081	350.2	Phosphenodi	0.0065	0.00627	0.0064	0.007	0.0035	0.0048	0.0042	0.0038	0.004	0.0093	0.0073	0.0043	0.0033	0.0081	0.0034	0.00945	0.0076	0.00437	0.0048	0.0044	0.0059	0.0036	0.0034	0.0044
183	13.395	381086	229.173	Capric acid	0.0008	0.00087	0.0008	0.0008	0.0013	0.0009	0.00088	0.0009	0.0009	0.0011	0.0013	0.001	0.0011	0.0013	0.0005	0.00123	0.0014	0.00116	0.0012	0.0035	0.00104	0.0008	0.0009	0.0009
185	13.525	381090	174.1	Tyramine	0.0102	0.01083	0.0111	0.0112	0.013	0.0101	0.01003	0.0115	0.011	0.0173	0.0194	0.0109	0.0081	0.02	0.0137	0.00644	0.0089	0.00929	0.0148	0.015	0.0171	0.0079	0.0208	0.0149
186	13.526	381090	147.1	Aminomalon	0.0597	0.06388	0.0649	0.066	0.0771	0.0563	0.05451	0.0653	0.0616	0.1053	0.1172	0.0707	0.0494	0.1208	0.0773	0.03936	0.0522	0.05694	0.0812	0.0894	0.10274	0.0445	0.124	0.0912
187	13.607	381093	155.104	L-glutamine	0.0017	0.00228	0.0016	0.0018	0.0025	0.0025	0.00162	0.0027	0.0019	0.0014	0.0017	0.0002	0.0011	0.002	0.0056	0.00239	0.0018	0.00223	0.0028	0.0044	0.00286	0.0032	0.0015	0.0016
189	13.688	381095	160.082	N-carbamoyl	0.0016	0.00175	0.0017	0.0017	0.0021	0.0007	0.00142	0.0007	0.0015	0.0029	0.0033	0.0006	0.0012	0.0033	0.0006	0.00101	0.0005	0.00329	0.002	0.0007	0.00112	0.0021	0.0009	0.0012
190	13.786	381098	257.2	L-cysteine-gl	0.0001	0.00011	0.0001	0.0002	0.0002	0.0001	0.00012	0.0002	0.0002	0.0001	0.0002	0.0001	2E-05	0.0002	0.0001	0.00018	0.0002	0.00015	0.0002	1E-04	0.00012	0.0001	4E-05	0.0002
191	13.815	381099	233.1	Malic acid	0.0008	0.00086	0.0007	0.0008	0.0005	0.0006	0.00036	0.0005	0.0003	0.0006	0.0005	0.0009	0.0003	0.0007	0.003	0.00043	0.001	0.00082	0.0007	0.0007	0.00077	0.0004	0.0006	0.0006
192	13.868	381101	174.114	N-acetylornit	0.0004	0.00055	0.0005	0.0006	0.001	0.0005	0.00057	0.0011	0.0012	0.0003	0.0011	0.0004	0.001	0.0015	0.0005	0.00063	0.0004	0.00046	0.0005	0.0003	0.00032	0.0003	0.0003	0.0004
193	13.898	381102	85.1	Tetracosane	0.0017	0.00173	0.0017	0.0018	0.0019	0.0016	0.00174	0.0018	0.0018	0.0016	0.0018	0.0016	0.0018	0.002	0.0017	0.00177	0.0017	0.00167	0.0016	0.0016	0.00156	0.0016	0.0015	0.0016
194	13.932	381103	186.111	Alloxanoic ac	0.0002	0.00025	0.0003	0.0003	0.0005	0.0004	0.00024	0.0005	0.0003	0.0003	0.0005	0.0002	0.0003	0.0004	0.0002	0.00013	0.0002	0.00037	0.0002	0.0001	0.00028	0.0002	0.0002	0.0004
195	13.97	381104	217.133	Erythritol	0.0007	0.00079	0.0008	0.0008	0.0006	0.0003	0.00039	0.0003	0.0005	0.0006	0.0007	0.0004	0.0004	0.0008	0.0005	0.00649	0.0011	0.00058	0.0007	0.0005	0.00025	0.0003	0.0004	0.0005
199	14.05	381107	232.136	Serotonin	0.0006	0.00069	0.0008	0.0009	0.002	0.0009	0.00077	0.0015	0.0013	0.0006	0.0013	0.0007	0.0012	0.0013	0.0009	0.00074	0.0007	0.00083	0.001	0.0004	0.00048	0.0005	0.0005	0.0006
201	14.133	381109	143.1	Phytol	0.0002	0.00026	0.0007	0.0003	0.0004	0.0002	0.00061	0.0002	0.0004	0.0003	0.0002	0.0004	0.0003	0.0002	0.0008	0.00313	0.0007	0.00042	0.0005	0.0002	0.0003	0.0004	0.0004	0.0008
203	14.168	381110	267.1	Salicylic acid	0.0085	0.009	0.0088	0.009	0.0169	0.0002	0.00025	0.0001	0.0002	0.0532	0.0592	0.0261	6E-05	0.0619	7E-05	0.01029	0.0308	0.01327	0.0187	9E-05	8.4E-05	9E-05	0.0112	0.0001
205	14.249	381113	317	N-acetyl-d-m	1E-05	1.3E-05	1E-05	2E-05	3E-06	1E-06	1.1E-06	3E-06	2E-05	1E-06	4E-06	1E-06	2E-06	3E-06	1E-06	0.00017	3E-06	1.1E-06	4E-05	1E-06	1.1E-06	1E-06	1E-06	1E-06
206	14.307	381115	176.1	L-methionine	0.0065	0.00714	0.0069	0.0071	0.0082	0.0099	0.0071	0.0109	0.008	0.0063	0.0075	0.0053	0.0041	0.0077	0.0133	0.01075	0.0083	0.00938	0.0068	0.0072	0.00295	0.0088	0.0027	0.0047
207	14.328	381115	232.1	L-aspartic ac	0.0043	0.00456	0.0043	0.0046	0.0055	0.0067	0.00459	0.0076	0.0053	0.0035	0.0043	0.0034	0.0036	0.0047	0.0073	0.00969	0.0041	0.00558	0.0046	0.0032	0.00294	0.0054	0.0016	0.0043
208	14.349	381116	156.1	Oxoproline	0.1241	0.13078	0.128	0.1314	0.1436	0.1309	0.11217	0.1359	0.1217	0.1354	0.156	0.1537	0.1068	0.1636	0.1604	0.17866	0.1098	0.13079	0.1212	0.1374	0.11108	0.1222	0.105	0.1057
209	14.416	381118	230.15	4-hydroxypro	0.0074	0.00808	0.0078	0.0079	0.0231	0.0094	0.00908	0.0101	0.0099	0.0027	0.003	0.0045	0.0038	0.0033	0.0071	0.05472	0.0025	0.00687	0.0154	0.0033	0.00244	0.0106	0.0017	0.0056
213	14.484	487220	214.2	Diclofenac	0.0653	0.06762	0.0676	0.0678	0.0691	0.0666	0.06853	0.0715	0.0732	0.0643	0.069	0.0651	0.0727	0.0712	0.0657	0.06502	0.0655	0.06643	0.067	0.0629	0.06411	0.0602	0.0622	0.0651
217	14.639	487225	263.18	2,6-bis(tert-b	0.0004	0.00043	0.0004	0.0004	0.0004	0.0004	0.00037	0.0005	0.0004	0.0004	0.0005	0.0004	0.0004	0.0005	0.0004	0.00037	0.0003	0.0004	0.0004	0.0004	0.00031	0.0003	0.0003	0.0004
220	14.722	487228	260.188	Aminoadipic	0.0102	0.01158	0.0119	0.0119	0.0191	0.0091	0.00816	0.011	0.0098	0.024	0.0295	0.0215	0.0135	0.0296	0.0054	0.0096	0.0102	0.01726	0.0099	0.0111	0.01709	0.0053	0.0139	0.0193
222	14.816	487232	115.1	Creatinine	0.0098	0.01016	0.0103	0.01	0.0162	0.0098	0.0077	0.01	0.0079	0.0095	0.0094	0.0075	0.0101	0.0089	0.0079	0.09164	0.011	0.01077	0.01	0.0062	0.00366	0.0068	0.0067	0.011
223	14.847	487233	220.1	L-cysteine	0.0036	0.00401	0.0039	0.004	0.005	0.0042	0.00354	0.0047	0.0042	0.0039	0.005	0.0029	0.0024	0.0052	0.0047	0.00887	0.0036	0.0041	0.005	0.0028	0.0034	0.0025	0.0038	0.0038
225	14.875	487234	292.165	Erythronic ac	0.0028	0.00292	0.0029	0.003	0.0038	0.0015	0.00271	0.0015	0.0029	0.0039	0.0043	0.0017	0.001	0.0047	0.003	0.0053	0.0019	0.00231	0.0016	0.0021	0.00439	0.001	0.0049	0.0042
226	14.947	487236	185.091	2-piperidinob	0.0004	0.00038	0.0005	0.0004	0.0004	0.0004	0.00047	0.0005	0.0007	0.0005	0.0006	0.0004	0.0003	0.0006	0.0005	0.00059	0.0006	0.00056	0.0002	0.0004	0.00045	0.0005	0.0003	0.0004
227	15.019	487239	345.138	Quinic acid	1E-04	0.0001	1E-04	9E-05	7E-05	4E-05	7.7E-05	6E-05	0.0001	2E-05	2E-05	4E-05	0.0002	5E-05	4E-05	3.1E-05	5E-05	9.4E-05	3E-05	5E-06	1.4E-05	2E-05	5E-05	2E-05
229	15.106	487241	192.071	L-kynurenine	0.0014	0.00145	0.0014	0.0015	0.0006	0.0009	0.00085	0.0012	0.0013	0.0028	0.0039	0.0013	0.001	0.0049	0.0005	0.00085	0.0026	0.00071	0.0005	0.0006	0.00131	0.0005	0.0004	0.0006
230	15.142	487243	247.12	L-2-hydroxy	0.0003	0.00026	0.0003	0.0003	0.0002	0.0002	0.00019	0.0002	0.0002	0.0002	0.0002	0.0002	0.0002	0.0002	0.0003	0.00028	0.0003	0.00024	0.0002	0.0002	0.00019	0.0003	0.0002	0.0002
231	15.168	487244	198.069	Oxoglutaric a	0.0007	0.00067	0.0007	0.0007	0.0005	0.0005	0.00048	0.0006	0.0005	0.001	0.0011	0.0033	0.0006	0.0012	0.0007	0.00022	0.0006	0.00029	0.0002	0.0004	0.00041	0.0004	0.002	0.0004
232	15.181	487244	216.1	5-methoxyps	0.0002	0.00013	0.0002	0.0001	0.0002	0.0001	6.4E-05	0.0001	6E-05	0.0002	0.0001	0.0002	8E-05	0.0001	0.0001	7.7E-05	0.0001	0.00018	0.0001	0.0001	0.00015	0.0001	0.0002	0.0002
233	15.223	487246	171.1	Glycocyamin	0.0002	0.00033	0.0003	0.0003	0.0004	0.0005	0.00033	0.0002	0.0004	0.0003	0.0004	0.0002	0.0003	0.0004	0.0005	0.00305	0.0003	0.00066	0.0005	0.0007	0.00073	0.0003	0.0005	0.0007
236	15.443	487253	357.1	Phosphoglyc	5E-05	7.7E-05	0.0001	8E-05	0.0004	0.0001	9.8E-05	0.0005	0.0004	4E-05	0.0004	5E-05	0.0005	0.0004	7E-05	8.6E-05	9E-05	8.2E-05	0.0001	1E-05	2.2E-05	2E-05	3E-05	5E-05
237	15.51	487255	232.127	Nicotianamir	0.0006	0.00065	0.0007	0.0007	0.0007	0.0006	0.00074	0.0007	0.0008	0.0009	0.0011	0.0006	0.0007	0.0011	0.0005	0.00043	0.0007	0.00075	0.0005	0.0006	0.0011	0.0005	0.0006	0.0007
240	15.531	487256	147.1	N-acetylaspa	0.006	0.00631	0.0064	0.0066	0.0088	0.0051	0.00411	0.0058	0.0025	0.0138	0.0158	0.0035	0.0054	0.0156	0.0033	0.00697	0.0067	0.00945	0.005	0.0066	0.00772	0.0044	0.007	0.0093
241	15.607	487259	156.111	Glutamyl-val	0.0012	0.00131	0.0012	0.0012	0.0014	0.0007	0.00064	0.0007	0.0007	0.0017	0.0017	0.0016	0.0008	0.0017	0.0014	0.00432	0.0006	0.00168	0.0006	0.0015	0.00411	0.001	0.0035	0.0028
243	15.636	487260	217.122	Glucose-1-ph	0.0008	0.00106	0.0008	0.001	0.0009	0.0014	0.00116	0.0013	0.0012	0.0009	0.0011	0.0008	0.0009	0.0015	0.0015	0.00104	0.0015	0.00075	0.0009	0.0015	0.00082	0.0009	0.0007	0.0007
246	15.769	487264	244.032	3-methylene-	0.0033	0.0037	0.0041	0.0042	0.0027	0.003	0.00309	0.0032	0.0036	0.0041	0.0046	0.0028	0.0025	0.0048	0.0027	0.00293	0.0042	0.00283	0.0024	0.0019	0.00276	0.0021	0.0017	0.0025
247	15.802	487265	246.108	L-glutamic ac	0.0312	0.0335	0.0334	0.0338	0.0438	0.0425	0.03548	0.047	0.0395	0.0362	0.0422	0.0583	0.0231	0.0443	0.0423	0.04731	0.0285	0.02989	0.0263	0.0307	0.01761	0.0409	0.0206	0.0303

250	15.903	487269	218.1	L-phenylalan	0.019	0.02024	0.0203	0.0204	0.0228	0.026	0.01722	0.0287	0.0195	0.0208	0.0246	0.0151	0.014	0.0256	0.0283	0.03121	0.031	0.02359	0.0203	0.021	0.01093	0.0246	0.0094	0.0136
252	15.941	487270	248.125	Glycerol-3-g	0.0004	0.00039	0.0003	0.0003	0.0004	0.0002	0.00018	0.0002	0.0002	0.0007	0.001	0.0005	0.0003	0.0012	0.0002	0.0003	0.0003	0.00032	0.0003	0.0005	0.00057	0.0001	0.0007	0.0004
253	15.959	487271	267.1	3-hydroxybe	0.0002	0.00016	0.0002	0.0002	9E-05	0.0001	7.6E-05	7E-05	6E-05	6E-05	8E-05	7E-05	4E-05	0.0001	0.0008	0.00154	0.0002	0.00017	0.0001	0.0006	9.7E-05	0.0006	6E-05	9E-05
254	16.021	487273	204.1	2,4-diaminob	0.0015	0.00191	0.0018	0.0019	0.0013	0.0008	0.00146	0.0013	0.0021	0.0037	0.0063	0.0016	0.0024	0.0063	0.0011	0.00155	0.005	0.00116	0.0008	0.0007	0.00084	0.0006	0.0006	0.0005
255	16.102	487276	335.15	D-xylulose	0.0007	0.00071	0.0006	0.0007	0.0008	0.0003	0.00042	0.0003	0.0004	0.0011	0.0015	0.001	0.0006	0.0018	0.0003	0.0008	0.0005	0.0009	0.0005	0.0009	0.00081	0.0002	0.0016	0.0005
256	16.12	487276	179.088	4-hydroxyph	0.0003	0.00031	0.0003	0.0004	0.0002	0.0002	0.00018	0.0002	0.0002	0.0001	0.0002	0.0002	0.0001	0.0003	0.0001	0.00171	0.0018	0.00043	0.0002	0.0001	9.8E-05	0.0002	9E-05	0.0001
260	16.259	487281	307.125	D-ribose	0.0002	0.00018	0.0002	0.0002	0.0002	0.0001	0.0001	0.0001	0.0001	0.0002	0.0002	0.0001	5E-05	0.0002	0.0002	0.00259	0.0003	0.00011	0.0002	0.0001	5.4E-05	0.0001	7E-05	7E-05
261	16.277	487282	201.1	3-hydroxycar	0.0002	0.00018	0.0001	7E-05	4E-05	9E-05	8E-05	5E-05	0.0001	8E-05	8E-05	3E-05	3E-05	8E-05	0.0002	0.0005	0.0002	5.3E-05	0.0002	0.0002	0.00015	2E-05	0.0001	7E-05
262	16.309	487283	147.1	2-deoxypento	0.0012	0.00139	0.0013	0.0013	0.0013	0.0006	0.00118	0.0009	0.0012	0.0005	0.0002	0.0009	0.0009	0.0008	0.0005	0.01719	0.0022	0.00113	0.0008	0.0007	0.00061	0.0007	0.0002	0.0006
263	16.324	487283	257.196	Dodecanoic a	0.0016	0.00159	0.0011	0.0012	0.0006	0.0012	0.00105	0.0009	0.0009	0.0009	0.0007	0.0009	0.0006	0.0008	0.0009	0.00082	0.0015	0.00094	0.0008	0.0039	0.00207	0.0011	0.0014	0.001
264	16.346	487284	451.11	Pyrophospha	0.0043	0.00568	0.008	0.0052	0.0158	0.007	0.00767	0.0144	0.0162	0.0031	0.0097	0.0041	0.0133	0.0096	0.004	0.00714	0.0061	0.00868	0.0104	0.0005	0.00079	0.0007	0.0022	0.004
268	16.475	487289	326.113	Taurine	0.0008	0.00079	0.0007	0.0008	0.0009	0.001	0.00117	0.0011	0.0011	0.0004	0.0004	0.0006	0.0005	0.0004	0.0011	0.00095	0.0005	0.00111	0.0016	0.0008	0.0006	0.0008	0.0013	0.0007
270	16.512	487290	116.1	L-asparagine	0.0019	0.00237	0.0022	0.0023	0.0031	0.003	0.00257	0.0033	0.0028	0.0014	0.0019	0.0009	0.001	0.002	0.0062	0.00393	0.0024	0.00243	0.0038	0.0021	0.00106	0.0033	0.0012	0.0019
271	16.572	487292	103	D-arabinose	0.001	0.001	0.0011	0.0011	0.0003	0.0007	0.00077	0.0008	0.0009	0.0017	0.0003	0.0011	0.0001	0.0003	0.0008	0.00279	0.001	0.00092	0.001	0.0018	0.00133	0.0007	0.0016	0.0002
273	16.769	487299	218.1	Glucosamini	1E-04	7.5E-05	8E-05	9E-05	6E-05	5E-05	5.6E-05	8E-05	5E-05	3E-05	5E-05	3E-05	3E-05	8E-05	0.0002	0.0015	0.0001	3.2E-05	1E-04	0.0001	8.8E-05	4E-05	2E-05	3E-05
275	16.872	487302	282.111	4-(dimethyla	0.0006	0.00064	0.0006	0.0007	0.0003	0.0004	0.00046	0.0005	0.0006	0.0012	0.0017	0.0006	0.0005	0.0021	0.0003	0.00046	0.0012	0.00037	0.0003	0.0004	0.00067	0.0003	0.0003	0.0003
279	16.969	487306	319.14	Ribitol	0.0002	0.00027	0.0003	0.0003	0.0005	0.0004	0.00028	0.0004	0.0003	0.0002	0.0003	0.0001	0.0002	0.0003	0.0006	0.00043	0.0003	0.00039	0.0005	0.0003	0.00023	0.0003	0.0002	0.0003
282	17.037	487308	218.089	Dl-dopa	0.0019	0.00207	0.0019	0.002	0.0028	0.0039	0.00116	0.0038	0.0012	0.0011	0.001	0.002	0.0026	0.0011	0.0018	0.00439	0.0029	0.00461	0.0069	0.0011	0.00108	0.0011	0.001	0.0028
285	17.07	487309	277.1	Indoxyl sulfa	0.002	0.00224	0.0024	0.0025	0.0008	0.0008	0.00124	0.0012	0.0019	0.0008	0.0012	0.0006	0.0015	0.0014	0.0006	0.04522	1E-04	0.0007	0.0002	0.0002	0.00039	0.0014	0.0003	6E-05
288	17.16	487312	103.1	2-monoolein	0.0025	0.00264	0.0026	0.0026	0.0016	0.0013	0.00147	0.0015	0.0017	0.0022	0.0024	0.0017	0.0022	0.0024	0.002	0.01386	0.0043	0.00198	0.0022	0.0014	0.0011	0.0016	0.0034	0.0014
289	17.161	487312	217.106	L-arabitol	0.0035	0.00365	0.0036	0.0035	0.0023	0.0019	0.00207	0.0022	0.0023	0.003	0.0034	0.0025	0.003	0.0037	0.0028	0.01819	0.006	0.00264	0.0031	0.0019	0.00155	0.0019	0.0044	0.002
290	17.234	487315	305.164	Hexitol	0.0008	0.00096	0.0006	0.0008	0.0008	0.0015	0.00119	0.0017	0.0016	0.0008	0.001	0.0006	0.0008	0.0014	0.0015	0.00074	0.0013	0.00073	0.0005	0.0012	0.00063	0.0008	0.0003	0.0004
291	17.255	487315	299.06	Glucose-6-ph	0.0001	0.00016	0.0002	0.0001	0.0001	0.0002	0.00019	0.0002	0.0002	0.0001	0.0001	0.0001	0.0001	0.0002	0.0001	0.00022	0.0002	0.00019	0.0001	0.0002	0.00016	0.0002	0.0002	0.0002
295	17.39	582620	199.2	Ergosterol	1.9467	1.93669	1.9466	1.9436	1.9865	1.9106	1.94093	1.9735	1.9613	1.8845	1.9446	1.9061	2.0051	1.9653	1.9364	1.96779	1.8656	1.92597	1.9438	1.8969	1.94837	1.7927	1.9206	1.9319
297	17.498	582624	217.1	Udp-glucuro	0.0039	0.00363	0.0033	0.003	0.0014	0.0016	0.00123	0.0014	0.001	0.002	0.0022	0.0005	0.0009	0.0021	0.0009	0.0456	0.0044	0.00168	0.0024	0.0016	0.00161	0.0039	0.0012	0.0015
299	17.524	582625	232.095	Beta-glutami	0.0084	0.00919	0.0088	0.0086	0.0094	0.0063	0.00576	0.0078	0.0072	0.0185	0.0189	0.0153	0.0062	0.0197	0.0062	0.00618	0.007	0.00766	0.0056	0.0064	0.01606	0.0017	0.005	0.0063
301	17.616	582629	229.085	Aconitic acid	0.0022	0.00191	0.0022	0.0019	0.0018	0.0017	0.00184	0.0016	0.001	0.0019	0.0013	0.0017	0.0014	0.0011	0.004	0.0085	0.0023	0.00233	0.0025	0.0017	0.0018	0.0015	0.0023	0.0019
302	17.68	582631	274.123	4-aminobutyr	0.0015	0.00184	0.0018	0.0018	0.0034	0.0024	0.00192	0.0032	0.0029	0.0015	0.0026	0.0015	0.0029	0.0027	0.0019	0.00208	0.0018	0.00194	0.0018	0.0013	0.00142	0.0011	0.0016	0.0018
303	17.696	582632	297.1	3-hydroxy-3-	0.0021	0.00267	0.0026	0.0029	0.0019	0.0012	0.01926	0.0014	0.026	0.0013	0.0021	0.0034	0.0014	0.002	0.0003	0.00106	0.004	0.00034	0.0003	0.0003	0.00384	1E-06	0.0026	0.0013
305	17.751	582634	357.127	Glycerol 3-pl	0.0082	0.00954	0.007	0.0075	0.0079	0.0069	0.00501	0.0057	0.005	0.0102	0.0113	0.0084	0.0059	0.0138	0.0125	0.00836	0.0045	0.00672	0.0043	0.0149	0.012	0.0086	0.0136	0.0084
306	17.815	582637	217.1	Xylonolactor	0.0082	0.00935	0.0097	0.0087	0.0121	0.014	0.01466	0.0226	0.0224	0.0078	0.0128	0.0081	0.0094	0.0163	0.0132	0.01224	0.0164	0.0086	0.0071	0.0049	0.0046	0.0049	0.0036	0.0039
309	17.871	582639	257.091	Udp-n-acetyl	0.0031	0.00251	0.0028	0.0026	0.0044	0.0015	0.0017	0.0021	0.0021	0.0051	0.0077	0.0017	0.0028	0.0075	0.0035	0.00529	0.0022	0.00378	0.0026	0.0036	0.00422	0.0023	0.0045	0.004
310	17.882	582639	292.109	Galactonic ac	0.002	0.00203	0.0021	0.0021	0.0014	0.0009	0.00113	0.0009	0.0012	0.0021	0.0023	0.0012	0.0009	0.0026	0.0024	0.01338	0.0026	0.00255	0.0016	0.0016	0.00096	0.0007	0.0015	0.0011
311	17.933	582641	217.1	Uridine	0.0033	0.00351	0.0362	0.0034	0.0034	0.0467	0.00207	0.0634	0.0625	0.0032	0.0035	0.006	0.0027	0.0038	0.0459	0.02221	0.0113	0.00271	0.006	0.0357	0.006	0.0022	0.0121	0.0021
313	17.949	582642	156.1	L-glutamine	0.2209	0.24557	0.2532	0.2532	0.3565	0.3004	0.2342	0.346	0.2579	0.1318	0.1688	0.0121	0.1125	0.1718	0.6392	0.33488	0.277	0.3988	0.5541	0.2948	0.23144	0.2791	0.1777	0.2251
314	17.971	582643	292.163	2-keto-l-gluc	0.0058	0.00626	0.0059	0.0063	0.0038	0.0022	0.00409	0.0026	0.0045	0.0035	0.0039	0.0032	0.0029	0.004	0.0043	0.04645	0.0056	0.00488	0.0044	0.0037	0.00406	0.0035	0.0034	0.003
316	18.001	582644	217.1	Xylofuranose	0.0219	0.02434	0.0247	0.026	0.0348	0.0369	0.03963	0.0538	0.0598	0.0241	0.0371	0.0227	0.027	0.0456	0.0323	0.0578	0.0402	0.02336	0.0191	0.013	0.01122	0.0122	0.0091	0.0109
318	18.038	582645	299.08	Phosphoetha	0.0015	0.00208	0.0015	0.0017	0.0045	0.0018	0.00225	0.003	0.0028	0.002	0.0026	0.0016	0.0029	0.0032	0.0015	0.00298	0.0019	0.00222	0.0021	0.0013	0.00155	0.001	0.0018	0.0011
319	18.059	582646	204.1	Glucose	0.0771	0.06365	0.0441	0.0344	0.0104	0.0186	0.04281	0.0053	0.0106	0.0202	0.0047	0.03	0.0533	0.0022	0.0124	0.00027	0.0224	0.06868	0.0181	0.0101	0.01817	0.0138	0.093	0.0394
320	18.089	582647	271.105	Trehalose-6-	0.0069	0.00747	0.0063	0.0069	0.0084	0.0038	0.00402	0.0037	0.0038	0.0103	0.0105	0.0012	0.006	0.01	0.0052	0.00572	0.0051	0.00993	0.0076	0.0105	0.02224	0.004	0.0174	0.011
322	18.203	582652	295.1	Terephthalic	0.0126	0.00858	0.0077	0.0069	0.005	0.0092	0.00741	0.0044	0.0047	0.0112	0.0045	0.0091	0.0048	0.0038	0.0084	0.00854	0.0071	0.00767	0.0075	0.023	0.02032	0.0127	0.0148	0.0137
323	18.227	582653	288.118	Ascorbic acid	0.0011	0.00117	0.0014	0.0013	0.0028	0.001	0.00061	0.0012	0.0012	0.0013	0.0027	0.0007	0.0014	0.003	0.0009	0.00044	0.0007	0.0016	0.0009	0.0009	0.00213	0.0005	0.0009	0.0017
325	18.288	582655	217.1	D-fructose	0.5263	0.53684	0.5383	0.5367	0.3509	0.283	0.6246	0.3015	0.6521	0.2243	0.2471	0.2702	1.0885	0.255	0.1956	0.09033	0.4294	0.52613	0.1864	0.0944	0.22529	0.1636	0.3421	0.2587
326	18.314	582656	204.1	Pectin	0.1957	0.19968	0.2012	0.1979	0.1566	0.2089	0.43442	0.2193	0.4533	0.1766	0.1905	0.2835	0.4026	0.1963	0.073	0.1983	0.6907	0.32689	0.2005	0.0574	0.11458	0.0389	0.1147	0.1051
327	18.342	582657	357.05	3-phosphogly	0.0006	6E-05	0.0006	0.0006	0.001	0.0012	0.00092	0.0012	0.0001	0.0006	0.0007	0.0005	0.0005	0.0008	0.0008	0.00057	0.0009	0.00142	0.0009	0.0004	0.00051	0.0003	0.0009	0.0008
329	18.418	582660	265.1	Hypoxanthin	0.0157	0.01647	0.0162	0.0176	0.01	0.0139	0.00791	0.0144	0.0085	0.0119	0.0119	0.0131	0.0149	0.0115	0.0132	0.00323	0.0073	0.02593	0.0074	0.0078	0.01518	0.0056	0.0549	0.014
331	18.448	582661	204.1	L-sorbose	0.2263	0.24921	0.2622	0.27	0.2419	0.1129	0.26089	0.1306	0.3024	0.0792	0.0991	0.1048	0.8462	0.104	0.0812	0.02293	0.1159	0.27572	0.0896	0.0342	0.05952	0.0563	0.1626	0.1065
332	18.5	582663	142.1	Ornithine	0.1206	0.1253	0.1277	0.1263	0.183	0.1896	0.16492	0.2027	0.1727	0.1122	0.1282	0.1153	0.0479	0.129	0.3501	0.24588	0.1704	0.18014	0.1357	0.1621	0.11022	0.2539	0.0995	0.1279
333	18.515	582664	273.1	Citric acid	0.2842	0.2889	0.2833	0.2841	0.3653	0.294	0.29316	0.3019	0.3002	0.2935	0.3175	0.2271	0.1449	0.3269	0.5017	0.38487	0.288	0.35832	0.3545	0.3105	0.3051	0.1654	0.2688	0.2948
335	18.582	582666	156.078	O-phosphose	0.0041	0.00474	0.0045	0.0047	0.0041	0.0046	0.00569	0.0052	0.0066	0.012	0.0149	0.0078	0.0042	0.0169	0.0051	0.00728	0.0054	0.00368	0.0041	0.0062	0.00394	0.0035	0.0074	0.0065
336	18.625	582668	256.157	Citrulline	0.004	0.00471	0.0042	0.0043	0.0079	0.0049	0.00768	0.0055	0.0091	0.0041	0.0051	0.0029	0.0033	0.0055	0.0046	0.01386	0.0058	0.00699	0.0033	0.0055	0.00248	0.0085	0.002	0.0038
341	18.815	582675	157.1	N-acetyl-d-h	0.0377	0.03917	0.0315	0.0322	0.0328	0.0627	0.05268	0.0398	0.0423	0.0428	0.0337	0.0524	0.0382	0.0356	0.0534	0.02926	0.0625	0.0393	0.0248	0.0417	0.05319	0.0317	0.0336	0.0387
342	18.827	582676	304.2	2-monopalmi	0.0022	0.00268	0.0027	0.0013	0.0025	0.0028	0.0031	0.0034	0.004	0.0029	0.0031	0.003	0.0014	0.0038	0.002	0.00191	0.0024	0.0025	0.0018	0.001	0.00283	0.0013	0.002	0.0019
344	18.847	582677	204.1	D-fucose	0.056	0.04464	0.0421	0.0385	0.0981	0.08	0.10253	0.0783	0.1026	0.0638	0.069	0.0876	0.0546	0.0706	0.0796	0.02633	0.0822	0.08697	0.0447	0.0497	0.19348	0.06	0.103	0.129
345	18.914	582679	157.1	Sophorose	0.0219	0.02293	0.017	0.0462	0.0634	0.0436	0.0272	0.0254	0.0191	0.0226	0.015	0.0251	0.0086	0.0161	0.0529	0.01428	0.0301	0.01549	0.0106	0.0388	0.12082	0.0908	0.092	0.0751
347	18.943	582680	147.1	1,5-anhydrog	0.3451	0.34143	0.3432	0.3426	0.5558	0.0628	0.04512	0.0764	0.0474	0.2448	0.2341	0.0409	0.0557	0.2389	0.1438	0.09781	0.2204	0.13048	0.1164	0.6074	1.04565	0.7027	0.8147	0.6534
349	18.982	582682	105.1	Hippuric acid	0.01	0.01006	0.0093	0.0087	0.0132	0.0063	0.00468	0.0042	0.0031	0.009	0.006	0.0054	0.0019	0.0059	0.0075	0.16526	0.0082	0.00367	0.0042	0.0176	0.02424	0.018	0.0192	0.0154
350	18.991	582682	285.222	Myristic acid	0.0172	0.01802	0.015	0.0158	0.0089	0.0152	0.01178	0.0143	0.011	0.0108	0.0106	0.0245	0.0095	0.012	0.0102	0.00802	0.0159	0.0159	0.0156	0.0372	0.01393	0.0084	0.036	0.0086
351	19	582683	356.2	Citrazinic	0.0018	0.00204	0.0023	0.0023	0.0002	0.0064	2.3E-05	0.0078	0.0002	0.0006	0.0008	6E-05	2E-05	0.001	0.0179	0.00014	4E-05	7.8E-05	0.0236	4E-05	7.3E-05	1E-06	3E-05	7E-05
352	19.002	582683	174.133	5-methoxytry	0.0078	0.00872	0.006	0.0069	0.0058	0.0136	0.01132	0.0099	0.0084	0.0051	0.0042	0.008	0.003	0.0046	0.0195	0.01002	0.0077	0.00927	0.0097	0.0129	0.00429	0.0156	0.0044	0.0093
353	19.05	582685	245.1	2,4-dihydrox	0.0064	0.00659	0.0063	0.0068	0.0044	0.002	0.00556	0.0021	0.0055	0.0026	0.002	0.0036	0.0145	0.0024	0.0025	0.0467	0.004	0.0051	0.0026	0.0041	0.00226	0.0024	0.003	0.0055
355	19.163	582689	103.1	D-tagatose	0.2394	0.24688	0.2424	0.2437	0.1465	0.2726	0.26973	0.2774	0.2723	0.1467	0.1541	0.1186	0.1235	0.1572	0.6847	0.06161	0.1275	0.17661	0.0847	0.2264	0.11986	0.4074	0.0683	0.1292
357	19.318	582695	217.1	Palatinitol	0.2197	0.2237	0.224	0.2128	0.1951	0.2924	0.3327	0.2943	0.3315	0.2416	0.2614	0.2843	0.2449	0.2634	0.3498	0.22319	0.3983	0.25553	0.1705	0.1652	0.16001	0.1817	0.1217	0.1592
360	19.396	582698	307.1	Isochlorogen	0.0303	0.03206	0.0125	0.0309	0.0213	0.1111	0.11166	0.1161	0.1119	0.0694	0.0743	0.0657	0.0745	0.0764	0.0018	0.04267	0.087	0.07957	0.0456	0.0024	0.0103	0.0075	0.0185	0.0151
361	19.396	582698	290.127	5-hydroxy-3-	0.0063	0.00653	0.0053	0.006	0.0071	0.0029	0.00311	0.0023	0.0025	0.0152	0.0135	0.0179	0.0047	0.015	0.0015	0.00543	0.0043	0.00601	0.0035	0.0065	0.00898	0.0027	0.012	0.0105
362	19.436	582699	102.1	2-monostearl	0.0196	0.01474	0.0057	0.0021	0.0223	0.0162	0.00661	0.0055	0.0056	0.012	0.0081	0.0023	0.0062	0.0102	0.0885	0.02441	0.0052	0.01223	0.0297	0.0256	0.04142	0.0765	0.0127	0.02
365	19.469	582701	204.1	Phytosphingo	14.113	13.8821	14.035	13.655	13.511	16.149	16.8319	15.322	16.145	15.736	14.746	16.895	16.714	14.274	9.1635	15.1276	19.456	16.7539	13.038	10.099	13.5626	7.2569	12.741	13.981
369	19.563	582704	132.1	Coniferin	0.0059	0.00681	0.0061	0.0071	0.0105	0.0109	0.01585	0.0073	0.0112	0.0062	0.0059	0.009	0.0071	0.006	0.011	0.00717	0.011	0.01123	0.0056	0.0062	0.02966	0.0107	0.012	0.0184
370	19.584	582705	179.1	Syringic acid	0.0086	0.00878	0.0081	0.0079	0.0065	0.0082	0.00782	0.0069	0.0077	0.008	0.0073	0.0096	0.006	0.0071	0.0096	0.02648	0.013	0.00908	0.0057	0.0103	0.00814	0.0084	0.0065	0.0057
371	19.598	582706	204.1	D-mannose	0.1364	0.14588	0.1442	0.1407	0.1111	0.1276	0.29236	0.1634	0.2984	0.1361	0.1381	0.1965	0.28	0.1439	0.0655	0.14824	0.4775	0.23007	0.1245	0.04	0.12516	0.0215	0.086	0.0912
372	19.65	582708	204.1	Galactinol	0.0517	0.05556	0.0515	0.0516	0.0942	0.075	0.12999	0.1634	0.1243	0.0616	0.4697	0.0875	0.0635	0.0587	0.1027	0.036	0.5783	0.08625	0.0342	0.0459	0.28479	0.0849	0.1183	0.163
374	19.83	582715	154.1	L-histidine	0.0136	0.01703	0.0253	0.0237	0.0506	0.0318	0.02742	0.0605	0.0407	0.0133	0.0298	0.0158	0.0109	0.0238	0.057	0.06633	0.0228	0.06012	0.0405	0.0142	0.00581	0.0307	0.0089	0.0198
375	19.838	582715	174.1	L-lysine	0.1274	0.14126	0.145	0.1452	0.2101	0.1925	0.17374	0.2091	0.1833	0.117	0.1319	0.1294	0.0589	0.1319	0.3354	0.26248	0.121	0.20859	0.2083	0.1481	0.07845	0.2224	0.1033	0.1714
376	19.859	582716	319.2	Sorbitol	0.1539	0.16636	0.1662	0.1662	0.0588	0.0413	0.05126	0.0442	0.0515	0.1262	0.1297	0.0535	0.0601	0.1318	0.0749	3.0633	0.0705	0.03524	0.0695	0.0689	0.18275	0.0339	0.069	0.0245
377	19.933	582719	333.2	Galacturonic	0.0108	0.01188	0.0116	0.012	0.0047	0.0047	0.00612	0.0047	0.0067	0.0063	0.0064	0.0061	0.0058	0.0067	0.011	0.1126	0.0135	0.00568	0.0079	0.0067	0.00464	0.0078	0.0029	0.0038
380	20.032	668724	218.1	L-tyrosine	0.1438	0.16127	0.1603	0.1627	0.1927	0.2813	0.16476	0.2987	0.173	0.154	0.1726	0.1404	0.0832	0.1736	0.2639	0.15583	0.2491	0.21141	0.134	0.2106	0.10333	0.2202	0.0783	0.1122
382	20.097	668727	179.096	N-acetyl-l-ty	0.0094	0.01146	0.0107	0.0108	0.0137	0.007	0.00526	0.0088	0.0062	0.0264	0.0301	0.0192	0.0104	0.0312	0.0052	0.01485	0.011	0.01326	0.0056	0.0198	0.02389	0.0064	0.0186	0.0134
386	20.171	668731	299.224	Isopentadeca	0.0016	0.00195	0.0017	0.0015	0.0013	0.002	0.00148	0.0018	0.001	0.0012	0.0009	0.0021	0.0018	0.0011	0.0013	0.00083	0.0016	0.00162	0.0017	0.0027	0.00157	0.0008	0.0025	0.0011
388	20.204	668733	156.09	1-kestose	0.0026	0.00258	0.0026	0.0026	0.0029	0.004	0.00433	0.0039	0.004	0.0044	0.005	0.003	0.0023	0.0049	0.0036	0.00556	0.0046	0.00243	0.0025	0.0023	0.00327	0.0021	0.0023	0.0029
389	20.246	668735	373.19	13beta-ethyl-	0.0018	0.00203	0.0018	0.0018	0.0016	0.0011	0.00117	0.001	0.0011	0.0021	0.002	0.0013	0.002	0.0018	0.0009	0.01057	0.0018	0.00192	0.0013	0.0009	0.00093	0.0007	0.0013	0.0022
390	20.297	668737	361.192	Maltotriose	0.0025	0.00262	0.0028	0.0026	0.0032	0.0071	0.00567	0.0074	0.0058	0.0042	0.0044	0.004	0.0026	0.0045	0.0067	0.00617	0.009	0.00438	0.0034	0.003	0.00194	0.0026	0.0015	0.0019
397	20.442	668745	385.163	2-amino[1,3]	0.001	0.00116	0.0011	0.0011	0.0013	0.0014	0.00187	0.0014	0.0019	0.0019	0.0023	0.002	0.001	0.0023	0.0005	0.00137	0.0017	0.00063	0.0002	0.0007	0.0007	8E-05	0.0016	0.0011
398	20.502	668748	234.1	Formononeti	0.0002	0.00022	0.0003	0.0003	0.0243	3E-05	2.7E-05	2E-05	7E-05	4E-05	0.0001	8E-05	3E-05	0.0001	0.0177	0.00608	3E-05	7.3E-05	6E-05	0.0178	3.6E-05	0.0145	3E-05	1E-05
399	20.557	668751	217.1	Ethyl beta-d-	0.0037	0.00416	0.0039	0.0043	0.0045	0.006	0.00653	0.008	0.008	0.0034	0.0047	0.0036	0.0038	0.0054	0.0064	0.08124	0.0071	0.00436	0.003	0.0022	0.0013	0.0025	0.0011	0.0012
400	20.594	668753	295.092	Pinitol	0.0015	0.00164	0.0014	0.0016	0.0017	0.0014	0.00119	0.0016	0.0018	0.001	0.002	0.0008	0.0018	0.0024	0.0011	0.00461	0.0012	0.00123	0.0011	0.0009	0.00075	0.0006	0.0007	0.0008
401	20.615	668754	147.1	Gluconic acid	0.024	0.02414	0.025	0.0253	0.0122	0.0146	0.01769	0.0151	0.0196	0.0165	0.0174	0.0105	0.0217	0.0197	0.0163	0.21973	0.0406	0.02253	0.0177	0.0181	0.00677	0.0098	0.0126	0.0096
402	20.615	668754	147.1	Isohexonic a	0.0245	0.02414	0.0286	0.0266	0.0148	0.0172	0.02005	0.0177	0.0225	0.0168	0.0181	0.0105	0.0234	0.0197	0.0163	0.21973	0.0427	0.02253	0.0189	0.0182	0.00706	0.01	0.0127	0.0096
403	20.712	668759	293.2	4-hydroxycin	0.0028	0.00313	0.0029	0.003	0.0057	0.0047	0.0029	0.0049	0.0028	0.0037	0.004	0.0046	0.0038	0.004	0.004	0.00517	0.0022	0.00379	0.0039	0.003	0.00333	0.0034	0.0028	0.0036
404	20.725	668760	333.2	Galactaric ac	0.0019	0.00196	0.002	0.0019	0.0015	0.0008	0.00118	0.0009	0.0012	0.0016	0.0018	0.001	0.0008	0.0018	0.001	0.02034	0.0011	0.00125	0.0011	0.0007	0.00058	0.0009	0.0009	0.0012
406	20.816	668765	217.1	D-glucuronic	0.0063	0.00634	0.006	0.0061	0.0038	0.0028	0.00494	0.0037	0.0059	0.0047	0.0049	0.0041	0.0053	0.0062	0.0044	0.04659	0.0085	0.00501	0.0042	0.0036	0.00499	0.003	0.0024	0.0038
407	20.855	668767	353.2	Xanthine	0.0018	0.00162	0.0016	0.0011	0.0007	0.0009	0.00038	0.0008	0.0005	0.001	0.0009	0.0013	0.0007	0.0011	0.004	0.00024	0.0016	0.0009	0.0005	0.0006	0.00088	0.0007	0.0159	0.0004
408	20.872	668768	393.187	Myo-inositol	0.0011	0.0011	0.0011	0.0011	0.0011	0.0004	0.00069	0.0005	0.0006	0.0015	0.0016	0.0007	0.0002	0.0017	0.0011	0.00445	0.0012	0.00066	0.0007	0.0005	0.00097	0.0004	0.0021	0.0012
411	20.943	668771	311.25	Palmitelaidic	0.0052	0.00508	0.0049	0.005	0.0028	0.001	0.00097	0.0001	0.0011	0.001	0.0009	0.0092	0.0021	0.001	0.0009	0.00167	0.0021	0.00101	0.0014	0.0037	0.00587	0.0038	0.0284	0.0022
414	21.092	668779	331.176	Alloxanic aci	0.0027	0.00307	0.0025	0.0025	0.004	0.0033	0.00289	0.0036	0.0033	0.0024	0.0032	0.0017	0.003	0.0032	0.0021	0.00278	0.0028	0.00319	0.0029	0.0014	0.00243	0.0017	0.0025	0.0029
415	21.146	668782	117.076	Palmitic acid	0.2696	0.26525	0.2404	0.2483	0.2367	0.1487	0.1249	0.1332	0.1161	0.2676	0.2638	0.4449	0.1693	0.2755	0.2456	0.12343	0.1682	0.34801	0.3818	0.2579	0.24194	0.1874	0.6051	0.165
417	21.234	668787	257.1	Abietic acid	0.0015	0.00171	0.0011	0.0013	0.0018	0.0009	0.00072	0.0011	0.0009	0.0015	0.0008	0.001	0.0008	0.0009	0.0016	0.00136	0.001	0.00133	0.0012	0.0036	0.00173	0.0008	0.0021	0.0022
420	21.332	668792	324.1	2-hydroxyhip	0.0045	0.00462	0.0044	0.0042	0.0019	0.001	0.00098	0.0009	0.0009	0.0039	0.0033	0.0029	0.0008	0.0039	0.0008	0.06177	0.0074	0.00535	0.0044	0.0008	0.00082	0.0008	0.0028	0.001
421	21.364	668794	357	Bisphenol a	0.2558	0.26056	0.2615	0.2594	0.2764	0.2848	0.2642	0.2839	0.2663	0.2689	0.2685	0.2502	0.2512	0.2627	0.2684	0.27282	0.2627	0.26434	0.2584	0.2274	0.26015	0.247	0.2547	0.2709
423	21.453	668798	217.1	Scyllo-inosito	0.1436	0.14664	0.1466	0.1471	0.1151	0.0714	0.0795	0.0759	0.0841	0.1235	0.1313	0.076	0.0851	0.1343	0.0997	1.67058	0.0925	0.08634	0.1074	0.079	0.05413	0.0674	0.1049	0.0877
424	21.488	668800	437.247	D-ribose-5-p	0.002	0.00205	0.0021	0.0021	0.0022	0.0029	0.0021	0.0026	0.002	0.0012	0.0011	0.0022	0.0037	0.0013	0.0019	0.00237	0.0028	0.00194	0.002	0.0007	0.00046	0.0007	0.0015	0.0013
426	21.561	668804	441.2	Uric acid	0.2697	0.28475	0.293	0.2962	0.4036	0.1879	0.2068	0.1973	0.2155	0.4037	0.4247	0.1868	0.1014	0.4315	0.5222	0.35772	0.3947	0.20756	0.2659	0.3178	0.15108	0.4641	0.2482	0.325
433	21.821	668818	229.147	Myristyl myr	0.004	0.00348	0.0037	0.0039	0.0062	0.0043	0.00416	0.0064	0.0058	0.0039	0.0059	0.0037	0.0063	0.0056	0.0036	0.0041	0.0042	0.00417	0.0043	0.0023	0.00358	0.0031	0.0044	0.0048
437	21.95	747426	294.188	7-methylguan	0.0043	0.00446	0.0043	0.0042	0.0019	0.0028	0.00535	0.0026	0.0054	0.0058	0.006	0.007	0.0016	0.0061	0.0028	0.01261	0.0041	0.00535	0.0056	0.0058	0.00143	0.0016	0.0028	0.0019
438	21.987	747428	327.276	Heptadecano	0.0025	0.0026	0.002	0.0022	0.0021	0.0018	0.00158	0.0016	0.0015	0.0022	0.0023	0.0042	0.0018	0.0026	0.002	0.00117	0.0018	0.00349	0.0033	0.0021	0.00205	0.0013	0.0041	0.0014
439	22.016	747430	243.1	6-hydroxy-al	0.0573	0.06557	0.0618	0.0604	0.0931	0.0927	0.06916	0.1018	0.0832	0.0802	0.1146	0.0801	0.0793	0.1306	0.0723	0.04721	0.059	0.05821	0.065	0.0264	0.05357	0.0177	0.0317	0.0482
445	22.174	747441	202.1	Indole-3-lact	0.0071	0.00774	0.0071	0.0075	0.0067	0.0056	0.00757	0.0056	0.0075	0.0082	0.0091	0.0076	0.0052	0.0088	0.0063	0.03	0.0142	0.0069	0.0069	0.0042	0.00397	0.0057	0.0051	0.0044
446	22.192	747442	315.195	Uridine 5'-m	0.0056	0.00557	0.0056	0.005	0.0101	0.0074	0.00598	0.0102	0.0087	0.0053	0.008	0.006	0.01	0.0073	0.0052	0.00645	0.0056	0.00754	0.0076	0.0023	0.00436	0.0033	0.006	0.0062
448	22.277	747448	401.218	D-fructose-1	0.001	0.00087	0.0009	0.0009	0.001	0.0009	0.00058	0.0005	0.0004	0.001	0.0002	0.0007	0.0004	0.0003	0.0005	0.00184	0.0007	0.00138	0.0008	0.0012	0.00066	0.0006	0.0007	0.0012
451	22.327	747451	174.1	Glycyl tyrosi	0.0075	0.00717	0.0069	0.0076	0.0061	0.0061	0.006	0.0052	0.005	0.0052	0.0054	0.0064	0.0049	0.0065	0.0046	0.01073	0.0102	0.00989	0.0089	0.0047	0.00425	0.0032	0.0044	0.0086
454	22.408	747457	248.083	Cholic acid	0.0015	0.00124	0.0012	0.0011	0.001	0.001	0.00128	0.0006	0.0007	0.0006	0.0003	0.0008	0.001	0.0004	0.0008	0.00085	0.0009	0.00129	0.0002	0.0003	0.0005	0.0003	0.001	0.0006
456	22.435	747459	315.1	Inosine-5'-m	0.0036	0.00345	0.0032	0.0035	0.0054	0.0038	0.00271	0.0039	0.0031	0.0037	0.0042	0.0045	0.0037	0.0041	0.0038	0.00451	0.0035	0.00382	0.0035	0.0037	0.00266	0.0039	0.0052	0.0055
457	22.437	747459	342.196	D-myo-inosit	0.0061	0.00617	0.0057	0.0059	0.0091	0.0063	0.00453	0.0072	0.0048	0.0064	0.007	0.0076	0.006	0.0071	0.0063	0.00674	0.0055	0.00626	0.0058	0.0064	0.00443	0.007	0.0091	0.0096
459	22.495	747463	202.1	Linoleic acid	0.1402	0.15036	0.1498	0.1488	0.1726	0.211	0.16342	0.2483	0.1832	0.1976	0.2359	0.1553	0.0816	0.2303	0.2893	0.10919	0.1921	0.2001	0.1006	0.1602	0.13958	0.1588	0.1301	0.1195
462	22.542	747466	117.078	Oleic acid	0.1414	0.14106	0.1414	0.1403	0.1246	0.0458	0.03793	0.0461	0.0378	0.145	0.1443	0.2139	0.0872	0.1451	0.1416	0.05541	0.0694	0.23642	0.2759	0.0666	0.11882	0.1145	0.6947	0.0589
464	22.577	747468	320.193	6,7-dimethyl	0.0092	0.00975	0.0109	0.0117	0.0136	0.0076	0.01111	0.0117	0.0178	0.0044	0.0073	0.0028	0.0146	0.0086	0.0055	0.0065	0.0083	0.00823	0.0121	0.0029	0.00336	0.0036	0.0063	0.0028
467	22.622	747471	193.1	3-hydroxyhip	0.0034	0.00367	0.0034	0.0036	0.0025	0.0016	0.00172	0.0023	0.0011	0.0011	0.0017	0.0013	0.0023	0.0015	0.0014	0.05359	0.0019	0.0033	0.0018	0.0009	0.00075	0.0005	0.0102	0.0011
469	22.701	747477	202.1	N-acetyl-d-tr	0.0087	0.0096	0.0085	0.009	0.0076	0.0028	0.00252	0.0054	0.0032	0.0286	0.0322	0.017	0.0075	0.0307	0.007	0.00441	0.0075	0.00977	0.0062	0.0158	0.03005	0.0047	0.0228	0.01
470	22.729	747479	117.077	Stearic acid	0.1286	0.12461	0.0988	0.1089	0.1121	0.0859	0.07222	0.064	0.0594	0.1183	0.1126	0.1479	0.0629	0.1162	0.112	0.06028	0.0868	0.15969	0.1489	0.1137	0.10562	0.0858	0.1331	0.0762
472	22.797	747483	406.2	Xanthurenic	0.0009	0.00115	0.001	0.0008	0.0002	0.0002	0.00038	0.0002	0.0001	0.0002	0.0002	0.0001	0.0003	0.0002	0.0001	0.01284	0.0004	0.00027	0.0002	7E-05	7.2E-05	0.0001	7E-05	1E-05
476	22.895	747490	221.1	Alpha tocoph	0.0033	0.00363	0.0031	0.0038	0.0048	0.0032	0.00325	0.0049	0.0047	0.0022	0.0055	0.0021	0.0048	0.0054	0.0026	0.00248	0.0031	0.00297	0.0028	0.0018	0.00159	0.0012	0.002	0.0017
479	22.991	747496	387.171	D-fructose-1	0.0007	0.00066	0.0007	0.0006	0.0007	0.0009	0.00063	0.0009	0.0007	0.0004	0.0004	0.0006	0.0007	0.0004	0.0007	0.00102	0.0006	0.00079	0.0006	0.0003	0.00027	0.0004	0.0003	0.0003
481	23.027	747499	246.117	Lactobionic a	0.0055	0.0064	0.0064	0.0062	0.009	0.0069	0.00537	0.0095	0.0076	0.0034	0.0045	0.0059	0.0155	0.0042	0.0061	0.00552	0.0068	0.00698	0.0065	0.0014	0.00118	0.0016	0.0031	0.0032
482	23.029	747499	218.1	L-cystine	0.0108	0.00609	0.0121	0.0129	0.0169	0.0087	0.00992	0.0098	0.0082	0.0059	0.0089	0.0142	0.0204	0.0079	0.0176	0.04435	0.0068	0.01534	0.0075	0.0073	0.00353	0.0072	0.0043	0.0022
486	23.15	747507	387.2	Chlorogenic	0.0008	0.00079	0.0008	0.0007	0.0008	0.001	0.0008	0.0012	0.0009	0.0005	0.0006	0.0006	0.0008	0.0006	0.0009	0.00104	0.0006	0.00092	0.0006	0.0004	0.00011	0.0004	0.0004	0.0004
493	23.271	747516	217.1	Pseudo uridi	0.0405	0.04237	0.0404	0.0416	0.0367	0.0235	0.03635	0.0232	0.0359	0.0227	0.0231	0.0228	0.0363	0.0239	0.0233	0.20687	0.0495	0.04297	0.0494	0.0178	0.01723	0.019	0.026	0.0224
500	23.398	819625	343.1	Piceatannol	0.0008	0.00063	0.0008	0.0007	0.0008	0.0007	0.00086	0.0008	0.0009	0.0009	0.0011	0.001	0.0009	0.0009	0.0006	0.00081	0.0008	0.00066	0.0007	0.0003	0.00086	0.0005	0.0008	0.0008
505	23.569	819640	350.2	Arachidonic	0.0126	0.01504	0.0163	0.0156	0.0245	0.011	0.01689	0.0162	0.0227	0.0073	0.0103	0.0101	0.0293	0.0094	0.0079	0.00911	0.0116	0.01726	0.0167	0.0028	0.00447	0.0032	0.0099	0.0052
506	23.616	819644	313.2	Resveratrol	0.0492	0.04683	0.0467	0.0465	0.065	0.0598	0.05019	0.0658	0.0583	0.0523	0.0541	0.0488	0.0602	0.0464	0.0467	0.05181	0.053	0.0595	0.0568	0.021	0.05048	0.0385	0.05	0.0604
509	23.692	819651	327.271	Octadecanol	0.0008	0.00082	0.0008	0.0009	0.0007	0.0008	0.00041	0.0007	0.0006	0.0009	0.001	0.0008	0.0006	0.0009	0.0008	0.00036	0.0007	0.00086	0.0005	0.0007	0.00096	0.0006	0.0008	0.0007
510	23.711	819652	290.1	Linolenic aci	0.0035	0.00516	0.0052	0.0041	0.0088	0.0049	0.00458	0.0082	0.0081	0.0034	0.0062	0.0044	0.0091	0.0058	0.0038	0.00524	0.0047	0.00516	0.0045	0.0009	0.00232	0.0016	0.0031	0.0038
513	23.773	819658	318.2	Myo-inositol	0.0026	0.00275	0.0024	0.0023	0.002	0.0022	0.00181	0.0021	0.0017	0.0028	0.0025	0.0024	0.0022	0.0025	0.0025	0.00237	0.0019	0.0025	0.0019	0.0041	0.00292	0.0028	0.0036	0.0037
515	23.834	819663	116.1	Oleamide	0.5793	0.63588	0.6603	0.6091	0.8269	0.7736	0.7422	0.7794	0.7239	0.4849	0.559	0.4895	0.6855	0.5267	0.4659	0.63836	0.7027	0.84143	0.8554	0.2009	0.42095	0.3659	0.4939	0.6189

517	23.835	819663	290.2	Edetic acid	8.4924	8.77652	9.1224	8.8524	10.714	10.706	10.1142	10.69	10.156	6.6224	7.2537	6.691	9.3384	6.8589	6.4533	8.89391	9.9469	11.6821	11.698	2.1596	5.68967	4.4705	6.784	8.9986
519	23.931	819671	338.3	Guanosine	0.0111	0.01169	0.0127	0.0116	0.0134	0.0137	0.01384	0.0143	0.0146	0.0081	0.0131	0.0123	0.013	0.0131	0.0118	0.01296	0.0129	0.01477	0.0117	0.0084	0.00951	0.0073	0.0111	0.0104
522	24.003	819677	369.35	Arachidic aci	0.0073	0.00807	0.0035	0.0037	0.002	0.0052	0.00314	0.0014	0.0011	0.0053	0.0018	0.0047	0.0023	0.002	0.0049	0.00435	0.0045	0.00392	0.0035	0.0157	0.02134	0.0093	0.0095	0.0068
528	24.197	819694	492.287	1-methylader	0.0006	0.00063	0.0007	0.0009	0.002	0.0012	0.00073	0.0021	0.0014	0.0004	0.0011	0.0016	0.0047	0.0014	0.0005	0.00037	0.0007	0.00099	0.0009	0.0002	0.00013	5E-05	0.0009	0.0006
533	24.342	819707	202.1	N-acetylgala	0.0106	0.00887	0.0109	0.0087	0.0076	0.0066	0.00573	0.0043	0.0042	0.0179	0.0143	0.0103	0.0041	0.0154	0.0027	0.01186	0.0108	0.01237	0.0041	0.0084	0.01756	0.0029	0.0201	0.0146
537	24.503	886621	354.4	Mesalazine	0.3087	0.3132	0.3122	0.3186	0.3162	0.3138	0.31335	0.3197	0.319	0.3141	0.3264	0.3066	0.3173	0.3375	0.3199	0.31263	0.3135	0.31401	0.3169	0.3179	0.31092	0.2792	0.3049	0.3079
542	24.632	886634	217.1	Inosine	0.0022	0.00234	0.0023	0.0383	0.0059	0.0015	0.02604	0.002	0.0027	0.0152	0.0012	0.0449	0.0052	0.0017	0.0107	0.0051	0.026	0.00459	0.002	0.0024	0.00821	0.0065	0.0238	0.0142
543	24.633	886634	91.0833	Docosahexae	0.0057	0.00594	0.0053	0.0052	0.0027	0.0039	0.00317	0.004	0.0033	0.0079	0.0088	0.0325	0.0041	0.0094	0.0055	0.00243	0.004	0.0061	0.0047	0.0051	0.0045	0.003	0.0106	0.0031
546	24.737	886644	371.308	1-monopalmi	0.0165	0.01689	0.0163	0.0163	0.0177	0.0154	0.01421	0.0161	0.0152	0.0144	0.0155	0.0202	0.0199	0.0164	0.0174	0.01289	0.013	0.01324	0.0149	0.0179	0.01496	0.0131	0.0296	0.0173
547	24.767	886647	355.12	Epigallocate	0.0015	0.00117	0.0018	0.0015	0.002	0.0013	0.0014	0.0017	0.0016	0.0007	0.0017	0.0012	0.0012	0.0018	0.0016	0.00134	0.0024	0.00146	0.0007	0.0012	0.00234	0.0012	0.0005	0.0018
548	24.788	886650	370.3	Catechin	0.0008	0.00077	0.0007	0.0009	0.0007	0.0005	0.00051	0.0008	0.0007	0.0005	0.0013	0.0006	0.0005	0.0013	0.0004	0.00059	0.0006	0.00075	0.0006	0.0004	0.00037	0.0004	0.0004	0.0004
550	24.848	886656	217.1	Digalacturon	0.0126	0.01263	0.0111	0.011	0.0127	0.0075	0.01049	0.0081	0.0102	0.0075	0.0068	0.0116	0.0185	0.0059	0.0028	0.03118	0.0088	0.01214	0.007	0.0029	0.00274	0.0038	0.007	0.0054
552	24.923	886663	361.208	Sucrose	0.0095	0.00967	0.0091	0.01	0.0065	0.0062	0.01497	0.0058	0.015	0.002	0.0028	0.0051	0.0072	0.0026	0.0044	0.03342	0.0154	0.00406	0.0117	0.0143	0.00659	0.0024	0.0076	0.0179
553	24.923	886663	361.2	Trisaccharide	0.0095	0.00967	0.0096	0.01	0.0072	0.006	0.01497	0.005	0.015	0.0025	0.0028	0.006	0.0076	0.0026	0.0044	0.02887	0.0145	0.00406	0.0122	0.0143	0.00659	0.0038	0.0076	0.0182
555	25.001	886671	290.14	N-acetyl-5-hy	0.0249	0.03006	0.0395	0.029	0.0512	0.0301	0.03515	0.0504	0.0435	0.0166	0.0305	0.0243	0.0392	0.0286	0.0203	0.02974	0.0338	0.05434	0.0569	0.003	0.00671	0.0048	0.0157	0.0261
556	25.016	886673	397.35	Behenic acid	0.0015	0.00164	0.0009	0.0012	0.0008	0.0014	0.00075	0.0006	0.0004	0.001	0.0007	0.001	0.0008	0.0012	0.001	0.00086	0.001	0.00089	0.0008	0.0015	0.00165	0.0008	0.0017	0.001
560	25.279	886700	204.1	Melibiose	0.0179	0.01797	0.015	0.0122	0.0103	0.0092	0.0214	0.0085	0.0212	0.013	0.0127	0.0074	0.0225	0.0082	0.0127	0.05746	0.0247	0.02576	0.0238	0.0091	0.00705	0.008	0.0094	0.0245
564	25.438	886716	361.2	Trehalose	0.0281	0.02832	0.0291	0.0289	0.017	0.0186	0.08045	0.019	0.0817	0.0196	0.0205	0.08	0.0455	0.0215	0.0105	0.02493	0.0189	0.03011	0.0174	0.0122	0.01418	0.0124	0.012	0.0168
568	25.556	886728	397.3	Monooleoylg	0.0035	0.00367	0.0034	0.0034	0.0017	0.0005	0.0007	0.0007	0.0008	0.0017	0.0018	0.0047	0.0088	0.0017	0.0013	0.00113	0.0009	0.00119	0.0011	0.0016	0.00053	0.0012	0.0445	0.0011
570	25.65	886738	459.227	Cellobiose	0.0012	0.00089	0.0011	0.001	0.0013	0.0004	0.00037	0.0005	0.0007	0.0022	0.0022	0.0015	0.0009	0.0017	0.0007	0.00073	0.0007	0.00133	0.001	0.0008	0.00141	0.0004	0.0025	0.0018
571	25.67	886740	399.375	Glycerol mon	0.0092	0.0101	0.0085	0.0088	0.0089	0.0082	0.00767	0.0082	0.0084	0.0084	0.0089	0.0107	0.0085	0.009	0.011	0.00725	0.0067	0.00686	0.0078	0.0119	0.01032	0.0078	0.0124	0.0102
576	25.998	886773	277.1	Cerotinic aci	0.0017	0.0018	0.0017	0.0015	0.0003	0.0001	0.00032	0.0004	0.0004	0.0001	0.0003	0.0001	0.0003	0.0003	0.0002	0.03228	0.0004	0.00027	0.0003	3E-05	0.00018	0.0002	7E-05	5E-05
580	26.374	886811	474.375	Delta-tocoph	0.0009	0.00114	0.0009	0.001	0.0016	0.0007	0.00068	0.0008	0.0009	0.0012	0.0012	0.0021	0.001	0.0015	0.0005	0.00157	0.0006	0.00089	0.002	0.0008	0.00026	0.0003	0.0004	0.0014
582	26.521	886826	215.2	Dihydrochol	0.0019	0.00175	0.0014	0.001	0.0024	0.0015	0.00193	0.0022	0.0018	0.0011	0.0014	0.0015	0.003	0.001	0.001	0.00115	0.0008	0.00146	0.0013	0.001	0.00099	0.0019	0.0017	0.0014
583	26.615	886836	203.2	Lithocholic a	0.0009	0.00148	0.0004	0.0005	0.0005	0.0113	0.00046	0.0004	0.0007	0.0039	0.0021	0.0006	0.0005	0.0005	0.0026	0.0006	0.0005	0.00058	0.0005	0.0045	0.00274	0.0027	0.001	0.0029
586	26.936	886869	361.2	Melezitose	9E-05	0.00116	0.0007	0.0006	0.0001	0.0007	0.00023	8E-05	8E-05	4E-05	4E-05	2E-05	0.0002	6E-05	5E-05	0.0143	0.0002	0.00025	0.0002	2E-05	0.00011	1E-06	7E-05	8E-05
587	26.983	886874	488.5	Gamma-toco	0.0083	0.00924	0.0081	0.0083	0.0118	0.0092	0.0019	0.0101	0.0022	0.0065	0.0073	0.0203	0.0115	0.007	0.0043	0.01033	0.0043	0.00964	0.0121	0.0078	0.00597	0.0048	0.0063	0.0129
588	27.071	886883	227.057	Stigmasterol	0.0053	0.01139	0.0112	0.0047	0.0173	0.009	0.00991	0.0126	0.0163	0.0058	0.0088	0.0147	0.0185	0.0091	0.0067	0.01259	0.0061	0.01232	0.0121	0.005	0.0028	0.0017	0.0044	0.0063
589	27.15	886891	243.083	Beta-glycero	0.0056	0.01558	0.0062	0.0126	0.014	0.0096	0.00885	0.0144	0.0093	0.0158	0.024	0.0244	0.0135	0.0261	0.0162	0.01244	0.0108	0.01222	0.0046	0.0125	0.01005	0.006	0.0173	0.02
590	27.161	886892	357.129	2-deoxy-d-er	0.01	0.01277	0.0081	0.01	0.0103	0.0079	0.00571	0.011	0.005	0.013	0.017	0.0187	0.0119	0.02	0.0134	0.01049	0.0057	0.0096	0.0068	0.0105	0.0072	0.005	0.0136	0.014
592	27.288	886905	315	Adenosine-5'	0.0006	0.00084	0.0002	0.0002	0.0002	0.0001	9.5E-05	0.0003	0.0002	0.0003	0.0002	0.0006	0.0008	0.0003	0.0002	0.00035	0.0002	0.00028	0.001	1E-04	0.00017	2E-05	0.0017	0.0002
593	27.503	886927	203.2	Ursolic acid	0.0045	0.00552	0.0031	0.0024	0.0013	0.0269	0.00495	0.0011	0.0018	0.0072	0.0025	0.0033	0.0018	0.0005	0.0061	0.00335	0.0032	0.00478	0.0028	0.0126	0.00675	0.0058	0.0041	0.004
595	28.042	886982	237.14	Alpha-tocop	0.0243	0.02662	0.0243	0.0248	0.024	0.0373	0.04533	0.0391	0.0473	0.0217	0.0235	0.0251	0.0174	0.0233	0.0354	0.02324	0.0356	0.02934	0.0076	0.0283	0.02393	0.0169	0.0293	0.0209
596	28.228	887001	129.1	Cholesterol	0.7002	0.68556	0.6968	0.6934	0.7802	0.7871	0.58274	0.8093	0.6014	0.6869	0.6996	0.7539	0.7795	0.701	0.8031	0.56315	0.6343	0.79271	0.6445	0.6827	0.71606	0.5564	0.7625	0.7373
597	28.776	887057	458.4	Lathosterol	0.0004	0.0005	0.0004	0.0004	0.0006	0.0004	0.00029	0.0004	0.0003	0.0002	0.0003	0.0009	0.0002	0.0002	0.0018	0.00035	1E-06	0.00021	1E-04	0.0005	0.00034	0.0002	0.0004	0.0009
598	29.386	887119	255.2	Deoxycholic	0.0012	0.00137	0.0011	0.0011	0.0006	0.0013	0.0001	0.0011	0.0001	0.001	0.0011	0.0005	0.0144	0.0011	0.0007	0.00053	0.0002	0.00118	0.0011	0.0001	0.00095	0.0004	0.0002	0.0001
599	29.708	887152	428.3	Chenodeoxy	0.0011	0.00124	0.0011	0.0011	0.0001	3E-05	1.8E-05	0.0002	8E-05	1E-06	4E-05	0.0007	0.0219	7E-05	0.0001	0.00063	0.0004	0.00065	0.0002	0.0007	4.9E-05	3E-05	0.0004	0.0002
600	29.726	887153	311.3	Labetalol	0.0376	0.04355	0.046	0.0356	0.0472	4E-05	0.05065	0.052	0.0646	0.0403	0.0572	0.0414	0.0524	0.0362	0.0434	0.06164	0.0343	0.04371	0.0482	0.0394	0.03235	0.0334	0.033	0.0401
601	29.856	887167	309.271	Beta-sitoster	0.0053	0.00727	0.0081	0.0068	0.007	3E-05	0.00984	0.0072	0.0117	0.0072	0.0076	0.007	0.0068	0.006	0.0077	0.0101	0.0052	0.00746	0.0099	0.0067	0.0079	0.0055	0.0042	0.008
602	29.911	887172	495.6	Triacontanol	0.001	0.00191	0.0006	0.0008	0.0001	0.0007	0.00043	6E-05	8E-05	0.0004	2E-05	0.0003	6E-05	0.0002	0.0008	0.00069	0.0007	0.00058	0.0004	0.0008	0.00088	0.0005	0.0005	0.0005
